# *ChARM*: Discovery of combinatorial chromatin modification patterns in hepatitis B virus X-transformed mouse liver cancer using association rule mining

**DOI:** 10.1186/s12859-016-1307-z

**Published:** 2016-12-13

**Authors:** Sung Hee Park, Sun-Min Lee, Young-Joon Kim, Sangsoo Kim

**Affiliations:** 1 Department of Bioinformatics and Life Science, Soongsil University, Seoul, 156-743 Republic of Korea; 2 Department of Biochemistry, College of Life Science and Technology, Yonsei University, Seoul, 120-749 Republic of Korea; 3 Department of Integrated Omics for Biomedical Science, World Class University Program, Yonsei University, Seoul, 120-749 Republic of Korea

**Keywords:** Combinatorial histone modifications, Association rule mining, Differential modifications, Chromatin signature, Hepatitis B virus X (HBx)-transgenic mice, Hepatocellular carcinoma

## Abstract

**Background:**

Various chromatin modifications, identified in large-scale epigenomic analyses, are associated with distinct phenotypes of different cells and disease phases. To improve our understanding of these variations, many computational methods have been developed to discover novel sites and cell-specific chromatin modifications. Despite the availability of existing methods, there is still room for further improvement when they are applied to resolve the histone code hypothesis. Hence, we aim to investigate the development of a computational method to provide new insights into *de novo* combinatorial pattern discovery of chromatin modifications to characterize epigenetic variations in distinct phenotypes of different cells.

**Results:**

We report a new computational approach, *ChARM* (*C*ombinatorial C*h*romatin Modification Patterns using *A*ssociation *R*ule *M*ining), that can be employed for the discovery of *de novo* combinatorial patterns of differential chromatin modifications. We used *ChARM* to analyse chromatin modification data from the livers of normal (non-cancerous) mice and hepatitis B virus X (HBx)-transgenic mice with hepatocellular carcinoma, and discovered 2,409 association rules representing combinatorial chromatin modification patterns. Among these, the combination of three histone modifications, a loss of H3K4Me3 and gains of H3K27Me3 and H3K36Me3, was the most striking pattern associated with the cancer. This pattern was enriched in functional elements of the mouse genome such as promoters, coding exons and 5′UTR with high CpG content, and CpG islands. It also showed strong correlations with polymerase activity at promoters and DNA methylation levels at gene bodies. We found that 30 % of the genes associated with the pattern were differentially expressed in the HBx compared to the normal, and 78.9 % of these genes were down-regulated. The significant canonical pathways (Wnt/ß-catenin, cAMP, Ras, and Notch signalling) that were enriched in the pattern could account for the pathogenesis of HBx.

**Conclusions:**

*ChARM*, an unsupervised method for discovering combinatorial chromatin modification patterns, can identify histone modifications that occur globally. *ChARM* provides a scalable framework that can easily be applied to find various levels of combination patterns, which should reflect a range of globally common to locally rare chromatin modifications.

**Electronic supplementary material:**

The online version of this article (doi:10.1186/s12859-016-1307-z) contains supplementary material, which is available to authorized users.

## Background

Post-translational histone modifications are known to be altered in cancer tissues and to contribute to the development and progression of cancer [1–3]. Histone modifications can occur over large regions of chromatin, including coding regions and non-promoter sequences, and these are referred to as global histone modifications [4–6]. Mutations, mis-regulation of gene expression, or attenuated post-translational modifications can impair the activity of histone-modifying enzyme complexes, and this may affect the mechanism that regulates global histone modifications throughout the genome. Currently, the consequences of altered histone-modifying enzyme activity are linked to inappropriate expression of a few genes that might function in tumorigenesis [4, 7]. Changes in global histone modification patterns (GHMPs) can be informative, particularly as predictors of prognosis, various steps of carcinogenesis, and responses to chemotherapy [4, 8]. Therefore, in this study, we aim to develop a new data mining approach for discovery and interpretation of differential GHMPs in cancer, and we use it to investigate whether an understanding of epigenetic alterations in cancer cells can expand prognostic capabilities.

For GHMPs, the complexity of patterns discovered can be explained by using the histone code hypothesis [8, 9], which states that each of four histones can be simultaneously modified in a site specific manner with different degrees of change in different modifications. For an example of this complexity, consider the following: histone H3 contains 19 lysine residues known to be methylated, and each lysine can be un-, mono-, di-, or tri-methylated. If modifications are considered to be independent, this allows a potential 4^19^ or 280 billion different lysine methylation patterns, which is more than the maximum number of histones in the human genome (~44 million) [10]. In this context, the problem of discovering the combinatorial chromatin modification patterns (CCMPs) that exist on a genome-wide scale can be considered an NP-complete (nondeterministic polynomial time complete) problem [11]. To date, various computational methods based on heuristic algorithms have been developed for the identification of GHMPs and CCMPs. Additionally, with the recent advances in next generation sequencing technology, new computational methods exploiting machine learning and data mining algorithms are being developed to detect histone modification patterns in genome-wide chromatin immunoprecipitation (ChIP)-Seq data sets. For instance, supervised learning based methods can identify and predict functional DNA elements (enhancers, promoters, and insulators) with chromatin signatures for known regulatory elements using classification algorithms such as artificial neural networks [12] and hidden Markov models (HMMs) [13]. The advantage of these supervised methods is their ability to predict the undiscovered regulatory elements that drive cell-type-specific gene expression.

Un-supervised learning algorithms can be applied to identify GHMPs and discover novel CCMPs that can characterise unknown regulatory elements. A range of algorithms are adapted to achieve this, including probabilistic profiles (e.g. ChromaSig [14]), bi-clustering (CoSBI [15] and SS-CoSBI [16]), HMMs (ChromHMM [17]), dynamic Bayesian networks ([18] and SegWay [19]), and dynamic programming (ChAT [20]). In contrast to some other unsupervised methods (e.g. ChromaSig, CoSBI, and ChAT), ChromHMM and SegWay, which segment the genome into distinct chromatin states, are advantageous to identify patterns of sequential chromatin modifications (spatially separate patterns), and the final CCMPs are forced to include all chromatin modification marks in the input data [16]. ChromHMM and SegWay focus on chromatin-centric genome annotations in order to assign and predict the final labels of chromatin states for given genome segments with chromatin marks. ChAT can discriminate the same combinatorial patterns of histone modifications with different shapes by using dynamic programming to measure the similarity of the chromatin signatures for genome partitions, but it may capture local signatures rather than those that occur globally.

Despite the development of many computational methods designed to elucidate combinatorial patterns of histone modifications and decipher the complex histone code, how CCMPs can be incorporated into the elaborate epigenetic model of cancer in contrast to normal cells has not been determined. Large-scale epigenomic projects have generated a vast number of epigenomes, including various types of histone modification and other epigenetic marks, for multiple human cell types and disease progression [21–23]. However, the development of computational methods for discovery of combinatorial patterns of chromatin state differences between different cell types and conditions has not been investigated. The discovery of *de novo* combinatorial patterns of differential chromatin modifications across tissues, cell types, and disease phases, is a non-trivial task. The validity of such a computational method can be determined by assessing its ability to extract novel biological knowledge from the patterns associated with various functional genomic features.

In this regard, we report *ChARM*, a new computational approach based on association rule mining (ARM), which is *de novo* pattern discovery of differential chromatin modifications that occur globally in hepatocellular carcinoma (HCC) tissues of hepatitis B virus X (HBx)-transgenic mice. *ChARM* computationally characterises these patterns to interpret their biological significance. By applying ARM to three different types of histone lysine methylation, DNA methylation, and RNA polymerase II (Pol II) phosphorylation on a genome-wide scale, we discovered 2,409 association rules that were expressed as combinatorial patterns of differential chromatin modifications. We identified a cancer-specific *de novo* global pattern, i.e. the combination of three histone modifications, namely a loss of H3K4Me3 and gains of H3K27Me3 and H3K36Me3, in both promoters and gene bodies. *ChARM* is an unsupervised approach for incorporating global CCMPs into epigenetic models of cancer, providing combinatorial patterns that discriminate HBx and normal (non-cancerous) tissue. The patterns are expressed with descriptive rules that are straightforward and simple to interpret.

## Results

### A global view of the discovered association rules

**Fig. 1:**
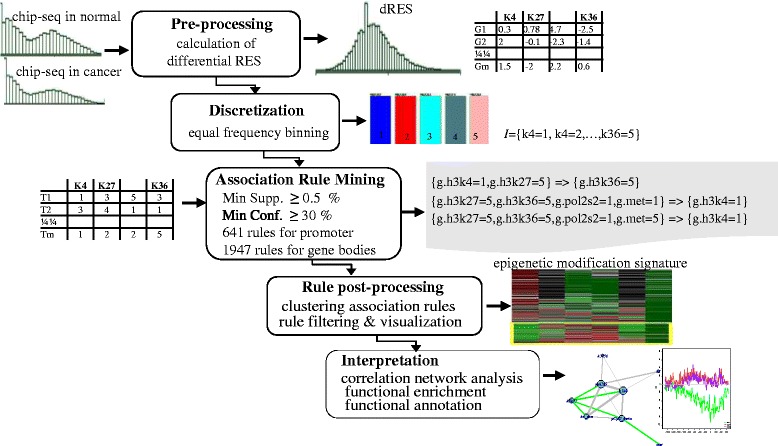
A work flow diagram of the *ChARM* method

An overall systematic workflow of the CCMP discovery process is shown in Fig. 1. This comprises transformation of our ChIP-seq data from continuous to categorical, ARM of the transformed ChIP-seq data, and clustering of association rules for the visualization and interpretation of patterns (Fig. 1). ARM was applied to promoter and gene body regions separately. All the association rules exceeded the thresholds of supports, confidence, and lifts were generated. In total, 556 rules (see Additional file 1: Table S1) for promoters and 1,853 rules (see Additional file 1: Table S2) for gene bodies (minimum support > 0.005, minimum confidence ≥ 0.3, Table 1) were discovered by the CCMP procedure described in Fig. 1.

**Table 1:** Representative association rules

No	Rule description for promoter	Supp^a^	Conf^b^	Lift	Annotation^c^
1	p.h3k27 = 5 p.h3k36 = 5 == > p.h3k4 = 1	0.018	0.35	1.87	P155
2	p.h3k4 = 1 p.h3k36 = 5 == > p.h3k27 = 5	0.018	0.34	1.74	P155
3	p.h3k4 = 1 p.h3k27 = 5 == > p.h3k36 = 5	0.018	0.33	1.80	P155
4	p.h3k27 = 5 p.h3k36 = 5 p.pol2s5 = 1 == > p.h3k4 = 1	0.005	0.41	2.17	Super set & highest lift
5	p.h3k4 = 1,p.h3k27 = 5,p.h3k36 = 5 = > p.met = 2	0.007	0.39	1.05	Super set & Lowest lift
6	p.h3k27 = 5 p.h3k36 = 5 p.met = 2 == > p.h3k4 = 1	0.007	0.36	1.89	Super set & Top 5 lift
7	p.h3k4 = 1 p.h3k27 = 5 p.met = 2 == > p.h3k36 = 5	0.007	0.33	1.8	Super set & Top 5 lift
8	p.h3k4 = 1 p.h3k36 = 5 p.pol2s5 = 1 == > p.h3k27 = 5	0.005	0.34	1.7	Super set & Top 10 lift
9	p.h3k4 = 1 p.h3k27 = 5 p.pol2s5 = 1 == > p.h3k36 = 5	0.005	0.33	1.77	Super set & Top 10 lift
10	p.h3k4 = 4,p.h3 = 3,p.h3k27 = 2,p.h3k36 = 2,p.met = 2 = > p.pol2s5 = 3	0.008	0.80	4.46	Top 5 lift
11	p.h3k4 = 4,p.h3 = 3,p.h3k27 = 2,p.h3k36 = 2 = > p.pol2s5 = 3	0.010	0.73	4.09	Top 5 lift
12	p.met = 2	0.373	0.37	1	Top 5 support
13	p.pol2s5 = 1 = > p.met = 2	0.084	0.4	1.07	Top 5 support
14	p.h3k27 = 3 = > p.met = 2	0.083	0.36	0.98	Top 5 support
	Rule description for gene body				
15	g.h3k27 = 5 g.h3k36 = 5 == > g.h3k4 = 1	0.048	0.56	2.58	G155
16	g.h3k4 = 1 g.h3k27 = 5 == > g.h3k36 = 5	0.048	0.54	2.88	G155
17	g.h3k4 = 1 g.h3k36 = 5 == > g.h3k27 = 5	0.048	0.53	2.86	G155
18	g.h3k4 = 1,g.h3k27 = 5,g.pol2s2 = 1,g.met = 5 = > g.h3k36 = 5	0.006	0.66	3.54	Super set & Top 5 lift
19	g.h3k4 = 1,g.h3 = 5,g.h3k36 = 5,g.met = 1 = > g.h3k27 = 5	0.005	0.65	3.49	Super set & Top 5 lift
20	g.h3k4 = 1,g.h3k36 = 5,g.met = 1 = > g.h3k27 = 5	0.017	0.64	3.45	Super set & Top 5 lift
21	g.h3k4 = 1,g.h3k36 = 5,g.pol2s2 = 1,g.met = 1 = > g.h3k27 = 5	0.007	0.648	3.42	Super set & Top 5 lift
22	g.h3k4 = 1,g.h3 = 1,g.h3k36 = 5,g.met = 1 = > g.h3k27 = 5	0.006	0.63	3.4	Super set & Top 5 lift
23	g.h3k4 = 1,g.h3k27 = 5,g.h3k36 = 5,g.met = 1 = > g.h3 = 5	0.0053	0.328	1.55	Super set & the lowest lift
24	g.h3 = 3,g.h3k27 = 3,g.pol2s2 = 4,g.met = 2 = > g.h3k36 = 4	0.008	0.79	3.86	Top 5 lift
25	g.h3 = 3,g.h3k27 = 3,g.h3k36 = 4,g.pol2s2 = 4,g.met = 2 = > g.h3k4 = 4	0.005	0.64	3.68	Top 5 lift
26	g.h3k36 = 5 = > g.h3k4 = 1	0.089	0.487	2.19	Top 5 support
27	g.h3k4 = 1 = > g.h3k36 = 5	0.089	0.41	2.19	Top 5 support
28	g.h3k27 = 5 = > g.h3k4 = 1	0.088	0.47	2.18	Top 5 support
29	g.h3k4 = 1 = > g.h3k27 = 5	0.088	0.41	2.18	Top 5 support
30	g.h3k27 = 5 = > g.h3k36 = 5	0.085	0.45	2.43	Top 5 support
31	g.h3k36 = 5 = > g.h3k27 = 5	0.085	0.45	2.43	Top 5 support

There were 556 rules and 1853 rules discovered by ARM for promoters and gene bodies, respectively. From these rules, we selected those encoding Pattern 155 (Rule 1–3 and Rule 15–17) and its supersets with high lift values, which were within the top 5 or top 10 highest lift values from all the rules as representative examples. In the table, we also report rules in the top 5 supports

^a^Supp: Support of a rule

^b^Conf: Confidence of a rule

^c^Annotation: annotation of the rules corresponding to their categories

**Fig. 2:**
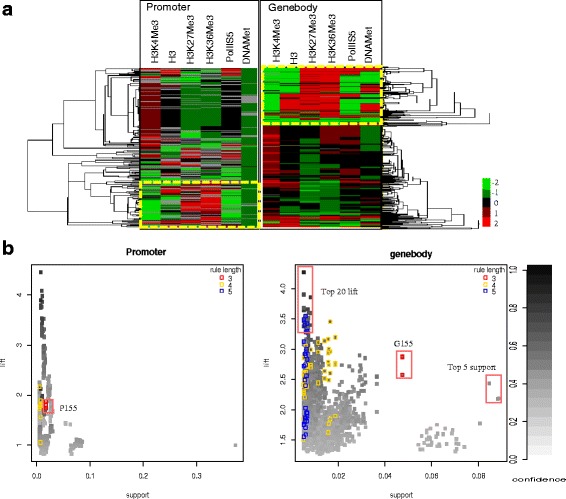
A global view of the chromatin modification patterns encoded in association rules. **a** All epigenetic signatures of chromatin modifications and Pattern 155. Each row corresponds to an association rule (i.e. a pattern or a combination of chromatin modification) and each column to a chromatin modification mark. Rules (556 for promoters and 1852 gene bodies) are clustered by chromatin modification marks. The colour in each cell indicates the differential change of marks in the livers of normal and HBx-transgenic mice. Light green and light red represent the extreme chromatin modification changes, e.g. hypo or hyper methylation of histone, respectively. The epigenetic signatures were tightly clustered into two groups, representing the modified and unmodified states of chromatin. Association rules in the yellow rectangles represent the epigenetic signatures of Pattern 155, which constitutes the combination of the loss of H3K4Me3 and the gains of H3K27Me3 and H3K36Me3. **b** Plots for the support and lift of the association rules. The grey scale represents the confidence levels and the coloured rectangles correspond to supersets of Pattern 155, which contained the three modified states of Pattern 155 as well as other chromatin marks. The rule length, which corresponds to the number of modified states, is > 3. Red, yellow, and blue rectangles correspond to rule length 3, 4, and 5, respectively

To extract and interpret interesting CCMPs from all the discovered rules, we employed existing tools such as TreeView and Gene Cluster 3.0 to produce a heatmap representing the global view of all the association rules. This heatmap, clustered by chromatin modification marks, represents the combinatorial effects of chromatin modification states (Fig. 2a). Each association rule (i.e. each row in Fig. 2a) encodes a pattern or signature of the combination of differentially modified states of chromatin. High support values can indicate globally modified patterns, and high lift values can signify the degree of co-occurrence. We filtered out rules presenting combinations of all unmodified states. The remaining rules were sorted by support and lift. Sorting by support has the same effect as rules were clustered by number of modifications in rules. After sorting by these two metrics, interesting rules were easily explored.

A combination of three histone modified states (H3K4Me3 = 1, H3K27Me3 = 5, and H3K36Me3 = 5) showed the highest frequency (support count = 957) among all possible combinations (125) in the gene bodies (Additional file 2: Figure S1), and it was the *K*-th most frequent itemset (*K* = 3), where *K* stands for the number of different chromatin modified states in a combination. Three association rules (Rules 15 ─ 17 in Table 1) were derived from this frequent itemset. In Fig. 2a, association rules in dashed yellows rectangles encode the notable combinations of differentially modified states that were derived from all possible subsets or supersets of the most frequent itemset.

In promoters, the combinations of unmodified states were common and present comprising a majority of the association rules with high frequency (e.g., Rules 12 ─ 14). The combination of three histone modified states (i.e. H3K4Me3 = 1, H3K27Me3 = 5, and H3K36Me3 = 5) in the promoter was the *K*-th most frequent itemset (*K* = 3) after filtering the combinations of any three unmodified states (Additional file 2: Figure S2). Thus, we identified a global histone modification pattern, named Pattern 155, which denotes the combinatorial effect of the loss of H3K4Me3 and the gains of H3K27Me3 and H3K36Me3 (Fig. 2a and Table 1). Pattern 155 was discovered in both promoters (named P155 for promoter pattern, Additional file 1: Table S3) and gene bodies (G155 for gene body pattern, Additional file 1: Table S4). Table 1 also lists a variety of other rules that form parts of the supersets and subsets of Pattern 155. We refined Pattern 155 to reduce potential false positives and derived fine patterns (Additional file 1: Table S5). Lift was employed to measure the independence of a rule and as a metric for the importance of a rule in terms of measuring co-occurrence of chromatin modifications. The lift values of the association rules encoding Pattern 155 were high (>1.5) as shown in Rules 1–3 and Rules 15–17 (Table 1). With the exception of two rules (Rule 5 and Rule 23), the supersets of Pattern 155 (i.e. the coloured rectangles in Fig. 2b, Rules 4–9 for promoters and Rules 18–23 for gene bodies), which consisted of more than three chromatin modified states, had higher lift values than the original Pattern 155 (1.736 ≤ lift ≤ 2.17 for promoter; 3.42 ≤ lift ≤ 3.54 for gene body).

It is notable that 55 (79 %) of 70 supersets (3.8 %, Additional file 1: Table S6) from G155 had high lift values (≥2) and the top 5 lifts of these supersets ranked within the top 1 % of highest lifts of all the association rules (*P* = 5.2 × 10^−4^). The high lift values of these supersets shows that the combination of three histone methylation marks (H3K4Me3 = 1, H3K36Me3 = 5, H3K27Me3 = 5) in Pattern 155 appeared together more often than expected, and that the pattern more likely co-occurred with other modifications such as RNA polymerase changes and DNA methylation. As more chromatin marks combined, the lift tended to increase (Additional file 2: Figure S3), implying a higher possibility for co-occurrence. These results suggest the possibility of interplay between three histone methylation marks in the pattern, which results from cross-talks between trimethylation of lysine 4, lysine 27, and lysine 36.

Except for rules in Table 1 and Pattern 155, we found a promoter pattern representing the combination of four modifications (i.e. H3K27me3 = 1, H3K36me3 = 2, DNY Methylation = 2, H3K4me3 = 4) but that mainly denotes loss of H3K27me3 and gain of H3K4me3. We also identified other patterns such as Pattern 511 and Pattern 111 in gene bodies. Pattern 511 presents the combination of three modified states (i.e. gain of H3K4me3 and losses of H3K36me3 and H3K27me3). Supersets and subsets of Pattern 511 are in 102 rules. Pattern 111 denotes losses of all three histone modifications and encoded in 100 rules.

### Negative relationships between H3K27Me3 and other marks characterise Pattern 155

**Fig. 3:**
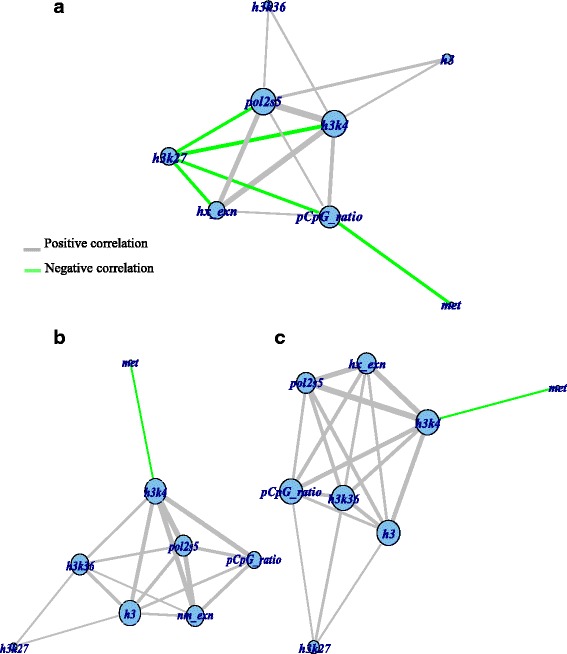
Correlation network of epigenetic modifications. The correlation networks were generated from correlations (r ≥ 0.2) between chromatin modification marks of transcripts in (**a**) P155, the promoter pattern for the HBx TG livers, **b** normal cells, and (**c**) HBx TG liver cells. Each node represents a chromatin modification mark and each edge width was weighted by Pearson correlation values (*r*). Green edges represent negative correlations and grey edges represent positive correlations. Each node name represents the abbreviated chromatin mark name: h3k4, h3k27, h3k36, pol2s5, met, h3, hx_exn, and pCpG_ratio denote H3K4Me3, H3K27M3, H3K36Me, Pol II S5, DNA methylation, H3, expression in HBx, and CpG ratio, respectively

The relationships among the epigenetic marks in Pattern 155 were compared to those of all other genes in either HBx-transformed cells or normal cells by using correlation network analysis based on the RESs (relative enrichment score) of ChIP-seq signals. Interestingly, we observed negative relationships between H3K27Me3 and other marks in Pattern 155 (Fig. 3a for P155s; Additional file 2: Figure S4A for G155s), but not among all genes in either normal or HBx-transformed liver cells (Fig. 3b and c, and Additional file 2: Figure S4B and C). Specifically, H3K27Me3 changes were negatively correlated with H3K4Me3, Pol2S5, gene expression, and CpG content in Pattern 155 (with the exception of the relationship with CpG content in G155s). These negative relationships are not replicated in random samples and had a low probability of occurring by chance (*P* < 10^−6^). Therefore, it is possible that the negative relationships between H3K27Me3 and other marks significantly affect epigenetic modifications in HBx.

In Pattern 155, the CpG content was negatively associated with DNA methylation and H3K27Me3 in P155s, while it had a weak positive relationship with H3K27Me3 in G155s (Additional file 2: Figure S4A). These negative relationships differ from the positive relationships observed for both H3K27Me3 and H3K36Me3 in HBx transformed cells for all genes, whereas such positive relationships were either not observed or were weak (with H3K36Me3 for gene bodies) in normal cells. The negative relationships observed between H3K27Me3 and other marks in Pattern 155 are compatible with the results of ARM (Rules 4, 8, and 9 in promoters; and Rules 18–23 in gene bodies; Table 1), and they can be considered as HBx-specific epigenetic modifications.

### Enrichment of functional genomic elements in Pattern 155

We measured the enrichment of the functional elements associating Pattern 155 in terms of odds relating to the relative proportion of functional elements in the mouse genome. The relative enrichment between the functional elements was then calculated as odd ratios (Table 2). In the pattern, there was a propensity for genes to non-genes (OR = 4.97), promoters to genes (OR = 1.05), exons to introns (OR = 3.58), and UTR5 to UTR3 (OR = 2.12). Interestingly, the pattern was enriched in genic regions and promoters rather than non-genic regions, and preferentially matched within exons with particularly coding regions and UTR5s.

**Table 2:** Enrichment of functional elements in the patterns

Functional elements	Mouse genome (MG) BP^a^	Ratio (MG)^b^	Patterns (P:P0) BP^c^	Ratio (P)^d^	Odds (M)^e^	Odds (P)^f^	Odds ratio (P/MG)^g^
Mouse genomes	2,725,765,481		12,537,400				
Non-gene	1,687,863,859	0.619	5,268,326	0.420	1.626	0.725	0.446
Promoter	60,956,000	0.022	636,400	0.051	0.023	0.053	2.338
Genes	976,945,622	0.358	6,936,145	0.553	0.559	1.238	2.217
Introns	917,470,255	0.337	6,319,444	0.504	0.507	1.016	2.003
Exons	63,877,330	0.023	1,841,546	0.147	0.024	0.172	7.175
Coding Exons	34,016,873	0.012	1,424,442	0.114	0.013	0.128	10.143
5′-UTR	6,222,075	0.002	211,139	0.017	0.002	0.017	7.487
3′-UTR	24,574,772	0.009	389,841	0.031	0.009	0.032	3.527

All of the 200 base pair intervals (62,687 intervals identified by a genome-wide scan) that met the conditions of the P155 pattern for promoters were mapped to the functional elements of the mouse genome

^a, b^Base pairs of functional elements in the mouse genome and their ratio over the mouse genome

^c, d^Base pairs of functional elements overlapping with the 200 base pair intervals in the pattern and their ratio over the pattern

^e, f^Odds for each functional element in the mouse genome and Pattern 155, calculated by Eq. 1

^g^Odds ratio for each functional element between the pattern and the mouse genome, representing functional element enrichment in the pattern in comparison to the mouse genome

### Epigenetic profiles of genes in Pattern 155

**Fig. 4:**
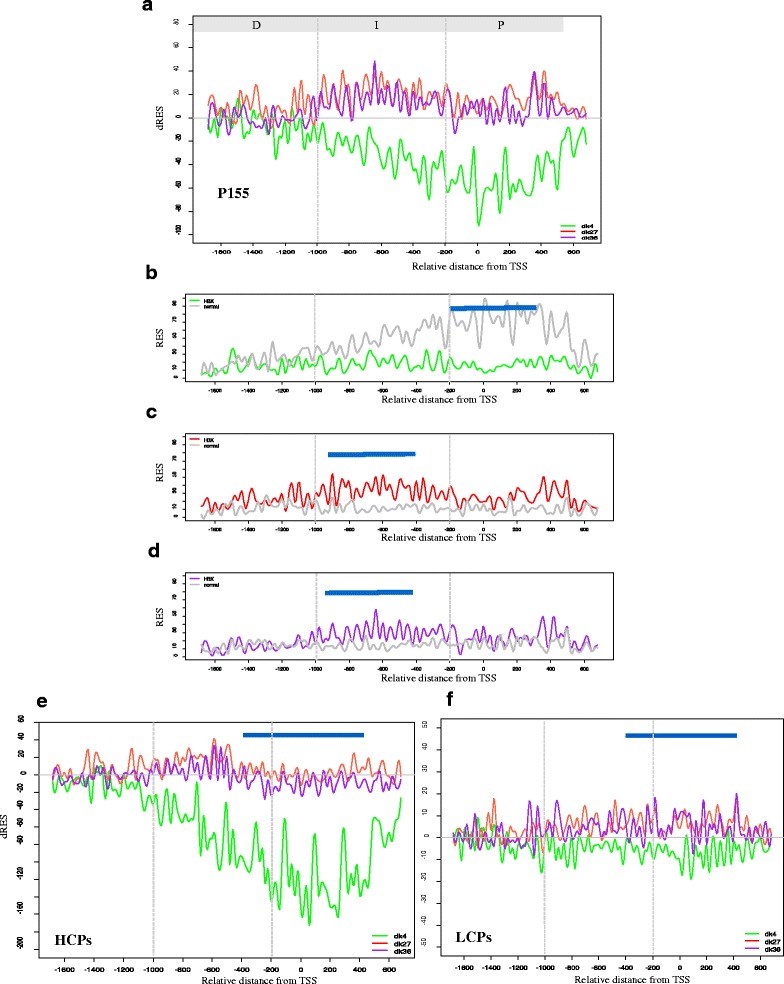
Epigenetic profiles of P155. **a** Differential changes of histone modifications between HBx TG and normal livers in Pattern 155. The plotted data are the dRES values summed over the member genes of Pattern 155 (50-bp interval). Promoter regions are divided into three regions relative to TSS: proximal (P: −200 to 500 bp), intermediate (I: −1000 to −200 bp) and distal (D: −1500 to −1,000 bp). *Vertical grey lines* in each figure represent the three promoter regions. **b**, **c** and **d**) A comparison between HBx and normal livers for (**b**) H3K4Me3, **c** H3K27Me3, and (**d**) H3K36Me3. **b** shows that H3K4Me3 was hypermethylated near the TSS regions in normal livers, whereas it underwent demethylation in HBx, displaying a strong negative peak in (**a**). **e** and **f** The changes in histone modification for (**e**) HCPs (242 transcripts) and (**f**) LCPs (43 transcripts) in Pattern 155. *Blue bars* in represent regions matched with Pattern 155. dRES changes of H3K4Me3, H3K27Me3 and H3K36Me3 from (**a**), (**e**) and (**f**) are coloured green, red, purple, respectively. dk4, dk27 and dk36 stand for H3K4Me3, H3K27Me3 and H3K36Me3, respectively

The average epigenetic profiles of the genes in P155 were measured in terms of RES values around the transcription start site (TSS). In normal cells, the P155 genes showed a strong H3K4Me3 peak around the TSS (the region from −200 bp to +200 bp), which was not present in HBx cells (Fig. 4a and b). This finding is in concordance with previous work [24]. Conversely, the signals of H3K27Me3 and H3K36Me3 increased in HBx cells compared to normal cells (Fig. 4a, c and d). Interestingly, the signals for these two marks did not change around the TSS, whereas the change in H3K4Me3 was drastic. However, these signals peaked at both sides of the TSS, i.e. around −1200 to −200 bp and +200 to +400 bp.

Both H3K36Me3 and H3K27Me3 were minimally changed around the TSS regions in the livers of normal and HBx mice; however, at the intermediate promoter regions, i.e. –1200 to −200 bp upstream of the TSS, the changes were substantially different between the two conditions (Fig. 4a, c, and d). In addition, the highest peaks of H3K36Me3 and H3K27Me3 changes in HBx were found in intermediate promoter regions, i.e. –1200 to −600 bp from the TSS.

### Pattern 155 is enriched in high CpG content

**Fig. 5:**
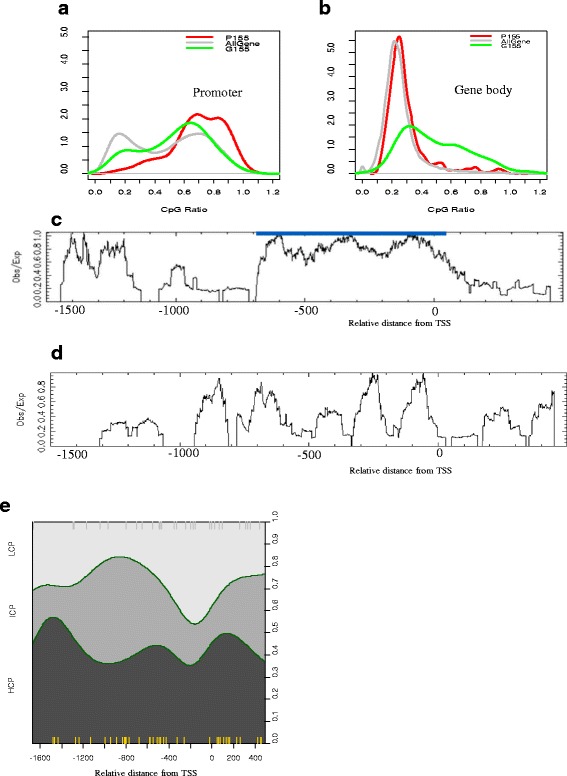
CpG ratio bias in Pattern 155. CpG ratio distributions in promoters and gene bodies for P155 (*red*), G155 (*green*), and all transcripts (*grey*). **a** In promoters, a high peak for the CpG ratio in P155 was observed where the CpG ratio was > 0.6, whereas two peaks were found for all transcripts, one in the low CpG ratio and one in high CpG ratio. **b** The CpG ratio distribution in gene bodies: 57 % of the G155 shows high CpG content (CpG ratio > 0.5). All transcripts and P155 show high peaks in the low CpG ratio (<0.4). (C and D) CpG ratio distributions alongside promoter regions for all transcripts: **c** HCPs (Additional file 3: Figure S4) and (**d**) LCPs of P155. **e** The proportion of 200 base pair intervals matched to Pattern 155 that corresponds to HCPs, ICPs, and LCPs alongside promoter regions. HCPs are more likely to match in intermediate or distal promoter regions, whereas LCPs are likely to match in proximal promoter regions around the TSS

Many previous studies [25–28] have addressed the association between CpG islands (CGIs) and epigenetic and functional regions. In concordance with this previous work, Pattern 155 was characterised with high CpG content (HCG) and strongly associated with high CpG density in promoters and gene bodies (Fig. 5a and b). We found that 67.6 % of the promoter pattern P155 consisted of high CpG content promoters (HCPs), whereas a small fraction (12 %) of P155 contained low CpG content promoters (LCPs). The enrichment of HCPs in P155 was statistically significant in comparison with the promoters in the mouse genome (*P* < 2.2 × e^−16^, chi-square test). HCG was also significant in the gene body pattern G155 (Fig. 5b, *P* = 8.4 × 10^−4^). We plotted CpG ratio distribution along the promoter regions and found strong aggregated peaks (Fig. 5c) ~650 bp upstream of the TSS (−650 to +50 bp). HCPs (Additional file 3: Figure S5) showed an analogous CpG distribution to P155 where high peaks of CpG ratio were densely concentrated on the two specific regions, the proximal (−650 to +50 bp) and distal promoter (−1500 to −1200 bp) regions. Some CpG ratio peaks in LCPs were more likely to be found in proximal regions surrounding the TSS up to 300 bp upstream and in intermediate promoter regions (−900 to −600 bp) (Fig. 5d); however, these peaks were not as high as those of the HCPs, and the peak regions were shallow.

We also investigated the possibility that pattern matched regions in gene bodies (G155) were associated with CGIs and other features of the mouse genome (Additional file 4: Figure S1). CGIs in G155 were preferentially enriched in UTR5 vs. UTR3 (odds = 3.33) and coding exons vs. intronic regions (odds = 1.32) compared with those of the mouse genome, suggesting that the gene body pattern was enriched in highly functional regions of the genome (e.g. UTR5 and coding exonic region) overlapping with high CpG content.

### Changes in histone modifications are associated with CpG distribution

The shape of the CpG ratio distribution along with promoter regions (Fig. 5c) was associated with the changes of each histone modification mark in P155 (Fig. 4a–d). The hypomethylated regions of H3K4Me3 around the TSS (−600 to +400 bp) (Fig. 4A and B) overlapped with the high peaks (−650 to +50 bp) of the CpG ratio distribution (CpG ratio ≥ 0.74; the blue line in Fig. 5c). The peaks of H3K36Me3 and H3K27Me3 appeared in intermediate regions and the distal promoter regions (−1000 to −400 bp), overlapped with low CpG content regions (−1200 to −650 bp).

Variations of each histone modification mark in the pattern were distinctively characterised in response to each promoter class (Fig. 4e for HCPs of P155; Fig. 4f for LCPs). The variations in histone modifications in HCPs resembled those of the P155, whereas those in LCPs were similar to the P155 but with different shapes. Some CpG shore regions (TSS, −200, −500, −1000, and −1400 bp) between HCPs and LCPs showed peaks of H3K27Me3 and H3K36Me3. These observations imply that the variations of histone modifications in HCPs of the pattern rendered the main signature of the pattern, which were weakly preserved in LCPs. These observations are consistent with the results of the correlation network analysis for P155 (Fig. 3b), indicating that CpG content has a positive relationship with H3K4Me3 changes and a negative relationship with H3K27Me3 changes in HBx.

### Comparison with random sampling

P155 comprises both HCPs and LCPs according to their CpG content. We examined whether epigenetic signatures of these groups are different from those observed in the mouse genome. To do so, both HCPs and LCPs in P155 were compared to those of randomly chosen sets. The drastic changes of H3K4Me3 around the TSS regions were conserved in randomly selected top *K* (*K* = 1000, 953 HCPs remained after filtering) HCPs (RHCPs) with high CpG ratios (≥0.88) from the mouse genome (Additional file 3: Figure S3). We observed the loss of H3K4Me3 around the proximal promoter regions with high CpG content in comparison with the change of H3K4Me3 in randomly selected top *K* LCPs (RLCPs), where *K* = 1000 and CpG ratio ≤ 0.4 (Additional file 3: Figure S4). Changes of H3K27Me3 and H3K36Me3 in Pattern 155 were not reproduced in RHCPs (Additional file 3: Figure S3) and RLCPs (Additional file 3: Figure S4). For example, all three histone marks in RHCPs showed relative demethylation alongside the promoter regions, particularly those with high CpG ratios (Additional file 3: Figure S3). However, in P155, H3K27Me3 and H3K36Me3 remained unmethylated or unmodified in the proximal promoter regions and gained in the intermediate promoter regions (Additional file 3: Figure S1 C and D), which were distinguished from RHCPs. RLCPs were devoid of Pattern 155, which was rather weak but conserved in LCPs.

This examination of random sampling suggests that high peak regions of CpG ratio are associated with a loss of H3K4Me3, while regions with low CpG ratio in both promoter classes show high peaks of H3K27Me3 and H3K36Me3.

### Relationships with PolII activity, DNA methylation, and gene expression

**Fig. 6:**
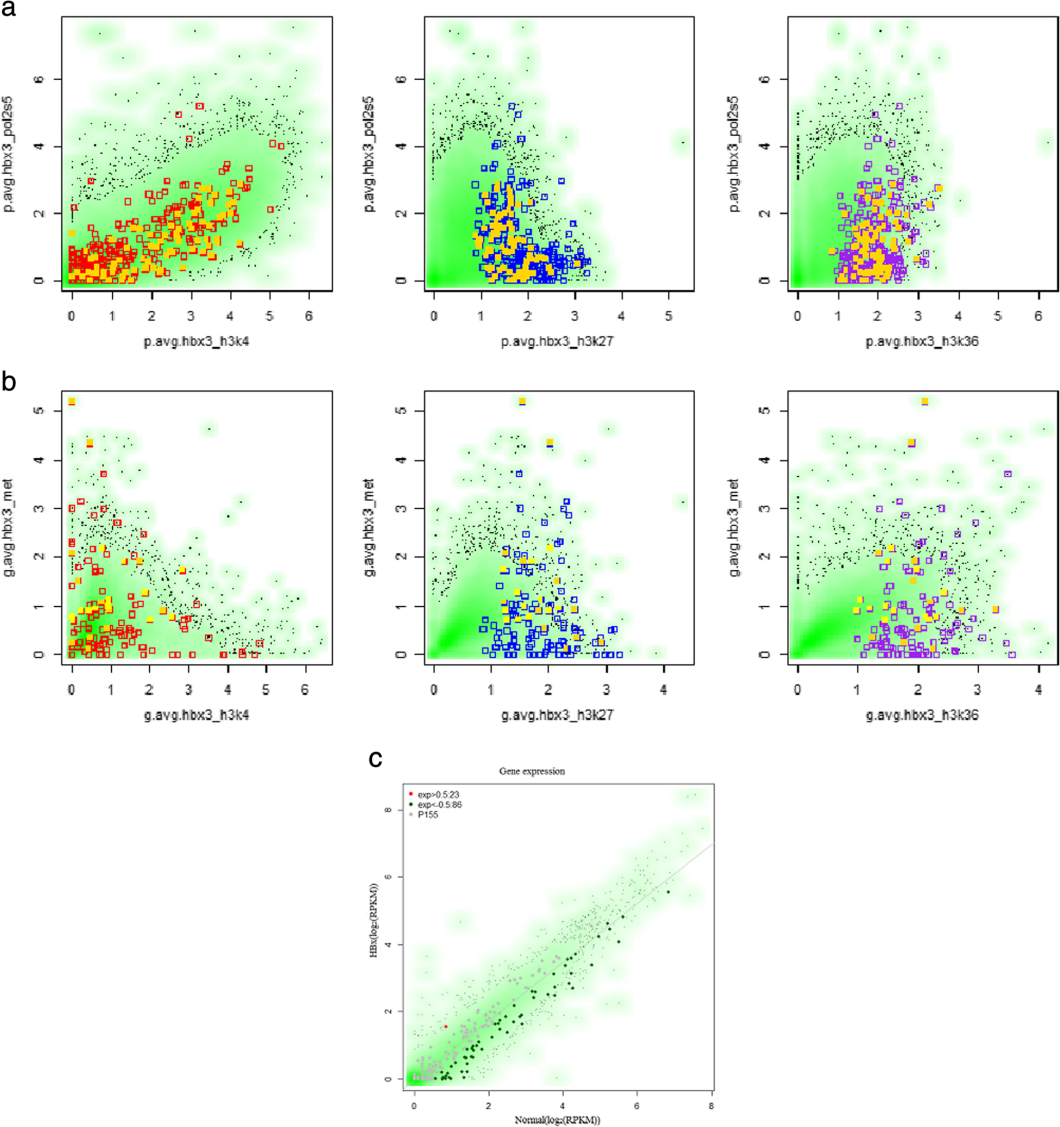
Relationships between histone methylations, PolII, DNA methylation, and gene expression. **a** Relationships between PolIIS5 changes and each of three histone methylation marks in the promoters. **b** Relationships between DNA methylation and histone methylation in gene bodies. In (**a**) and (**b**), a *green* background represents the changes of all transcripts, while red (H3K4Me3), blue (H3K27Me3), and *purple* (H3K36Me3) rectangles represent the changes of (**a**) P155s and (**b**) G155s. *Yellow* rectangles in each plot correspond to the genes for which (**a**) PolIIS5 or (**b**) DNA methylation decreased (dRES < −0.5). **c** Gene expression in normal and HBx TG mouse livers, as measured by RNA-seq and exon array (Additional file 4: Figure S3). The majority of genes in P155 were not differentially expressed (grey dots). Among the differentially expressed genes in P155, substantially more genes were down-regulated (*dark green dots*) than up-regulated (*red dots*)

Overall, RNA polymerase activity (PolIIS5 and PolIIS2) was strongly correlated with each mark of Pattern 155. In both P155 and G155, negative relationships between PolII and H3K27Me3, and between PolII and H3K36Me3, were different from the relationships among all transcripts (Rules 4 and 18 in Table 1, and Fig. 3a–c). This observation is apparent in regions where serine 2 (Additional file 4: Figure S2) or serine 5 (Fig. 6a) phosphorylation of RNA polymerase II decreased by more than a 0.5 differential RES (dRES) between HBx and normal livers (dRES of Pol II ≤ −0.5), particularly in the promoter pattern (Fig. 6a). In P155, the promoter pattern was associated with unchanged states of DNA methylation (Rule 5). However, the gene body pattern was associated with both gain and loss of DNA methylation (Rules 18–23 and Fig. 6b). Exons overlapping with CGI in G155 also tended to be hypomethylated, which is rather strongly observed in more than 0.5 RES hypomethylation (dRES ≤ −0.5 and Fig. 6b). Most of the genes in Pattern 155 were not differentially expressed between normal and HBx-transformed cells. Those that were differentially expressed were down-regulated (9.7 % in the exon array and 30 % in RNA-Seq; Fig. 6c). In RNA-Seq, 86 (78.9 %) out of 109 differential expressed genes were down-regulated. The relationships observed in the network analysis between each mark and gene expression were also observed in down-regulated genes in P155 (Additional file 4: Figure S4).

### Interpretation of functional annotations

According to DAVID functional enrichment analysis and Ingenuity Pathway Analysis (IPA), both the promoter pattern and gene body pattern showed similar functions (Additional file 5: Table S1 and Table S2). Both promoter and gene body patterns were enriched in the ‘transcription regulator activity’, ‘DNA binding’, and ‘transcription factor activity’ functional categories, as indicated by Gene Ontology (GO) and Protein Information Resource (PIR) keyword annotations (Table 3) in DAVID.

**Table 3:** Enriched functional terms and canonical pathways, identified using DAVID and ingenuity pathway analysis (IPA)

Category	Term or pathway	*P*-value
Promoter		
SP_PIR_KEYWORDS	Transcription regulation	2.00E-08
GOTERM_MF	Transcription regulator activity	6.72E-06
GOTERM_BP	Regulation of transcription from RNA polymerase II promoter	8.83E-06
GOTERM_MF	Transcription factor activity	2.56E-05
SP_PIR_KEYWORDS	Phosphoprotein	5.31E-05
SP_PIR_KEYWORDS	DNA-binding	9.99E-05
GOTERM_BP	Regulation of RNA metabolic process	1.31E-04
GOTERM_BP	Positive regulation of transcription	1.43E-04
SP_PIR_KEYWORDS	Developmental protein	1.56E-04
SP_PIR_KEYWORDS	Activator	1.85E-04
SP_PIR_KEYWORDS	Repressor	2.61E-04
Canonical pathway^a^	Role of NFAT in cardiac Hypertrophy	4.36E-06
	Wnt/β-catenin signalling	2.42E-04
	Molecular Mechanisms of Cancer	3.91E-04
	cAMP-mediated signalling	5.60E-04
	Dopamine-DARPP32 Feedback in cAMP signalling	6.05E-04
Gene body		
GOTERM_MF	DNA binding	1.82E-07
INTERPRO	IPR001766:Transcription factor, fork head	3.34E-06
GOTERM_MF	Sequence-specific DNA binding	9.11E-06
SP_PIR_KEYWORDS	Developmental protein	9.66E-06
GOTERM_MF	Transcription regulator activity	4.65E-05
GOTERM_MF	Transcription factor activity	9.25E-05
SP_PIR_KEYWORDS	Transcription regulation	1.62E-04
Canonical pathway^a^	Notch signalling	4.89E-05

Only annotations with *P* < 0.02 after Benjamini-Hochberg correction for multiple hypothesis testing are presented. Full lists and more details are provided in Additional file 5: Table S1 and S2

^a^Canonical pathways were outputs from IPA analysis; other significant functional annotation terms were obtained from DAVID analysis

IPA generated seven significant canonical pathways (including NFAT, Wnt/β-catenin, cAMP mediated, Ras, and PhoGDI signalling) for the promoter pattern and one significant pathway, Notch signalling, for the gene body pattern (*P* < 0.02 after BH correction) (Table 3). Out of the seven significant canonical pathways for the promoter pattern, ‘molecular mechanism of cancer’ implied that genes in promoter patterns could play a role in molecular pathways for cancer. Significant pathways such as Wnt/β-catenin [29–32], Ras [33–35] and cAMP mediated [36–38] are already known to be involved in the pathogenesis of human HCC development [39–42]. The role of NFAT (Nuclear Factor of Activated T cells) in cardiac hypertrophy [43] in our IPA analysis can be explained by a similar role as a factor in HBx related HCC through previous work [44–46]. For instance, it was reported that HBx activates transcription and nuclear translocation of NFAT regulating cytokine encoding genes such as TNF-α whose production was observed in chronic liver injury and inflammation leading to development of HCC [44, 45]. The Notch signalling pathway, which was significantly enriched in the gene body pattern, mediates tumour invasion in HCC, which suggests that inhibition of Notch signalling pathway inhibitors could suppress invasion of HCC cells via the extra cellular signal-regulated kinases 1 and 2 (ERK1/2) signalling pathways [47].

Functional annotations for differential expressed genes (>2 fold changes) using DAVID were shown in Additional file5: Table S3. In terms of biological process and functions, they are involved in regulation of cell death and metabolism of xenotiotics by cytochrome P450 while genes in Pattern 155 are enriched in regulation of transcription and transcription factor activity.

Taken together, previous studies largely verified the pivotal roles played by the eight significant pathways we identified using IPA and our findings were consistent with the current knowledge regarding HBx-induced HCC.

## Discussion

In this study, we developed *ChARM*, an unsupervised approach that uses ARM, a well-known method for finding frequent patterns in large databases, for the discovery and interpretation of *de novo* combinatorial epigenetic modification patterns that occur globally in a cancer cell line. We applied *ChARM* to investigate an HBx-transformed mouse liver tumour model and discovered an aberrant histone modification pattern (a combination of a loss of H3K4Me3 and gains of H3K27Me3 and H3K36Me3). The pattern characterised with CpG content of underlying DNA sequences-H3K27Me3 and H3K36Me3 hypermethylation in HBx occurred in intermediate promoter regions where CpG ratio is low. There is a possibility those signals reflected by neighbouring genes intersecting with the promoter regions of the pattern. In some cases, the gains of H3K36Me3 were observed in exonic regions of neighbouring upstream genes or overlapping ESTs, implying that exonic enhancers function in promoter regions. As we found in correlation network analysis, a positive relationship between H3K4Me3 and CpG content and negative relationships between H3K27Me3 and H3K36Me3 and CpG content were conserved in the patterns, whereas these relationships were not replicated in the whole mouse genome and random samples. The pattern observed in this study was enriched in functional elements such as UTR5, coding exons, and promoters, in response to CpG content. The pattern was associated with Pol2 activity and gene expression, where a small portion of genes in the promoter pattern showed mostly down-regulated expression. Interestingly, while the majority of the genes in the promoter pattern showed no significant changes in expression levels, the pivotal roles played by some of these genes (e.g. PTEN) in HCC progression has already been highlighted in previous studies [40], suggesting that these specific genes in the pattern could be potential predictors of epigenetic prognosis in HBx. The significant canonical pathways enriched in the pattern accounted for the pathogenesis of HBx; for example, Notch signalling, and Wnt/β-catenin, cAMP mediated, and Ras pathways, are linked to a general cancer pathway. Our results indicate that histone modifications in the promoter pattern could regulate mis-expression of the downstream genes. The observation that most genes were down-regulated suggests that the genes in the pattern may play a role in inhibition of oncogenic pathways in HBx and, therefore, they could be candidates for further investigation of the epigenetic mechanisms in HBx.

From a methodological perspective, the features of *ChARM* are comparable to those of existing computational methods [14, 15, 17, 19, 20]. For epigenetic therapeutic targets, there has been more emphasis on identifying global patterns of combinatorial chromatin signatures. In this context, ARM is able to extract all the possible combinations from 1 to *K*-th large itemsets, which are composed of *K* constituent modifications that meet minimum support and lift from a large chromatin modification data. However, the three existing methods (ChAT, CosBI and ChromaSig) work well for identifying locally aligned similar signatures of different modifications whilst *ChARM* can identify combinatorial patterns composed of distally related peaks of different modifications (Additional file 6: Figure 1S and Figure S2A). Because degrees of confidence, support, and lift are provided in the rules of the method, biologists could determine differential patterns between different cell types more easily than with some existing methods such as ChromHMM based on HMM, Segway based on dynamic Bayesian networks, ChAT based on dynamic programming, and hierarchical clustering and ChromaSig that use probability profiles. Most of previously developed methods do not detect differential modification patterns in a pattern discovery process (Additional file 6: Figure S1-S6).

We evaluated whether the pattern explained variation in gene expression and functions. Some existing methods require a prior knowledge, e.g. the use of motif seeds to initialise the subsequence of the pattern (ChromaSig), local prior knowledge for initial state definition of emission and transitions probabilities (ChromHMM), and corresponding genome annotation for regions around, for example, the TSS, exons, UTR5 and UTR3, and GC rich regions.

The patterns we discovered have flexibility in the representation of chromatin signatures. The pattern is capable of identifying differential combination patterns and can include multiple modes [20] of the constituent modifications. For example, our method can be used to identify the co-localised epigenetic modifications for which differential changes are likely conserved in specific functional elements in different genomic space. The equal frequency binning strategy used in *ChARM* to transform continuous values into categorical bins also gives flexibility and simplicity to find the pattern reflecting an epigenetic modification distribution of each mark.

While *ChARM* has several strengths, it also has some limitations. For example, it does not distinctively distinguish the different shapes of patterns that are composed of the same constituent modifications. However, it provides informative relationships among the constituent modifications, which may not correspond to physical biochemical interactions but are more likely to imply cross-talk between different epigenetic modifications. Thus, our approach is scalable in respect of deriving functionally associated patterns by incorporating epigenetic modifications with other genomic features (e.g. SNP density, conservation, microsatellite, and functions) in a learning model. In future studies, we could take advantage of this, and of *ChARM*’s other qualities, to infer functionally important epigenetic modification patterns.

## Conclusions

We developed *ChARM*, an unsupervised approach that uses ARM, a well-known method for finding frequent patterns in large databases, for the discovery and interpretation of *de novo* combinatorial epigenetic modification patterns that occur globally in a cancer cell line. Consequently, *ChARM* identified combinatorial chromatin patterns of differentially modified regions in an unbiased fashion without using any functional annotations (except gene boundaries). Additionally, it was able to characterise the functional elements and genome features that are enriched in the patterns.

The patterns are expressed as association rules, which are quantitative, informative, and easily interpreted. Biologists could determine interesting rules or differential patterns between different cell types more easily than with some existing methods.

## Methods

### ChIP-seq and gene expression processing

Transgenic mice expressing HBx protein, and the HCC tissues in these mice, have been described previously [48]. Genome-wide DNA methylation [49], histone methylations (H3K4Me3, H3K27Me3, and H3K36Me3), and serine 2 and 5 phosphorylation of RNA polymerase II were profiled from the livers of 3-month old wild-type and HBx transgenic (TG) mice. The gene expression data were downloaded from the Gene Expression Omnibus (GEO accession number: GSE48052 for RNA-Seq [49]). The reads from mRNA-seq were aligned to MM9 (mouse genome build 37) using bowtie2 (version 2.1.0), extended toward the 3′ end for fragments to reach the final 200 bp interval BED format and counted overlapping sequence tags at 50 bp resolution. The RPKM values of RefSeq transcripts were calculated using TopHat/Cufflinks and were log_2_ transformed. The ChIP DNA fragments were sequenced using Solexa sequencing technology and the ChIP-seq reads were mapped to the MM9 mouse reference genome using Bowtie2. We extended the 34-bp reads toward the 3′ according to the average size of library fragments (i.e. 200 bp). The number of overlapping sequence reads mapped to each promoter or gene body was counted and divided by the length of the each promoter and gene body which was normalized by the ratio of the total read count to the genome size ((target read count/target size)/(total read count/genome size)) [48]. This metric measures the relative enrichment of reads within a given genomic locus relative to the whole genome. The relative enrichment score (RES) of the Chip-seq signals for a given genomic locus was obtained by using a log2 ratio, as previously described [50–54]. For each genomic locus, the differential RES (dRES) between HBx and normal livers was calculated by subtracting two RESs between HBx and normal livers.

All the genomic positions of transcripts and CGIs were obtained from the UCSC genome browser and are based on MM9. The NCBI mRNA reference sequences collection (RefSeq) was employed for defining transcription units such as gene bodies and TSSs. We divided each transcript into two large bins, i.e. promoters and gene bodies. Promoter regions were defined as existing in the region 1500 bp upstream to 500 bp downstream from the TSSs of the RefSeq genes, and gene bodies encompassed the boundary of the RefSeq genes.

For each promoter and gene body, we calculated the average RES of each chromatin feature across all transcripts. Each ChIP-seq experimental data set across all promoters or gene bodies was represented with a matrix, which comprised 20,147 coding mRNA × 7 chromatin modification features for all promoters or gene body regions across all transcripts of the RefSeq genes.

### ARM

ARM [55] was originally designed to identify products that were purchased together in customers’ shopping baskets. It identifies frequent patterns of co-occurrence and relationships involving dependence in large data sets containing many items. These patterns are expressed as association rules that describe the dependence or associations among a set of singlet products or items. We have previously shown that association rules as patterns detected by ARM are informative, quantitative, and biologically interpretable [52, 56]. Finding global combinatorial histone modifications can be considered as discovering the *K*-th most frequent itemset: the combination of *K* different epigenetic modification states whose frequency (support) is greater than all the possible combinations composed of *K* modified states, where *K* ≤ N, *K* is the number of different modified states, and N is total number of epigenetic modified states given. The *K*-th most frequent itemset should be subject to the close itemsets but not mandatory to be maximal frequent itemsets. We analysed the frequency of all *K*-large itemsets to find the *K*-th most frequent itemset (Additional file 2: Figures S1 and S2). Association rules were generated from *K*-large itemsets that met minimum confidence.

Let *I* be a set of items and *D* be a set of database transactions, e.g. each set of promoters or gene bodies. Each transaction *T* is a set of items such that *T ⊆ I*. An association rule has the form R: X → Y [c, s], where X (the left-hand side (LHS)) and Y (the right-hand side (RHS)) are the body and the head of a rule, respectively. X and Y are disjoint predicates (X ∩ Y = Ø). Each X and Y consists of a conjunction of distinct predicates that describe items. The strength of the association rules can be measured in terms of their support (s) and confidence (c). The support of a rule (X → Y) is the probability that a case in a database contains both X and Y. The confidence of a rule is the probability that a case contains Y given that it contains X. Thus, the rule indicates strong or partial correlation or dependence between items X and Y encoded in the rule.

$$ \begin{array}{l}\mathrm{Support}\left(\mathrm{X}\to \mathrm{Y}\right)=\mathrm{P}\left(\mathrm{X}\cup \mathrm{Y}\right)\hfill \\ {}\mathrm{Confidence}\left(\mathrm{X}\to \mathrm{Y}\right)=\mathrm{P}\left(\mathrm{Y}\Big|\mathrm{X}\right)\hfill \end{array} $$

For instance, consider an example from our epigenetic data, which can be used to illustrate the concepts described above. In our data, *I =* {p.H3K4Me3 = 1, p.H3K4Me3 = 2, p.H3K4Me3 = 3, p.H3K4Me3 = 4, p.H3K4Me3 = 5, p.H3K27Me3 = 1, p.H3K27Me3 = 2, p.H3K27Me3 = 3, p.H3K27Me3 = 4, p.H3K27Me3 = 5,…, p.H3K36Me3 = 4, p.H3K36Me3 = 5}, *D =* 20,147 transactions of promoters, and a transaction *T* can be formulated with the form of, for example, *T*_*i*_ = {p.H3K4Me3 = 1, p.H3K27Me3 = 5, p.H3K36Me3 = 5}, where *T*_*i*_ ⊆ *I*. The patterns are expressed with association rules, e.g. Rule 1 (Table 1), which is formulated with {p.H3K27Me3 = 5 p.H3K36Me3 = 5} == > {p.H3K4Me3 = 1}. In Rule 1, the support of 1.8 % denotes that there are 362 promoters that show the combination of three histone modification states {p.H3K27Me3 = 5, p.H3K36Me3 = 5, p.H3K4Me3 = 1}, with gains of H3K27Me3 and H3K36Me3 and a loss of H3K4Me3. Confidence of 35 % indicates that 35 % of the promoters that have high gains of H3K27Me3 and H3K36Me show a high loss of H3K4Me3.

### A workflow for the *de novo* pattern discovery of chromatin modifications using ARM

The discovery of *de novo* global CCMPs, given a chromatin modification matrix for a set of promoters or a set of gene bodies, was used to identify frequent combinatorial patterns, which is a typical application of ARM. The pattern discovery procedure included adaption of ARM and interpretation of the patterns discovered. The procedure comprised the following steps: pre-processing and discretization, ARM rule generation, clustering of association rules for visualization, refinement of the patterns (Fig. 1), and interpretation of the patterns.

### Pre-processing and discretization

For each of the promoters or gene bodies, we calculated the differential RES for the HBx and normal cells of each chromatin modification in order to identify variation in the patterns of modification between the two conditions. ARM is not directly applicable to continuous types such as our ChIP-seq experimental data; therefore, we used discretization to transform the continuous data into categorical data based on an equal frequency discretization algorithm. In this process, the continuous data for each ChIP-seq mark were divided into five bins (*b* = 1, …, *n*; *n* = 5): extremely hypo-changed (*b* = 1), hypo-changed (*b* = 2), unchanged (*b* = 3), hyper-changed (*b* = 4), and extremely hyper-changed (*b* = 5). Because significant ChIP-seq peaks were skewed, the adoption of equal width discretization might ignore a small number of outliers.

Although our major aim was to identify global changes to epigenetic modifications, these are likely to represent a relatively small portion of the genome. Therefore, equal frequency, rather than equal width, discretization is more appropriate for minimising the loss of outliers, which represent extreme changes, and prioritising the discovery of relatively weak patterns.

### Association rule generation

The generation of association rules was carried out by using the APRIORI algorithm [55]. We used Oracle Data Miner for discretization and the *arules* Package in R, which implemented the APRIORI algorithm, for ARM. We ran ARM over five states for each of the six epigenetic modification marks in 20,147 mRNA transcripts and their corresponding promoters. An item corresponded to a modified state of each mark, and a collection of these items in each gene body or promoter of a transcript corresponded to a transaction. We set a minimum support and a minimum confidence of 0.05 and 30 %, respectively.

We focused on detecting relatively weak and rare but epigenetically meaningful patterns against strong patterns, which occur frequently, have high support, and represent well-known common correlations. The majority of strong patterns with high support and high confidence will characterise the combinations in unchanged states. To prioritize a small portion of the modification states in the whole genome, and to ensure that infrequent itemset generation was not missed, we set low thresholds for support (e.g. 0.05 %) and confidence (e.g. 30 %), generated as many rules as possible, and filtered them by measures of interestingness e.g. Lift. The existing association rule mining formulation relies on the support and confidence measures to eliminate uninteresting patterns. The drawback of support was that many potentially interesting patterns that might have weak pairwise co-occurrences but have strong multi-item co-occurrences might be eliminated by the support threshold due to their low supports.

In the discovery of combinatorial patterns, the confidence metric can mislead and reveal directional information. Therefore, a metric known as lift [57] is more suitable for adopting measures of interestingness and the co-occurrence of epigenetic modification states in the different marks in the patterns. Lift is defined as follows:

$$ Lift=P\left(X,Y\right)/P(X)\times P(Y) $$

Lift calculates the ratio between the rule’s confidence and support of item *Y* in the rule’s consequence. It was originally known as interest, and measures how many times more often X and Y occur together than expected if they are statistically independent. If the result of improvement is <1, >1, or equal to 1, then the relationship of X and Y is negatively correlated, positively correlated, or independent, respectively. In this study, lift allowed us to measure the possibility of interplay between epigenetic modification states in the pattern and provided us with baseline information for determining whether the pattern implied cross-talk between histone modifications. We selected rules representing the combination of two or more epigenetic modification states that appeared in the pattern and calculated the lift (Table 1).

### Clustering association rules for visualisation of the patterns

ARM results in a large number of discovered rules; thus, identifying and globally visualising rules of interest are not easy tasks for analysts. Therefore, we present a new approach to post-processing and visualisation of rules that makes interpretation more feasible. In our approach, by parsing epigenetic modification states in all the rules discovered, we generated a rule matrix in which each row represents a rule and each column stands for each epigenetic mark. The cells of each row in the rule matrix were filled with the chromatin modification states of each mark represented as the intensity of gene expression. In order to visualise association rules by epigenetic marks using TreeView and Gene Cluster 3.0, which are broadly employed in analysis of gene expression data, we transformed the scale of the bins representing the modification states (e.g. 1, 2, 3, 4, 5) of each epigenetic mark into a range of values (e.g. -2, −1, 0, 1, 2). After clustering the rule matrix, we used Gene Cluster 3.0 to graphically capture the global view of the discovered rules, and we visualised the clustering results with TreeView (Fig. 2a). For both promoters and gene bodies, the global view of association rules clustered by their items, i.e. chromatin modification states (Fig. 2a), represents combinatorial patterns of epigenetic modifications. Each association rule (i.e. each row in Fig. 2a) encodes each combination of differential modification states for given chromatin marks.

### Identification of functional elements enriched in the pattern

We refined the pattern discovered in order to filter out false positives and to obtain fine-grained targets. We searched 200-bp intervals, for which epigenetic modification states were congruent to the gene-level patterns, throughout the mouse genome. To investigate genomic features associating with the patterns, we identified the functional elements that were enriched in these 200-bp intervals. Moreover, we calculated the frequency of the functional elements (e.g. coding exons, introns, UTR5, UTR3, and promoters) in the patterns and the genome. Odds for a functional element *f* in the patterns, with respect to values expected from the relative size of the mouse genome, were calculated as follows:

1 $$ \mathrm{Odds}\kern0.5em \mathrm{f}\mathrm{o}\mathrm{r}\kern0.5em \mathrm{f}\mathrm{unctional}\kern0.5em \mathrm{element}\kern0.5em f=\left[\frac{p_a{q}_m}{p_{{}_m}{q}_a}\right] $$

*p*_a_, *p*_m_: probabilities of functional element *f* appeared in each of the patterns and the mouse genome.

*q*_a_, *q*_m_: (1- *p*_a_) and (1- *p*_m_) respectively.

Odds ratios for two functional elements *f*_*i*_ and *f*_*j*_ are calculated by odds (*f*_*i*_)/odds (*f*_*j*_) as shown in Table 2.

### Correlation network analysis for identifying relationships between chromatin marks

To investigate the relationships and dependency between the epigenetic modifications across a given set of genes, we generated a correlation matrix based on all columns (epigenetic modifications). We took the upper diagonal of the correlation matrix as an input to calculate an adjacency matrix of a graph in order to draw a correlation network. To transform the correlation matrix into the adjacency matrix of the graph, the cells in which Pearson’s correlation (r) was < 0.2 were set to zero and other cells were taken. The columns of the adjacency matrix represented epigenetic modifications as nodes, and the non-diagonal cells of the adjacency matrix represented the correlation (r) between the nodes as assigned to edges. We visualised the adjacency matrix of the graph by using *igraph* R module 2.12 to produce a correlation network (Fig. 3 for promoters). Correlation values were assigned to the width of edges and vertex size was proportional to degrees of the vertices.

### Characterisation and interpretation of the patterns

Our method characterises patterns by analysing the associations between the following genomic features: CpG content, propensity of spatial positions along the promoter regions, and relationships with gene expression. We further investigated the relevance of these genomic features to histone modification changes by dividing each promoter into three regions relative to TSS according to the work of Koga et al. [28]: proximal (−200 to +500 bp), intermediate (−200 to −1000 bp), and distal (−1000 to −1500). We also classified promoters into three groups based on their CpG ratio [28]: low CpG (LCPs), intermediate CpG (ICPs), and high CpG (HCPs) content promoters.

We used Benjamini-Hochberg corrected Fisher exact tests in IPA software (Ingenuity Systems, http://www.ingenuity.com) to analyse the association of the genes identified in the promoter and gene body patterns with molecular functions, cellular functions, and canonical pathways. Similarly, functional enrichment analyses were performed using DAVID (Database for Annotation, Visualisation and Integrated Discovery, http://david.abcc.ncifcrf.gov). From the DAVID analysis, we reported GO terms related to the Biological Process and Molecular Function ontologies, KEGG pathways, and terms from InterPro and PIR at Level 3.

## Additional files

Additional file 1: Association rules. A set of all association rules discovered for promoters and gene bodies present and rules are sorted by lift and support. **Table S1.** All association rules discovered in promoters. **Table S2.** All association rules discovered in gene bodies. **Table S3.** mRNA IDs in P155. **Table S4.** mRNA IDs in G155. **Table S5.** Pattern Specification. **Table S6.** Supersets of G155. **Table S7.** Filtered rules for P155. **Table S8.** Filtered rules for G155. (XLSX 310 kb)
Additional file 2: Frequency plots for *K*-itemsets (*K* = 3). **Figure S1.** The frequency plot of *K*-itemsets for gene bodies. **Figure S2.** The frequency plot of *K*-itemsets for promoters. **Figure S3.** Plots of lift and rule length. **Figure S4.** Correlation network analysis for gene bodies. **Figure S5.** Clustering normalized ChIP-seq read count for patterns. **Figure S6.** Clustering normalized top K rules (*K* = 87). **Figure S7.** Distributions of three Histone Methylation in Pattern 155. (PPTX 1177 kb)
Additional file 3: Epigenetic profiles of the patterns. **Figure S1.** Epigenetic modification changes of HCPs in P155. **Figure S2.** Epigenetic modification changes of LCPS in P155. **Figure S3.** Epigenetic modification changes of random HCPs (*n* = 1000). **Figure S4.** Epigenetic modification changes of random LCPs (*n* = 1000). **Figure S5.** CpG distributions of HCPs and LCPs in P155 and pattern matching region. **Figure S6.** CpG distributions of groups composing of chromatin mark changes. (PPTX 772 kb)
Additional file 4: Characterisation and interpretation of the patterns. **Figure S1** Enrichment of CGI in the pattern. **Figure S2.** Pol2SII changes in the gene body pattern. **Figure S3.** DNA methylation changes in the promoter pattern. **Figure S4.** Differential gene expression. **Figure S5.** Gene expression vs. histone modification marks in the pattern. **Figure S6.** Epigenetic changes of each transcript in the promoter pattern. **Figure S7.** Epigenetic changes of each transcript in the gene body pattern. (PPTX 1164 kb)
Additional file 5: Functional annotation for the pattern. **Table S1.** Functional enrichment analysis for the promoter pattern. **Table S2.** Functional enrichment analysis for the gene body pattern. **Table S3.** Functional enrichment analysis for differentially expressed genes. (XLS 168 kb)
Additional file 6: Comparison with ChAT. **Figure S1.** Pattern detected by ChAT on a ChIP-seq data set from livers of HBx TG mice. **Figure S2.** Multi-mode signatures composing of the same histone modifications. **Figure S3.** Pattern detected by ChAT on a ChIP-seq data set from livers of normal mice. **Figure S4.** Comparison of HBx with Normal: H3K4me3. **Figure S5:** Comparison of HBx with Normal: H3K36me3. **Figure S6.** Comparison of HBx with Normal: H3K27me3. (PPTX 909 kb)
