# Supplementary material for: ChARM: Discovery of combinatorial chromatin modification patterns in hepatitis B virus X-transformed mouse liver cancer using association rule mining
Source: BMC Bioinformatics. 2016 Dec 13;17(Suppl 16):452. doi: 10.1186/s12859-016-1307-z (PMC5249029; doi:10.1186/s12859-016-1307-z)
Supplement: Additional file 2: — Frequency plots for K-itemsets (K = 3). Figure S1. The frequency plot of K-itemsets for gene bodies. Figure S2. The frequency plot of K-itemsets for promoters. Figure S3. Plots of lift and rule length. Figure S4. Correlation network analysis for gene bodies. Figure S5. Clustering normalized ChIP-seq read count for patterns. Figure S6. Clustering normalized top K rules (K = 87). Figure S7. Distributions of three Histone Methylation in Pattern 155. (PPTX 1177 kb) [file 12859_2016_1307_MOESM2_ESM.pptx]

## Slide 1
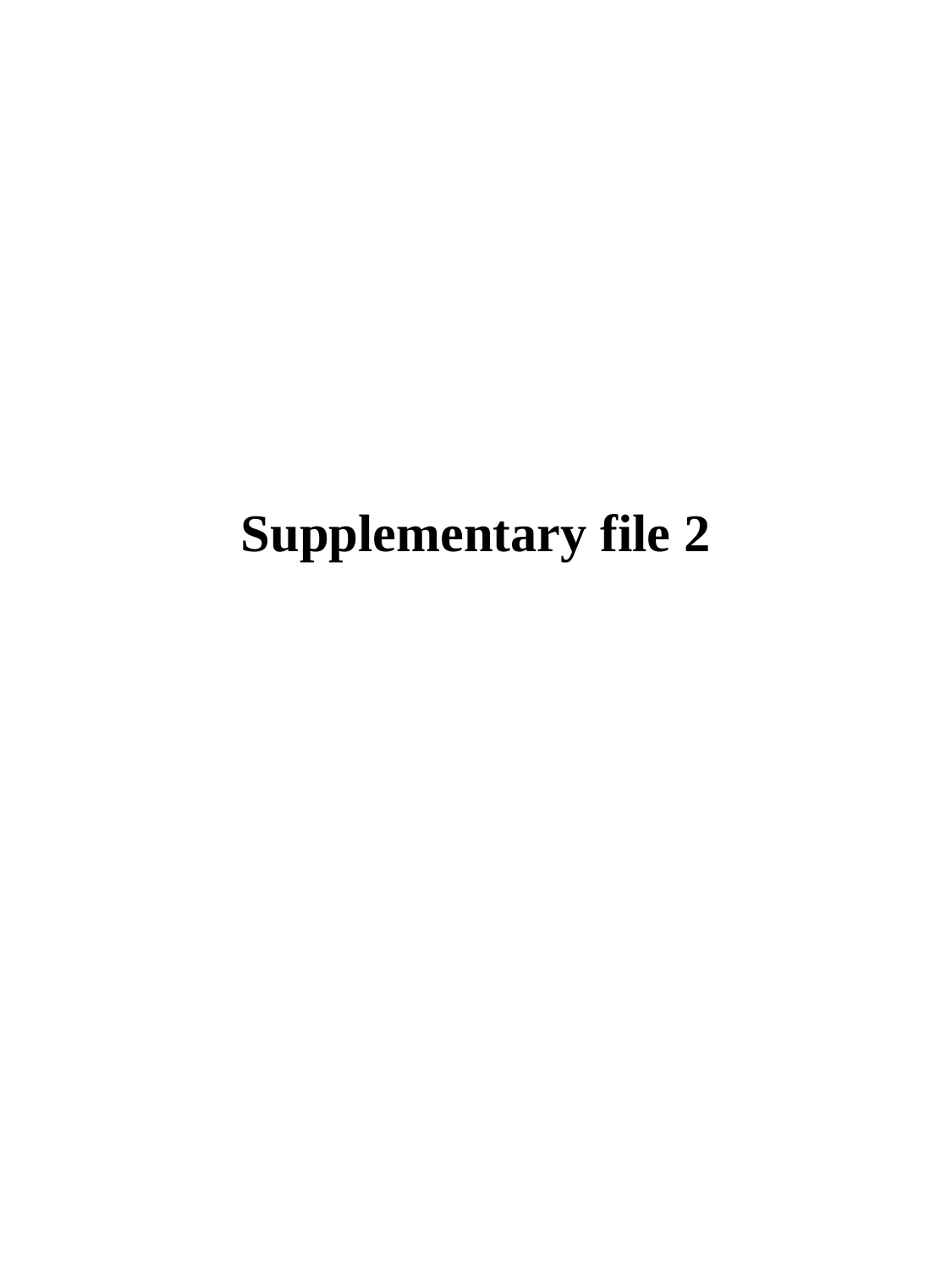

# Supplementary file 2

## Slide 2
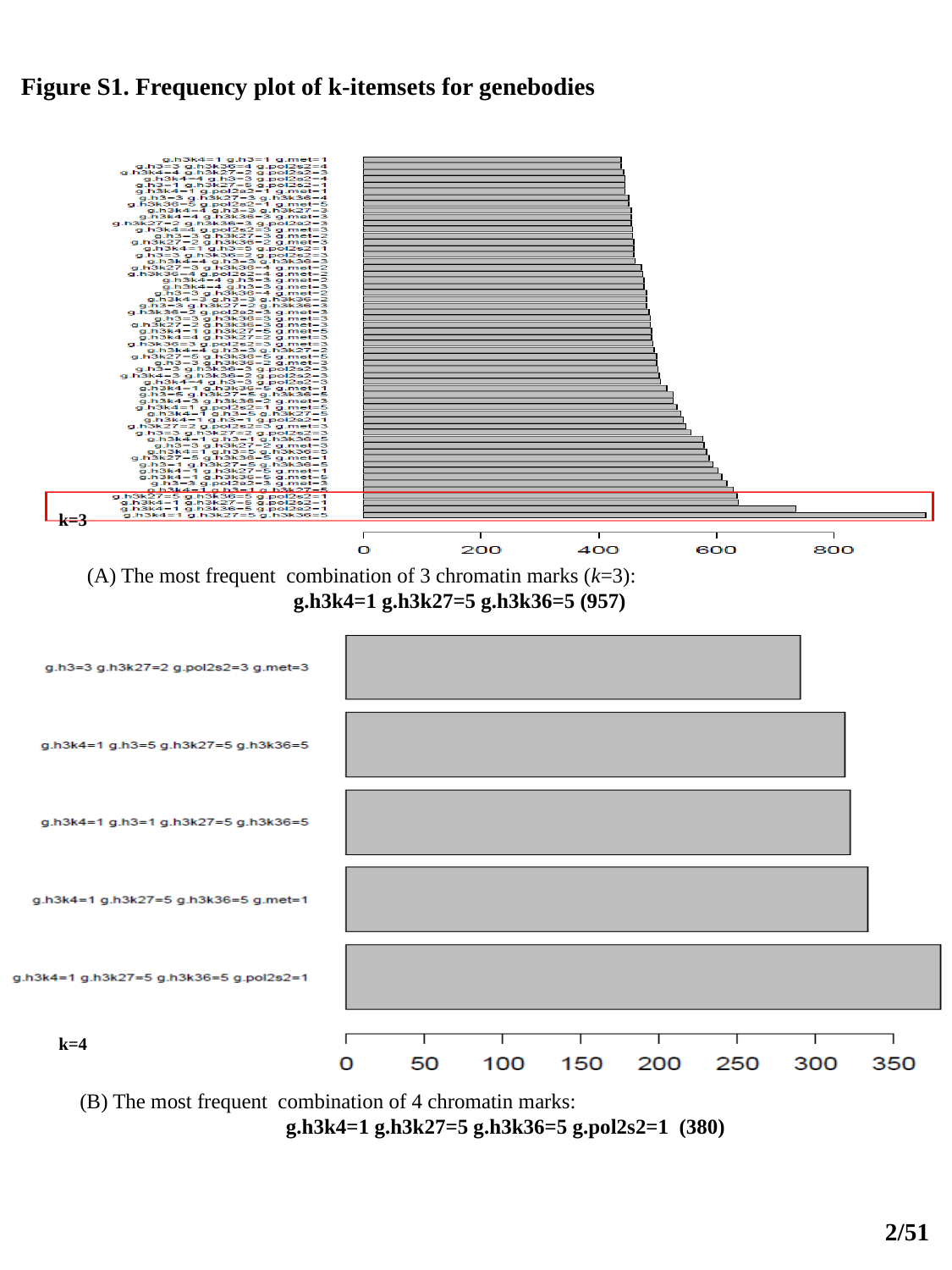

# Figure S1. Frequency plot of k-itemsets for genebodies
k=3
(A) The most frequent combination of 3 chromatin marks (k=3):
g.h3k4=1 g.h3k27=5 g.h3k36=5 (957)
k=4
(B) The most frequent combination of 4 chromatin marks:
g.h3k4=1 g.h3k27=5 g.h3k36=5 g.pol2s2=1 (380)
2/51

## Slide 3
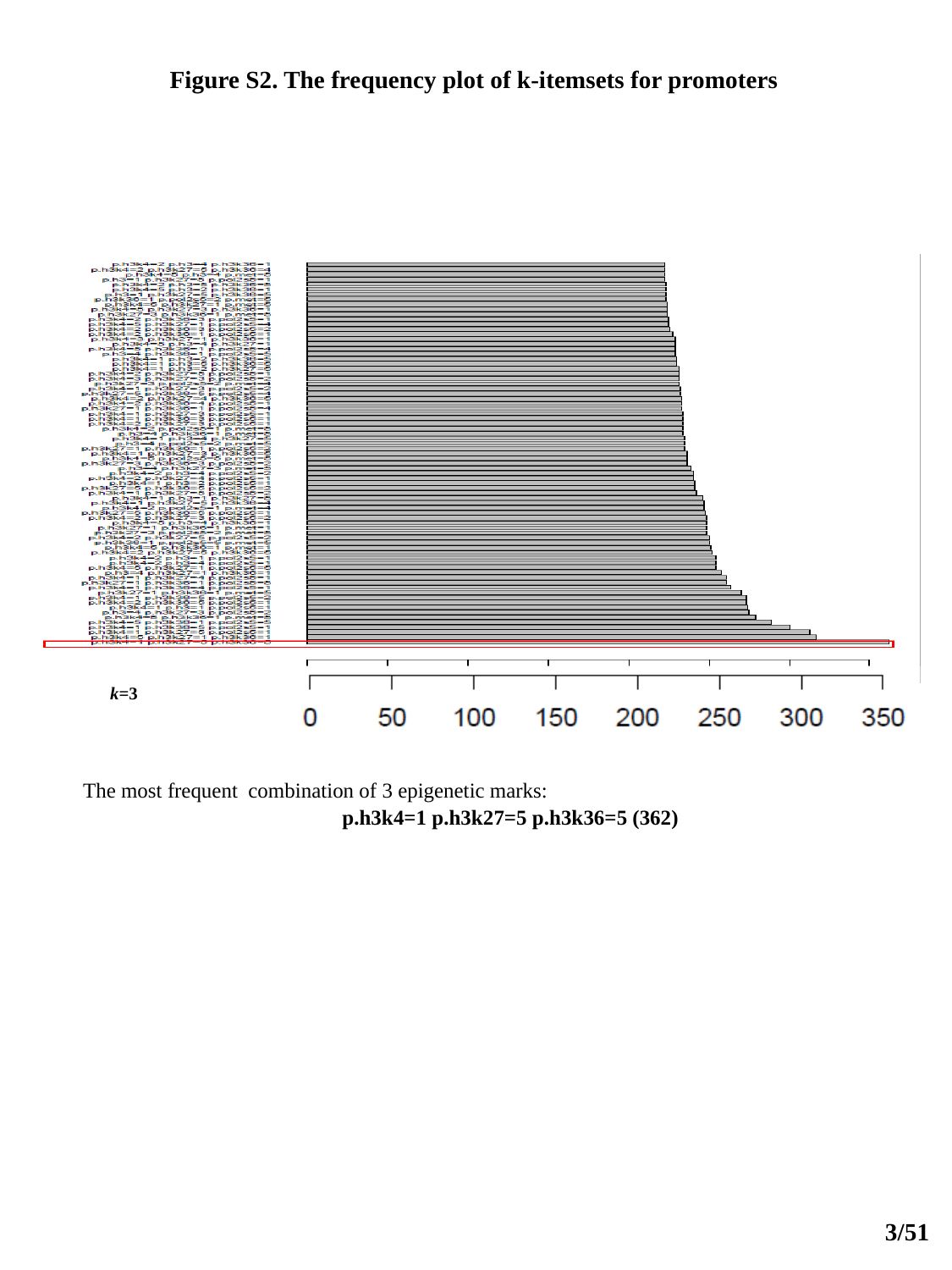

# Figure S2. The frequency plot of k-itemsets for promoters
k=3
The most frequent combination of 3 epigenetic marks:
		 p.h3k4=1 p.h3k27=5 p.h3k36=5 (362)
3/51

## Slide 4
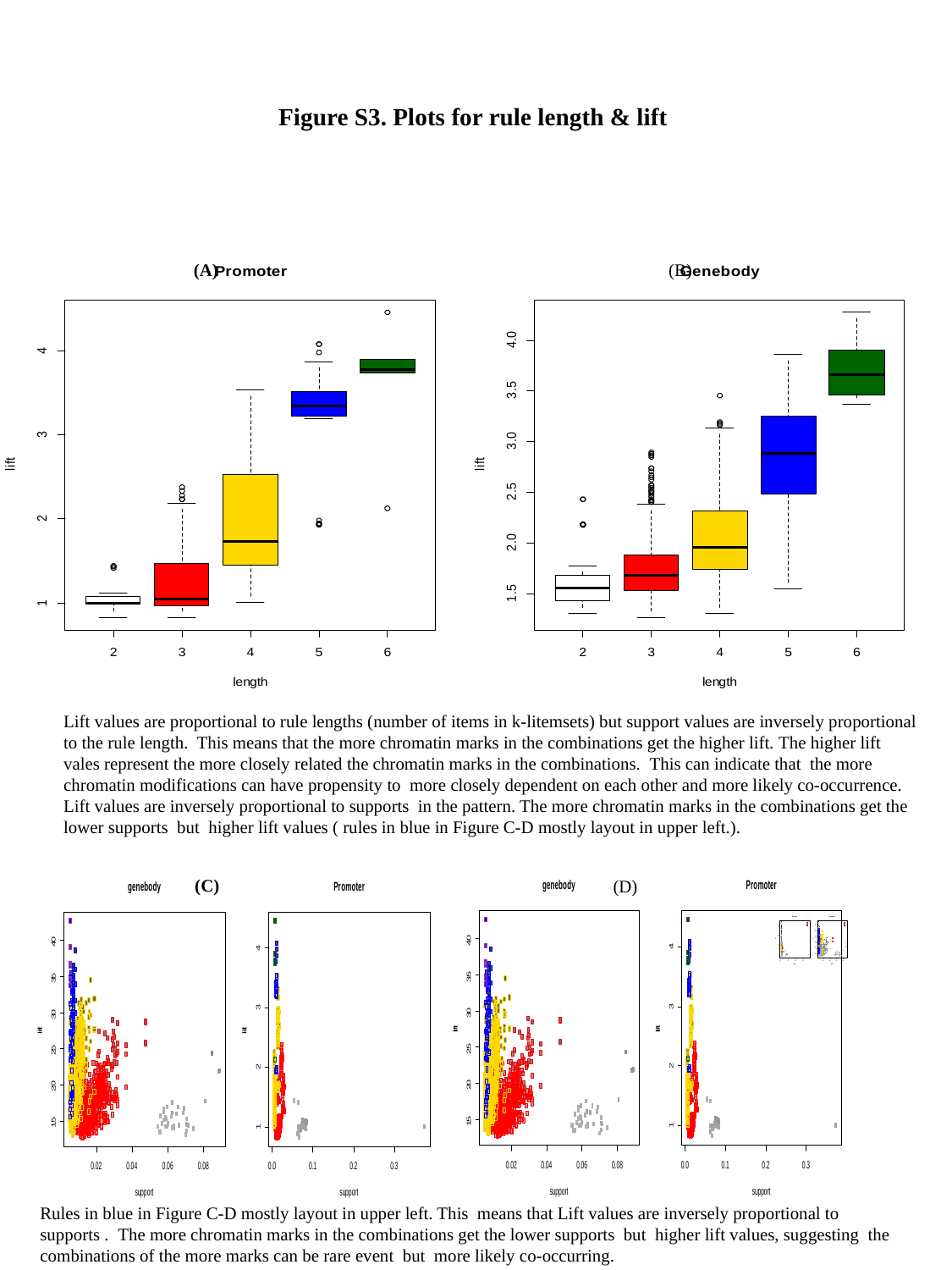

# Figure S3. Plots for rule length & lift
(A)
(B)
Lift values are proportional to rule lengths (number of items in k-litemsets) but support values are inversely proportional to the rule length. This means that the more chromatin marks in the combinations get the higher lift. The higher lift vales represent the more closely related the chromatin marks in the combinations. This can indicate that the more chromatin modifications can have propensity to more closely dependent on each other and more likely co-occurrence.
Lift values are inversely proportional to supports in the pattern. The more chromatin marks in the combinations get the lower supports but higher lift values ( rules in blue in Figure C-D mostly layout in upper left.).
(C)
(D)
Rules in blue in Figure C-D mostly layout in upper left. This means that Lift values are inversely proportional to supports . The more chromatin marks in the combinations get the lower supports but higher lift values, suggesting the combinations of the more marks can be rare event but more likely co-occurring.

## Slide 5
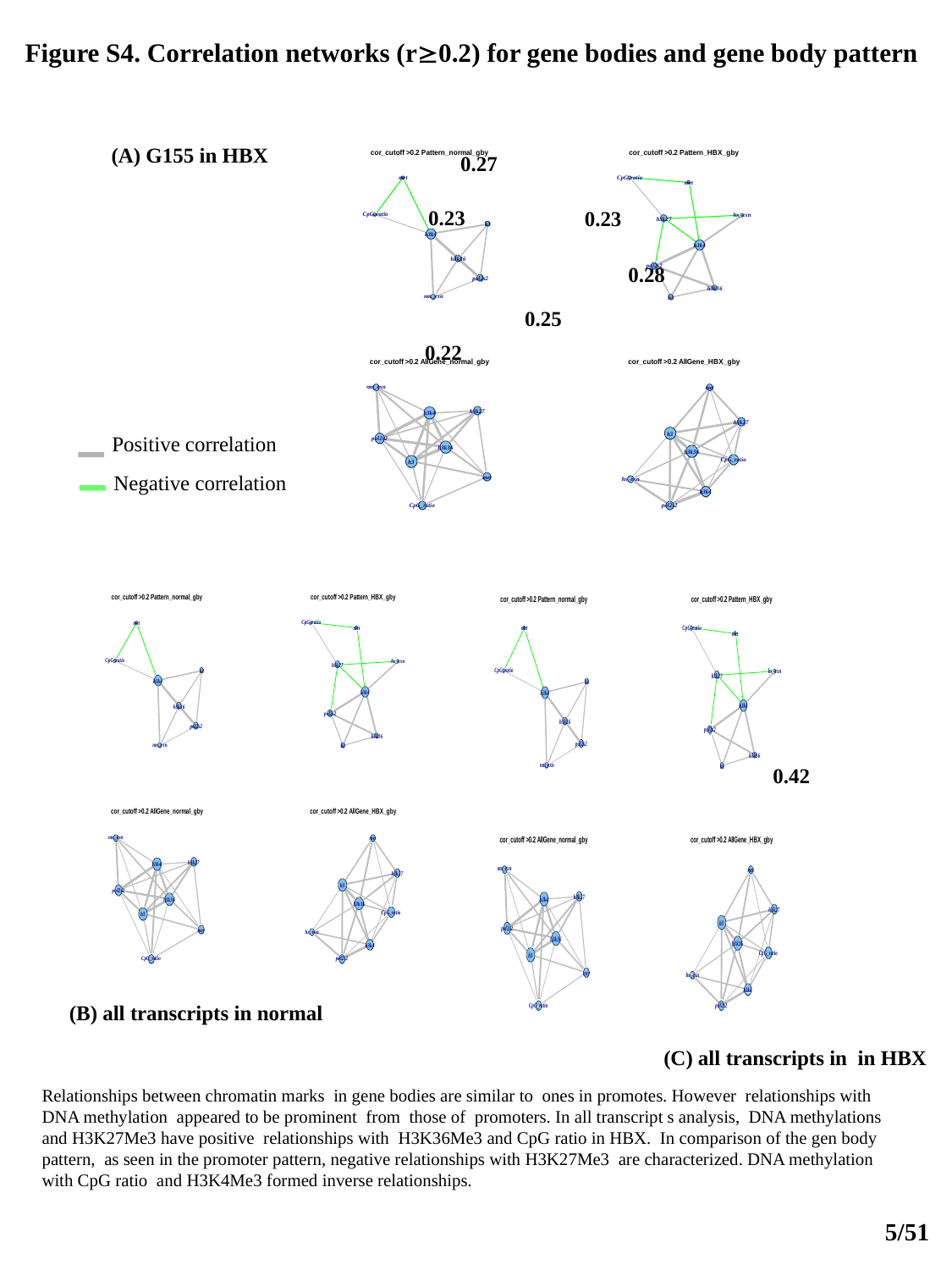

# Figure S4. Correlation networks (r≥0.2) for gene bodies and gene body pattern
(A) G155 in HBX
0.27
0.23
0.23
0.28
0.25
0.22
Positive correlation
Negative correlation
(B) all transcripts in normal
0.42
(C) all transcripts in in HBX
Relationships between chromatin marks in gene bodies are similar to ones in promotes. However relationships with DNA methylation appeared to be prominent from those of promoters. In all transcript s analysis, DNA methylations and H3K27Me3 have positive relationships with H3K36Me3 and CpG ratio in HBX. In comparison of the gen body pattern, as seen in the promoter pattern, negative relationships with H3K27Me3 are characterized. DNA methylation with CpG ratio and H3K4Me3 formed inverse relationships.
5/51

## Slide 6
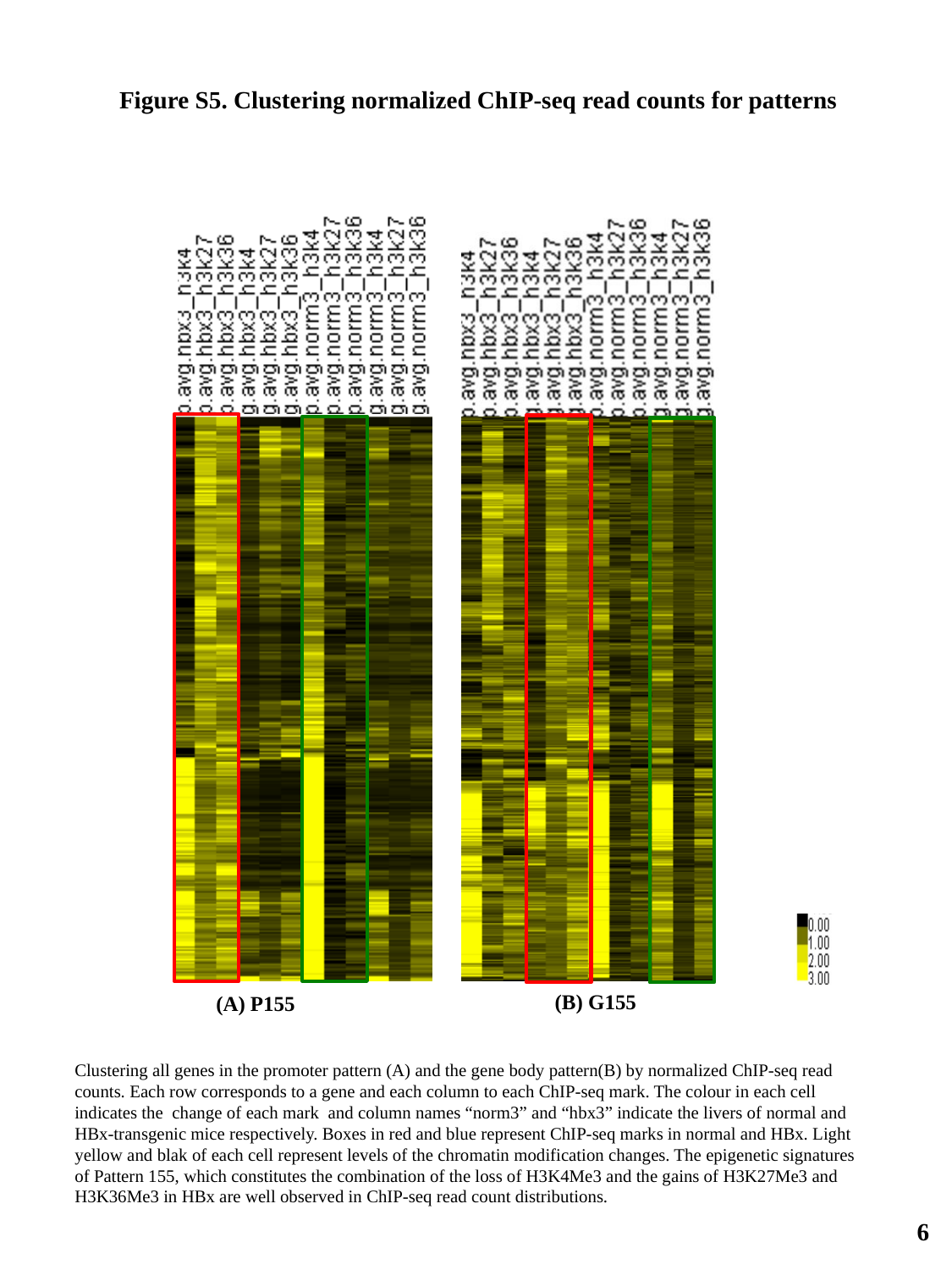

# Figure S5. Clustering normalized ChIP-seq read counts for patterns
(B) G155
(A) P155
Clustering all genes in the promoter pattern (A) and the gene body pattern(B) by normalized ChIP-seq read counts. Each row corresponds to a gene and each column to each ChIP-seq mark. The colour in each cell indicates the change of each mark and column names “norm3” and “hbx3” indicate the livers of normal and HBx-transgenic mice respectively. Boxes in red and blue represent ChIP-seq marks in normal and HBx. Light yellow and blak of each cell represent levels of the chromatin modification changes. The epigenetic signatures of Pattern 155, which constitutes the combination of the loss of H3K4Me3 and the gains of H3K27Me3 and H3K36Me3 in HBx are well observed in ChIP-seq read count distributions.
6

## Slide 7
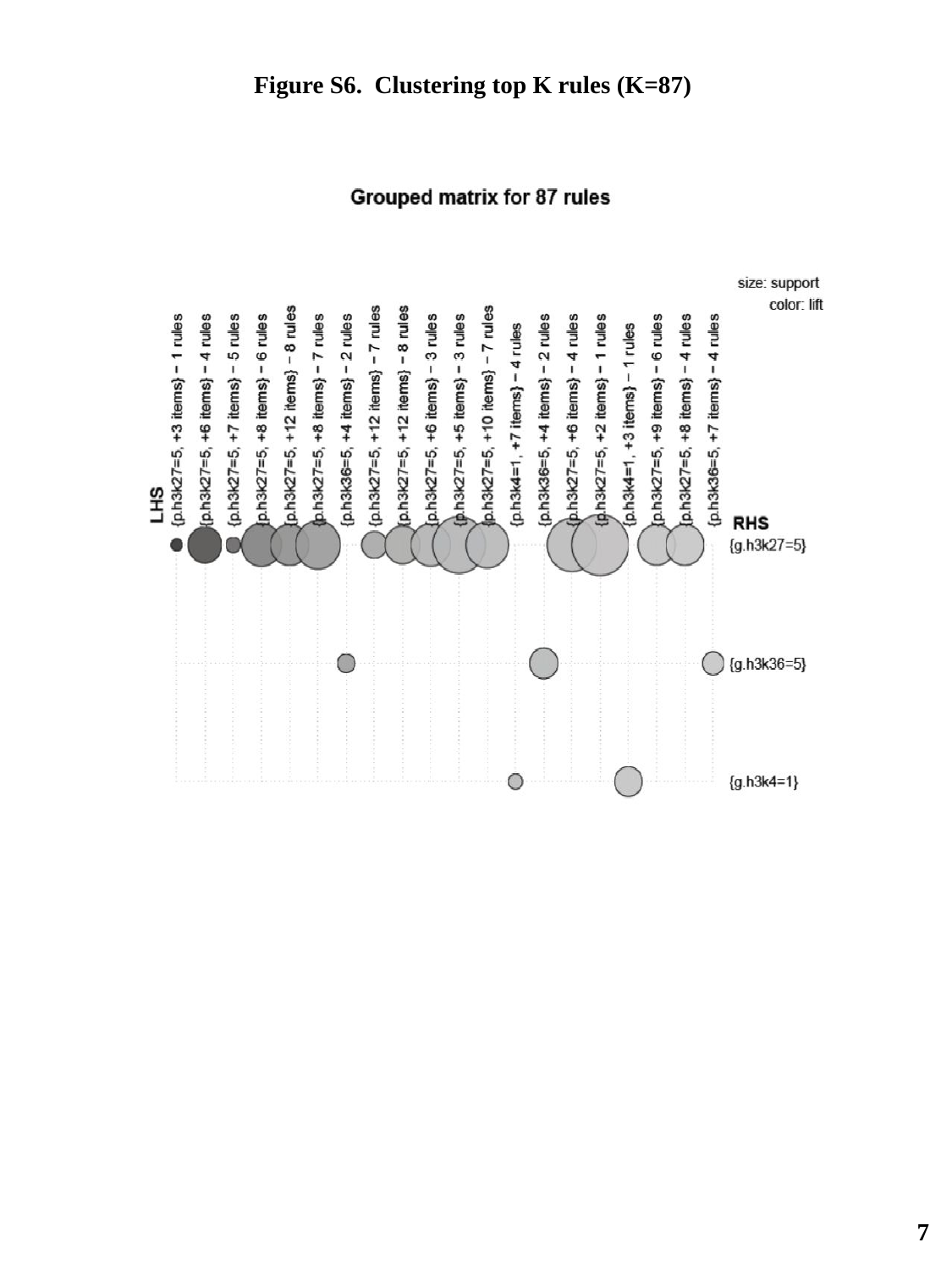

# Figure S6. Clustering top K rules (K=87)
7

## Slide 8
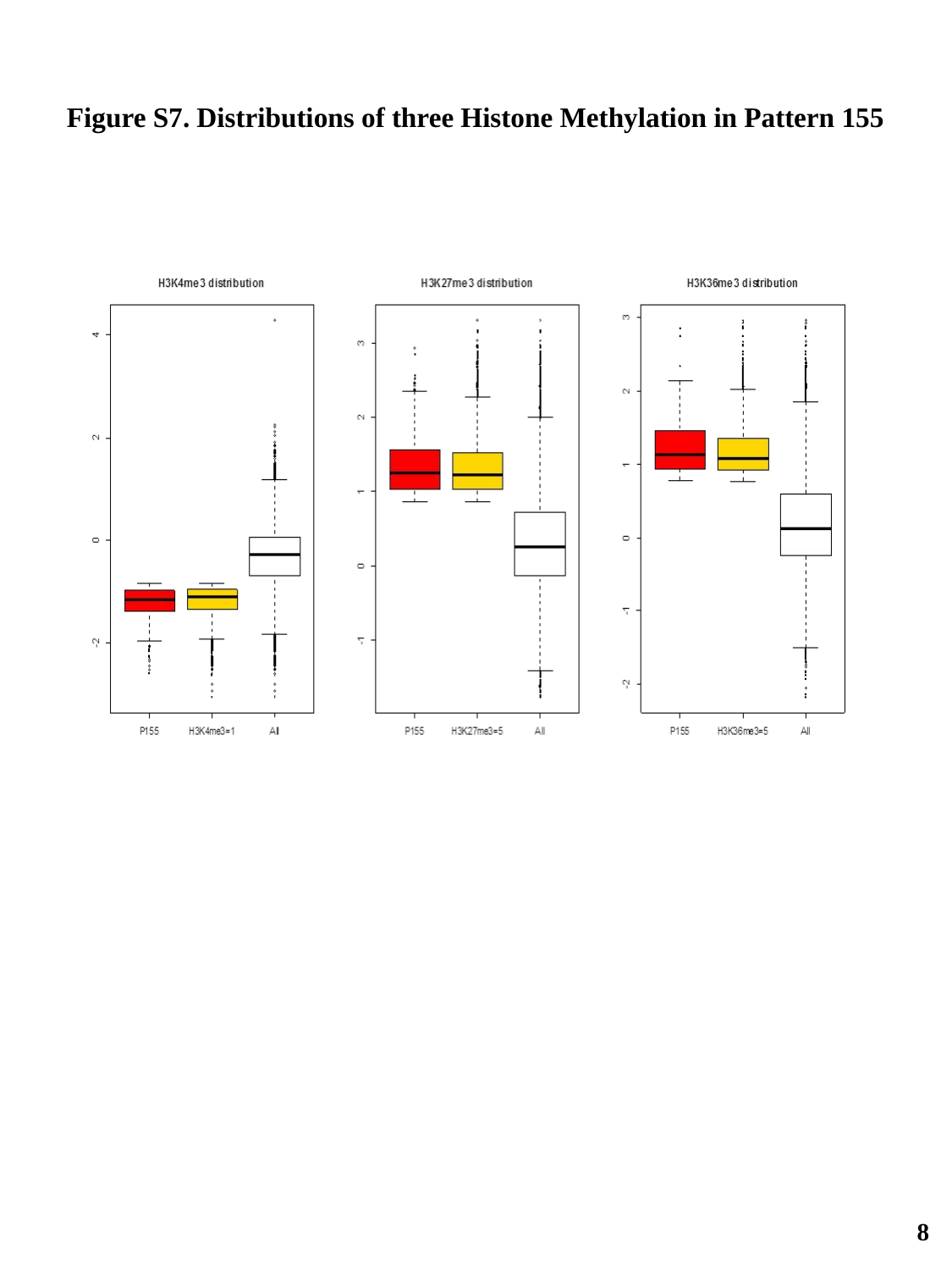

# Figure S7. Distributions of three Histone Methylation in Pattern 155
8
