# Supplementary material for: ChARM: Discovery of combinatorial chromatin modification patterns in hepatitis B virus X-transformed mouse liver cancer using association rule mining
Source: BMC Bioinformatics. 2016 Dec 13;17(Suppl 16):452. doi: 10.1186/s12859-016-1307-z (PMC5249029; doi:10.1186/s12859-016-1307-z)
Supplement: Additional file 3: — Epigenetic profiles of the patterns. Figure S1. Epigenetic modification changes of HCPs in P155. Figure S2. Epigenetic modification changes of LCPS in P155. Figure S3. Epigenetic modification changes of random HCPs (n = 1000). Figure S4. Epigenetic modification changes of random LCPs (n = 1000). Figure S5. CpG distributions of HCPs and LCPs in P155 and pattern matching region. Figure S6. CpG distributions of groups composing of chromatin mark changes. (PPTX 772 kb) [file 12859_2016_1307_MOESM3_ESM.pptx]

## Slide 1
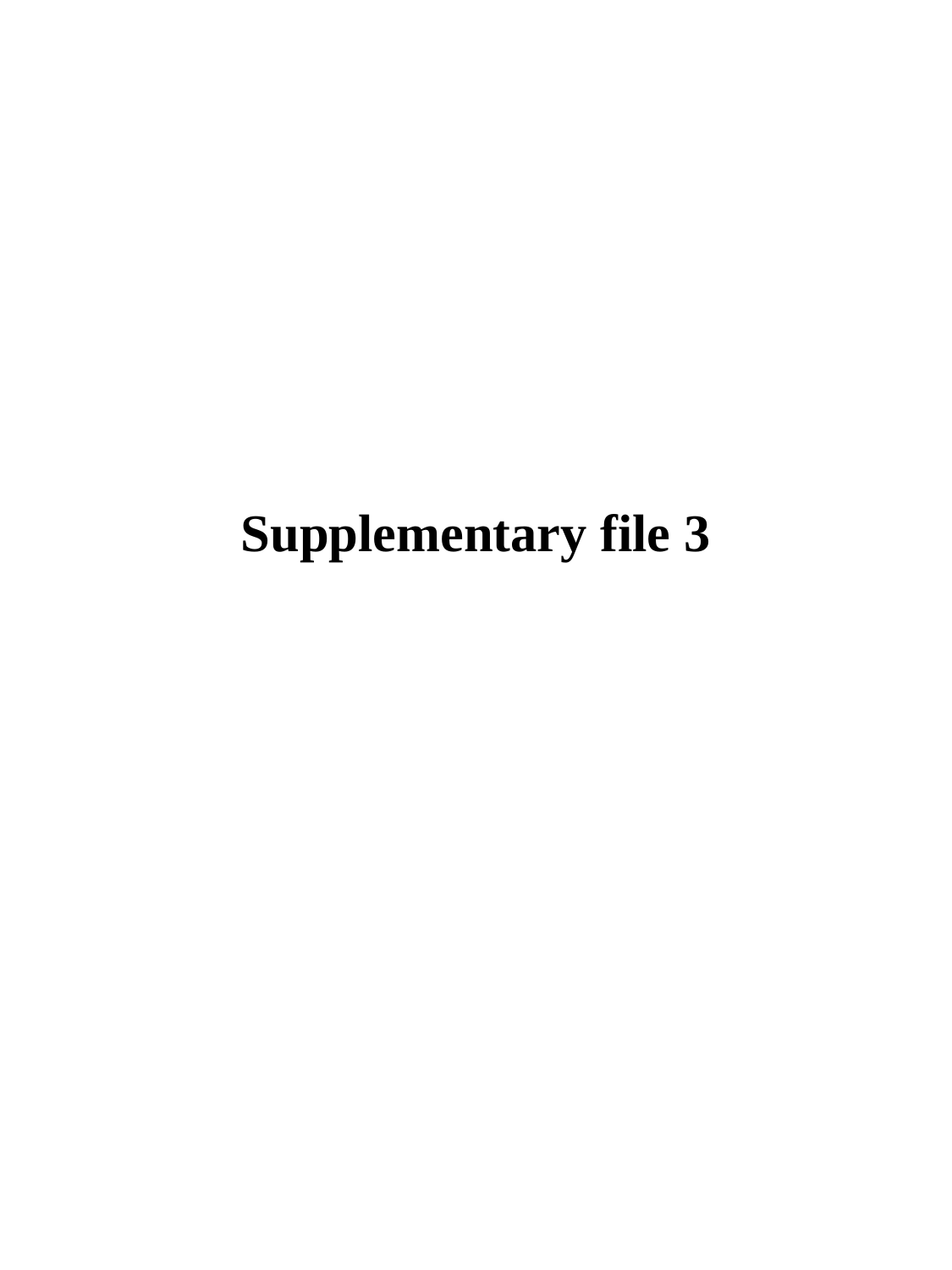

# Supplementary file 3

## Slide 2
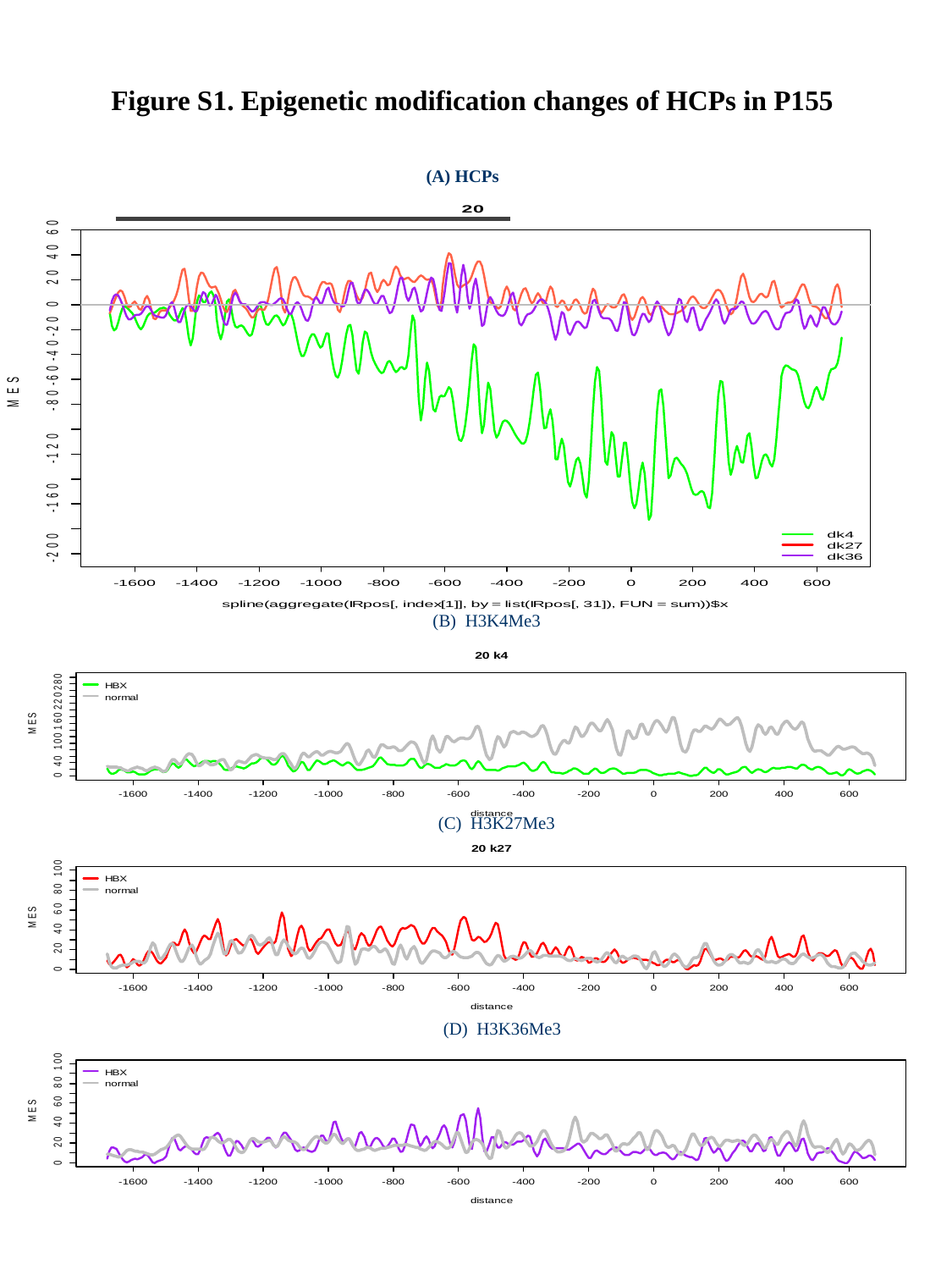

# Figure S1. Epigenetic modification changes of HCPs in P155
(A) HCPs
(B) H3K4Me3
(C) H3K27Me3
(D) H3K36Me3

## Slide 3
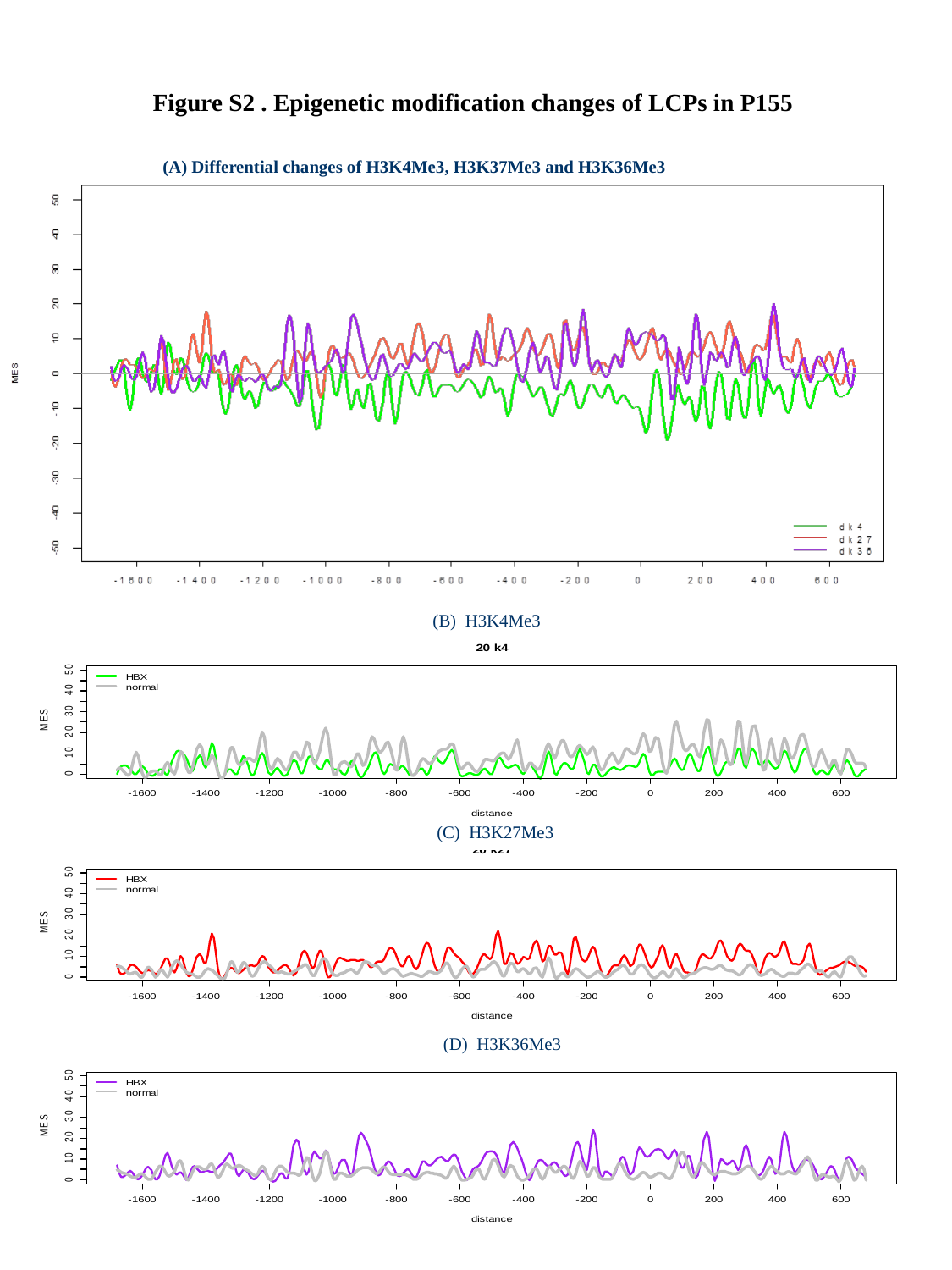

# Figure S2 . Epigenetic modification changes of LCPs in P155
(A) Differential changes of H3K4Me3, H3K37Me3 and H3K36Me3
(B) H3K4Me3
(C) H3K27Me3
(D) H3K36Me3

## Slide 4
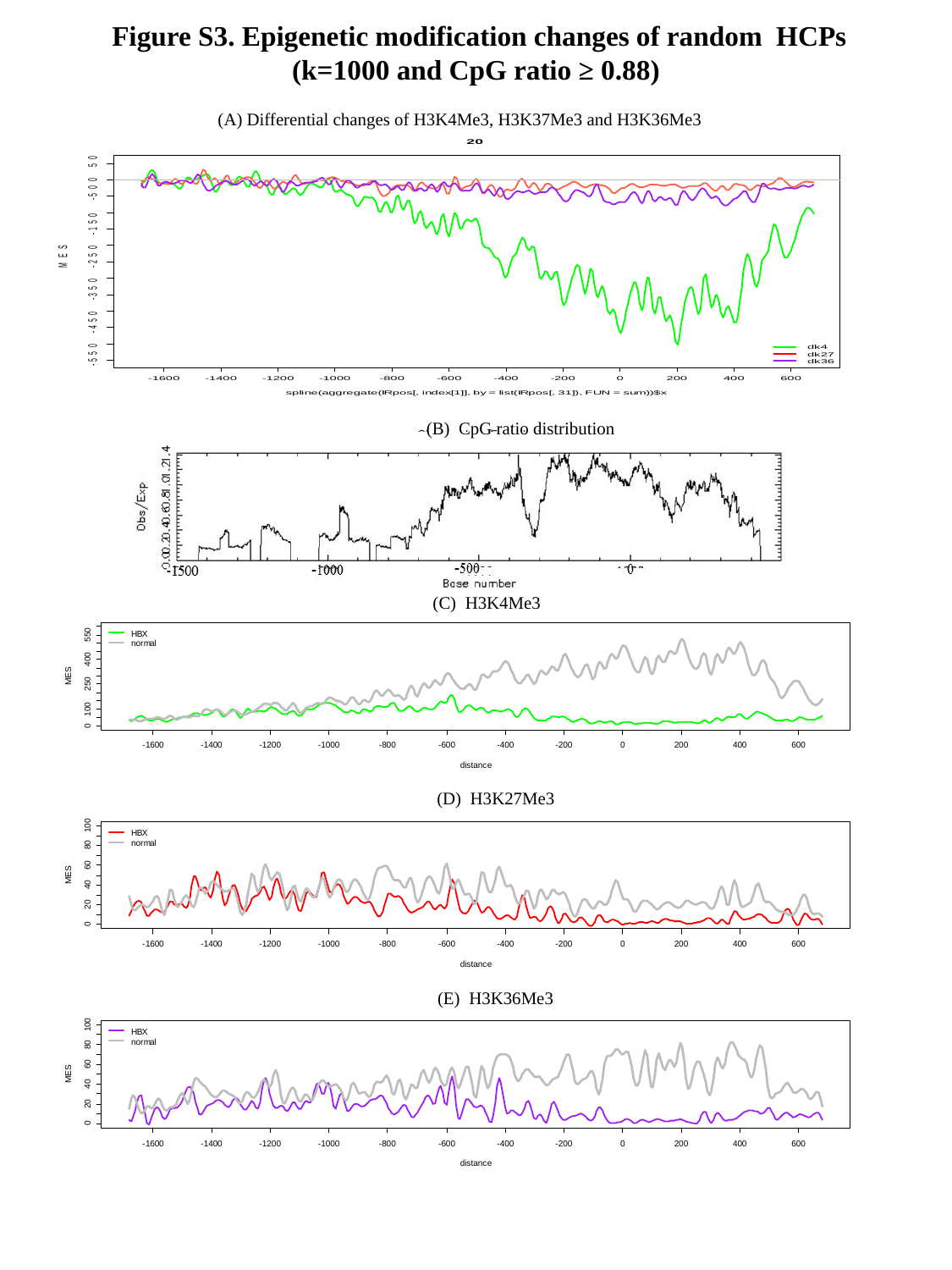

# Figure S3. Epigenetic modification changes of random HCPs (k=1000 and CpG ratio ≥ 0.88)
(A) Differential changes of H3K4Me3, H3K37Me3 and H3K36Me3
(B) CpG ratio distribution
(C) H3K4Me3
(D) H3K27Me3
(E) H3K36Me3

## Slide 5
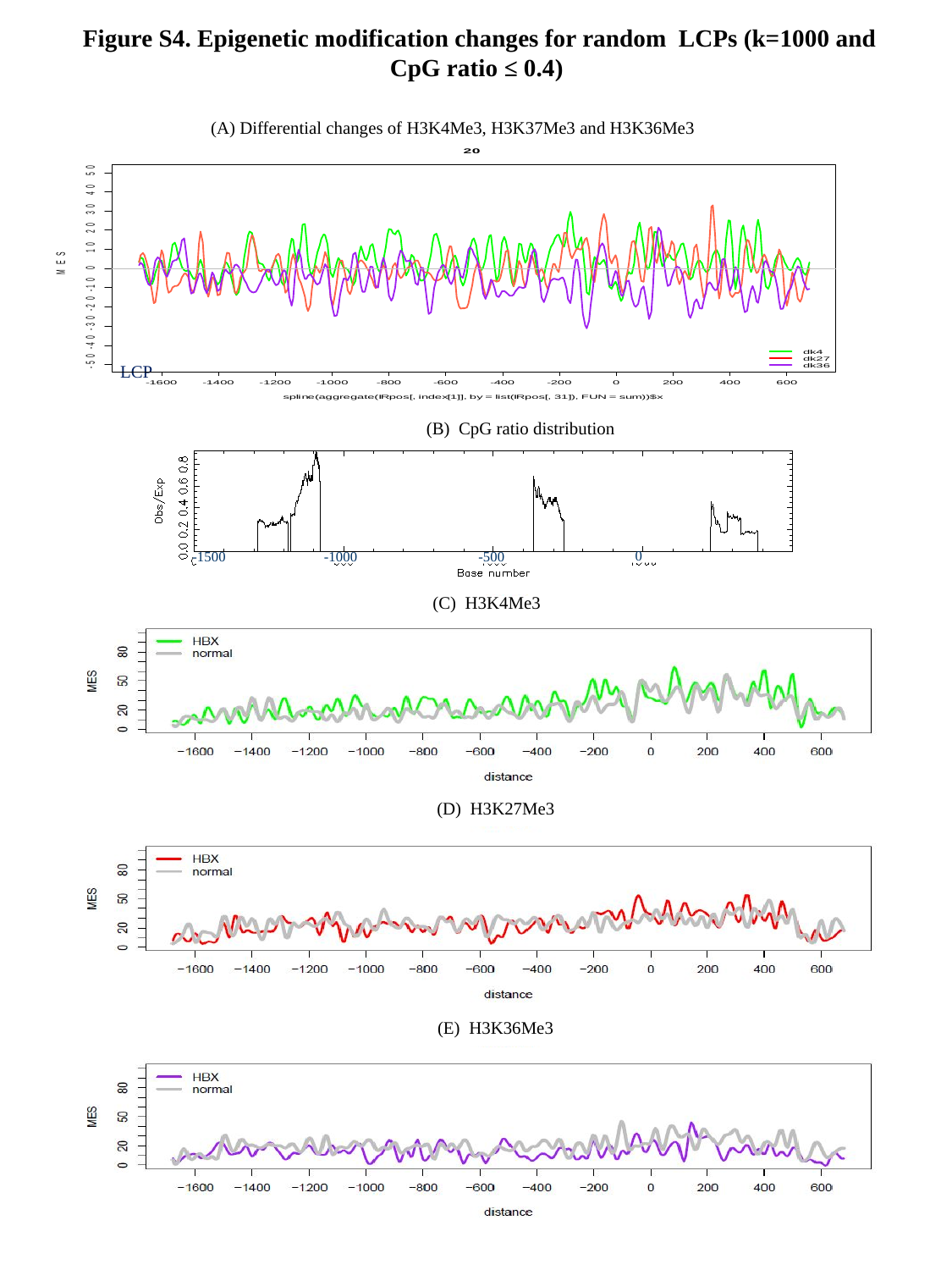

# Figure S4. Epigenetic modification changes for random LCPs (k=1000 and CpG ratio ≤ 0.4)
(A) Differential changes of H3K4Me3, H3K37Me3 and H3K36Me3
LCP
(B) CpG ratio distribution
-1500
 0
-1000
-500
(C) H3K4Me3
(D) H3K27Me3
(E) H3K36Me3

## Slide 6
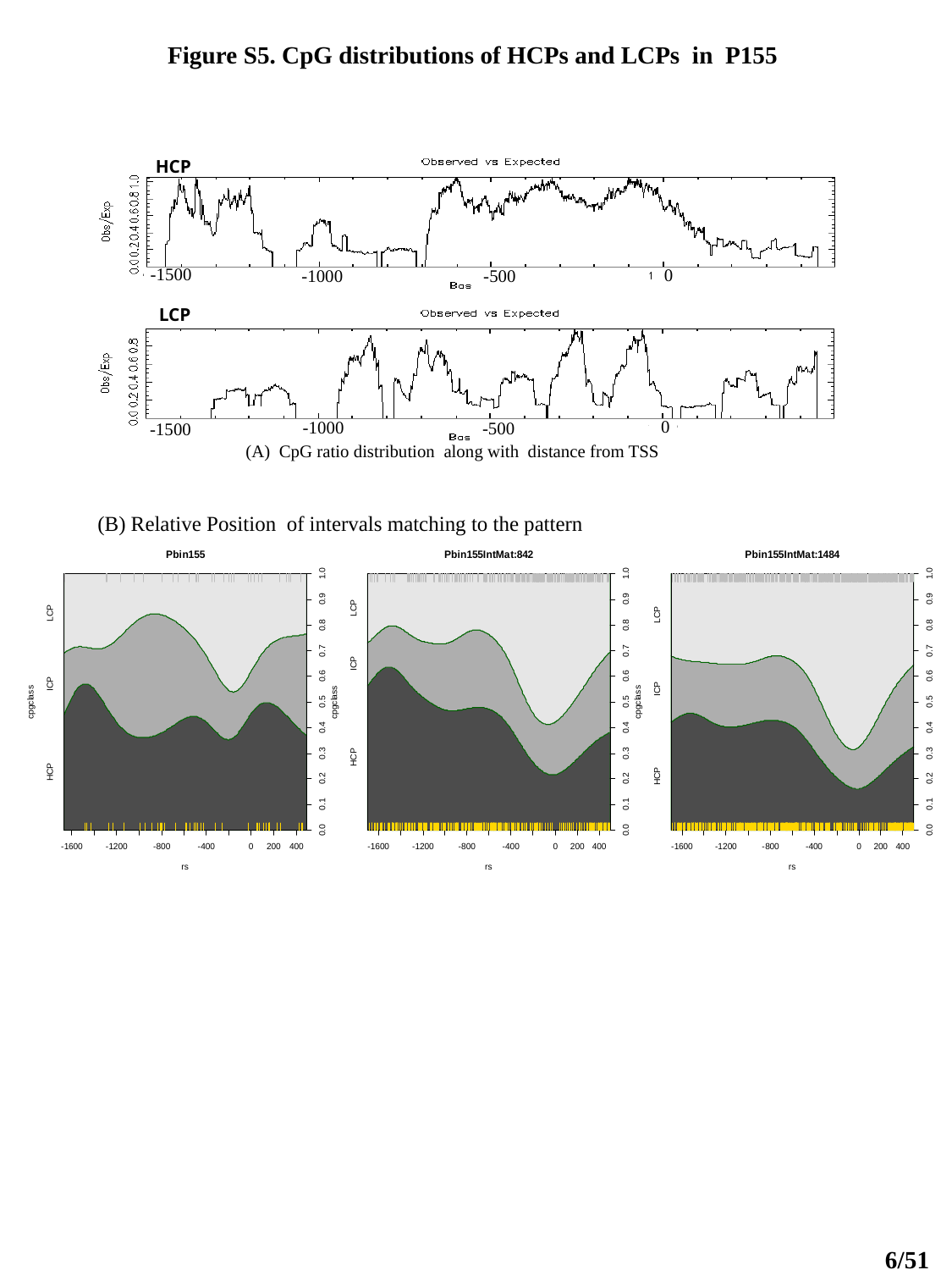

# Figure S5. CpG distributions of HCPs and LCPs in P155
HCP
0
-500
-1000
-1500
LCP
0
-1000
-500
-1500
(A) CpG ratio distribution along with distance from TSS
(B) Relative Position of intervals matching to the pattern
6/51

## Slide 7
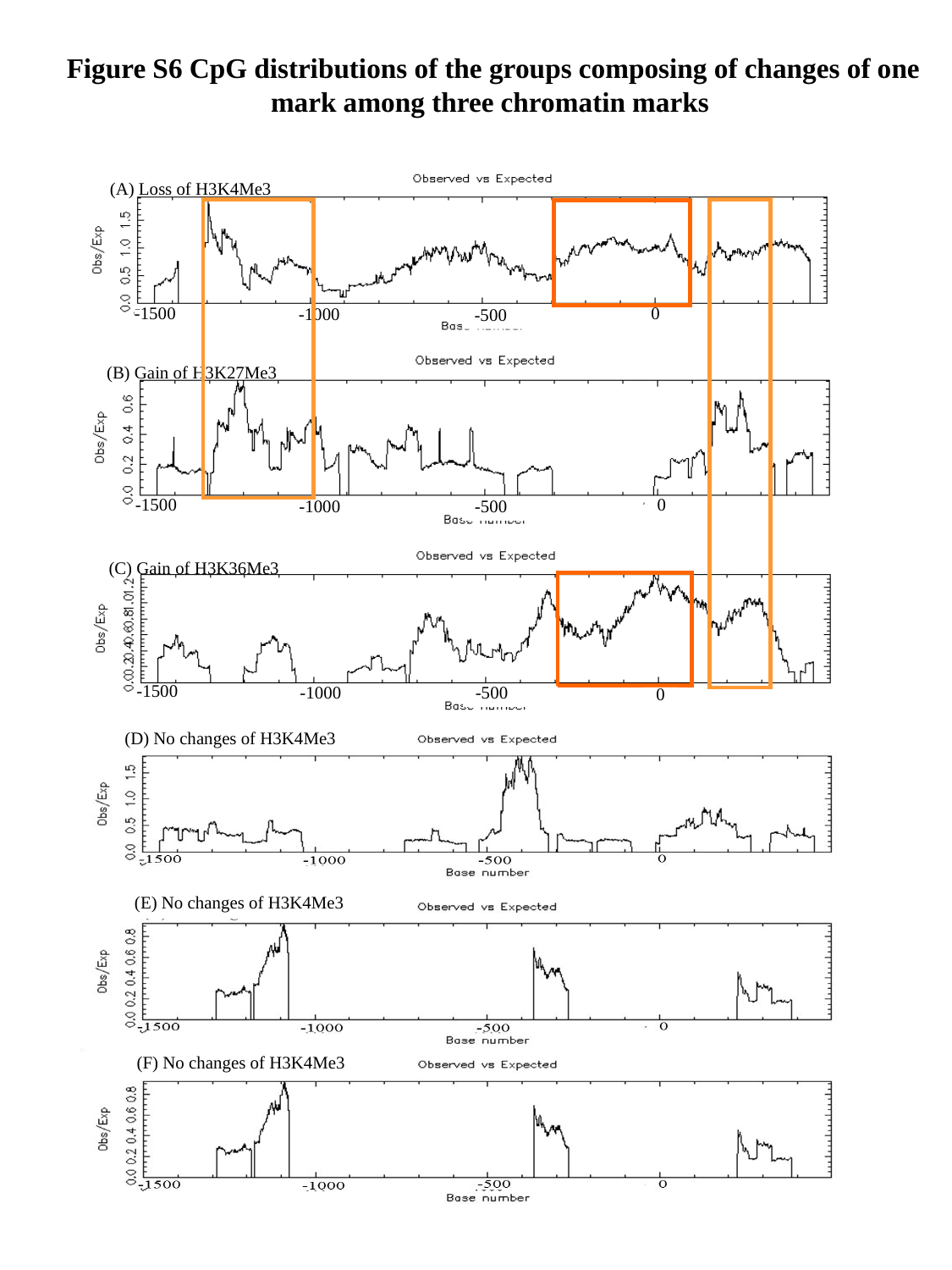

# Figure S6 CpG distributions of the groups composing of changes of one mark among three chromatin marks
(A) Loss of H3K4Me3
0
-1000
-500
-1500
(B) Gain of H3K27Me3
0
-1000
-500
-1500
(C) Gain of H3K36Me3
-1000
-500
0
-1500
(D) No changes of H3K4Me3
(E) No changes of H3K4Me3
(F) No changes of H3K4Me3
