# Supplementary material for: ChARM: Discovery of combinatorial chromatin modification patterns in hepatitis B virus X-transformed mouse liver cancer using association rule mining
Source: BMC Bioinformatics. 2016 Dec 13;17(Suppl 16):452. doi: 10.1186/s12859-016-1307-z (PMC5249029; doi:10.1186/s12859-016-1307-z)
Supplement: Additional file 4: — Characterisation and interpretation of the patterns. Figure S1 Enrichment of CGI in the pattern. Figure S2. Pol2SII changes in the gene body pattern. Figure S3. DNA methylation changes in the promoter pattern. Figure S4. Differential gene expression. Figure S5. Gene expression vs. histone modification marks in the pattern. Figure S6. Epigenetic changes of each transcript in the promoter pattern. Figure S7. Epigenetic changes of each transcript in the gene body pattern. (PPTX 1164 kb) [file 12859_2016_1307_MOESM4_ESM.pptx]

## Slide 1
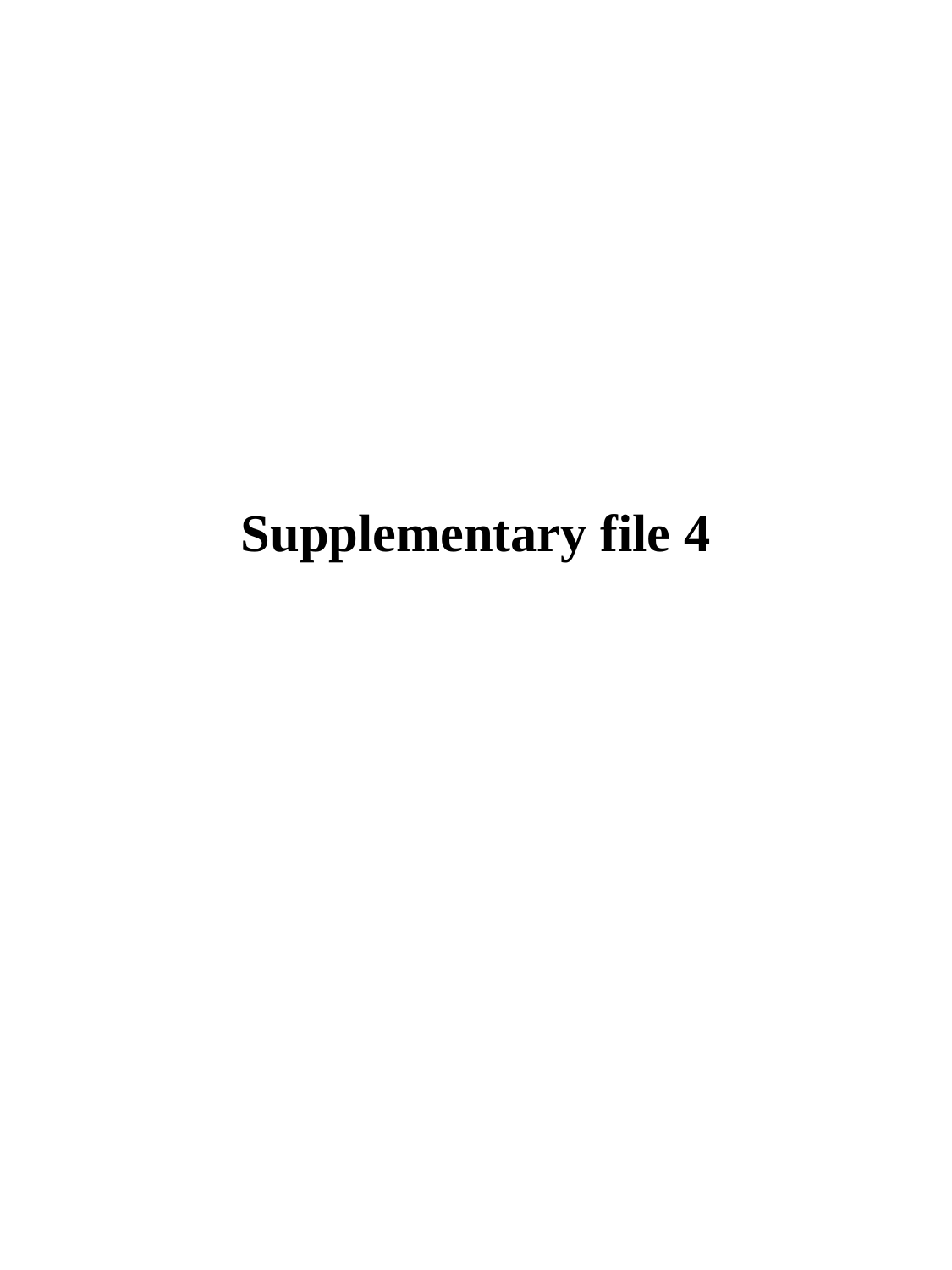

# Supplementary file 4

## Slide 2
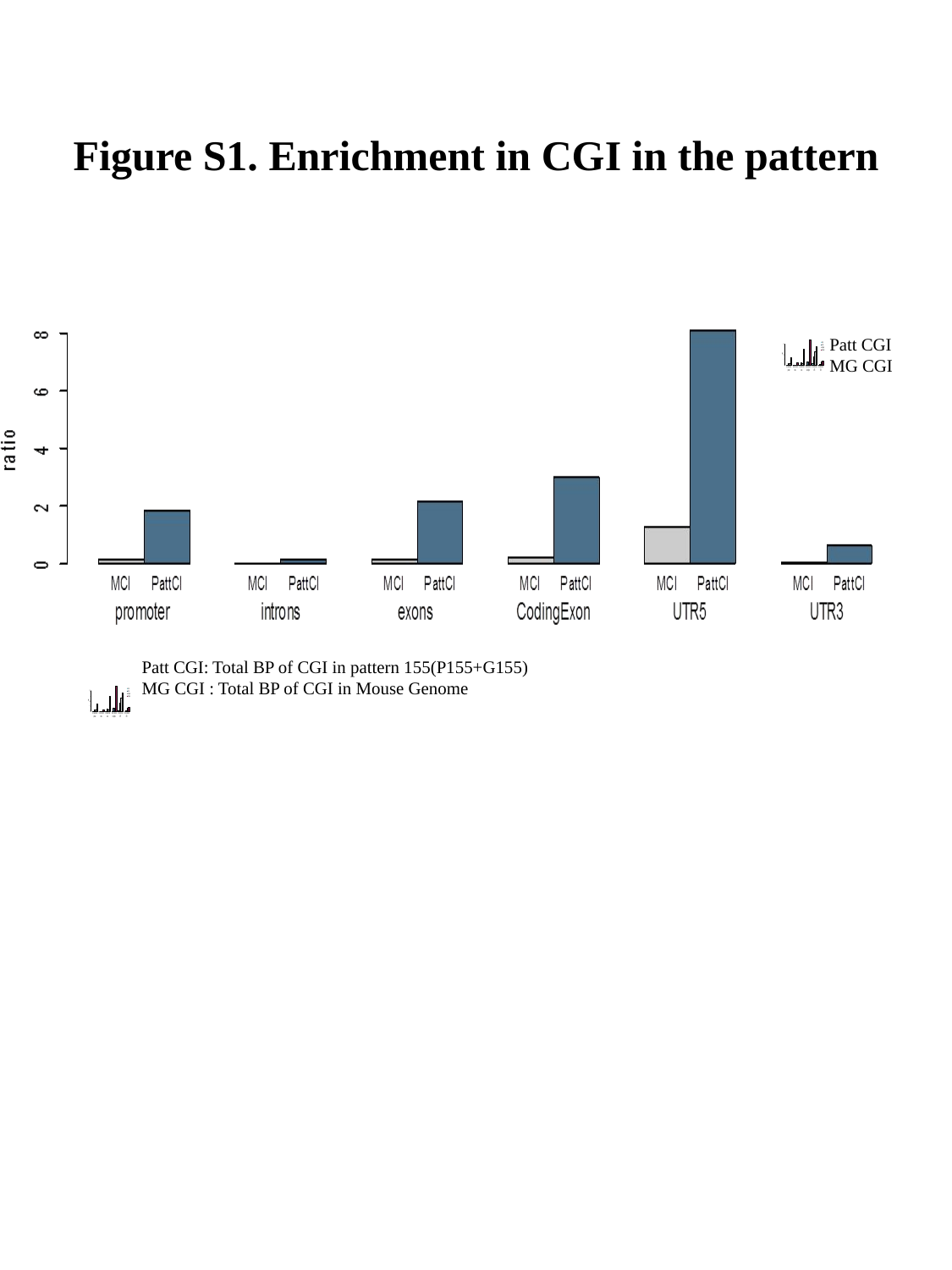

# Figure S1. Enrichment in CGI in the pattern
Patt CGI
MG CGI
Patt CGI: Total BP of CGI in pattern 155(P155+G155)
MG CGI : Total BP of CGI in Mouse Genome

## Slide 3
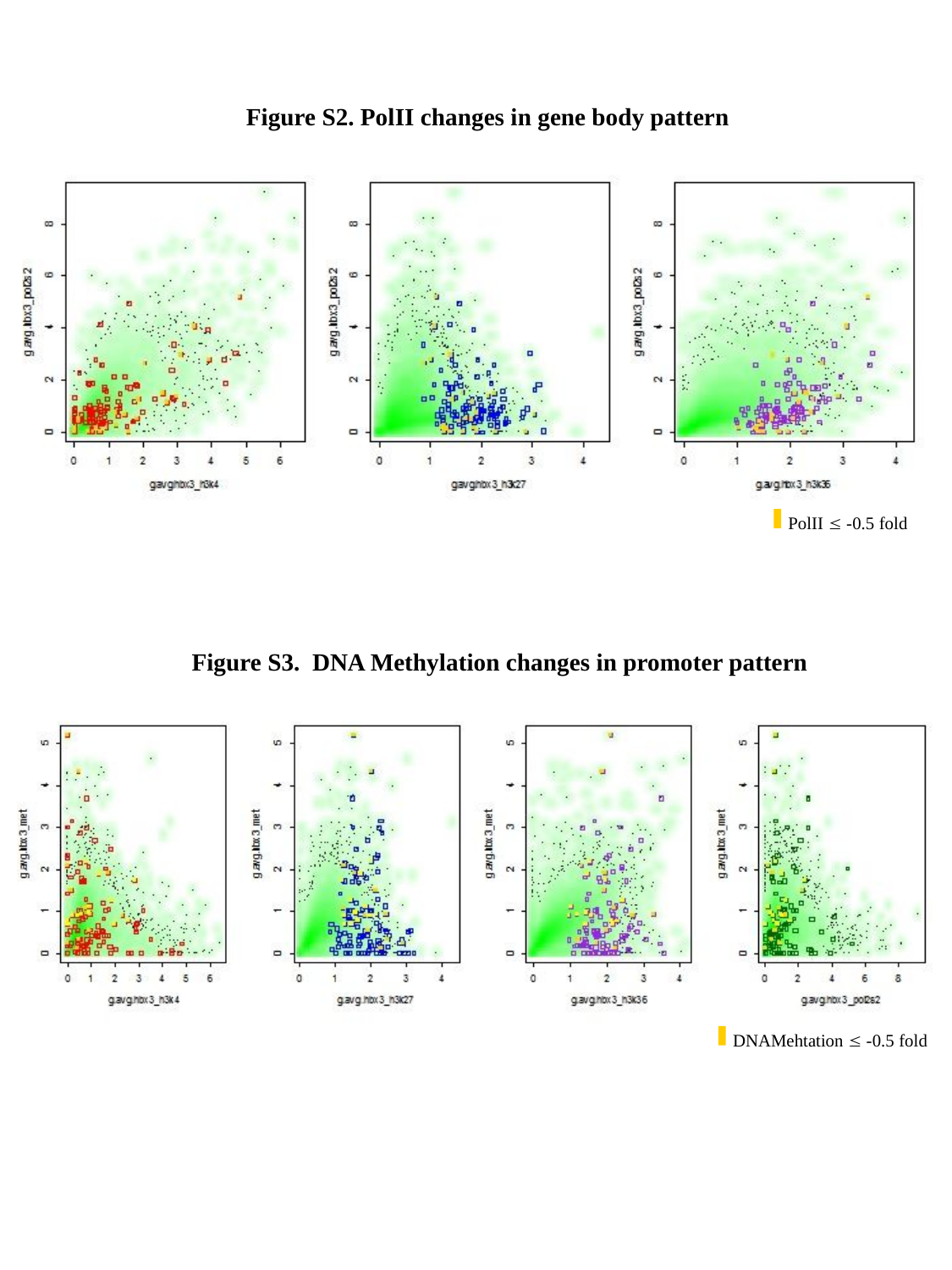

# Figure S2. PolII changes in gene body pattern
PolII ≤ -0.5 fold
Figure S3. DNA Methylation changes in promoter pattern
DNAMehtation ≤ -0.5 fold

## Slide 4
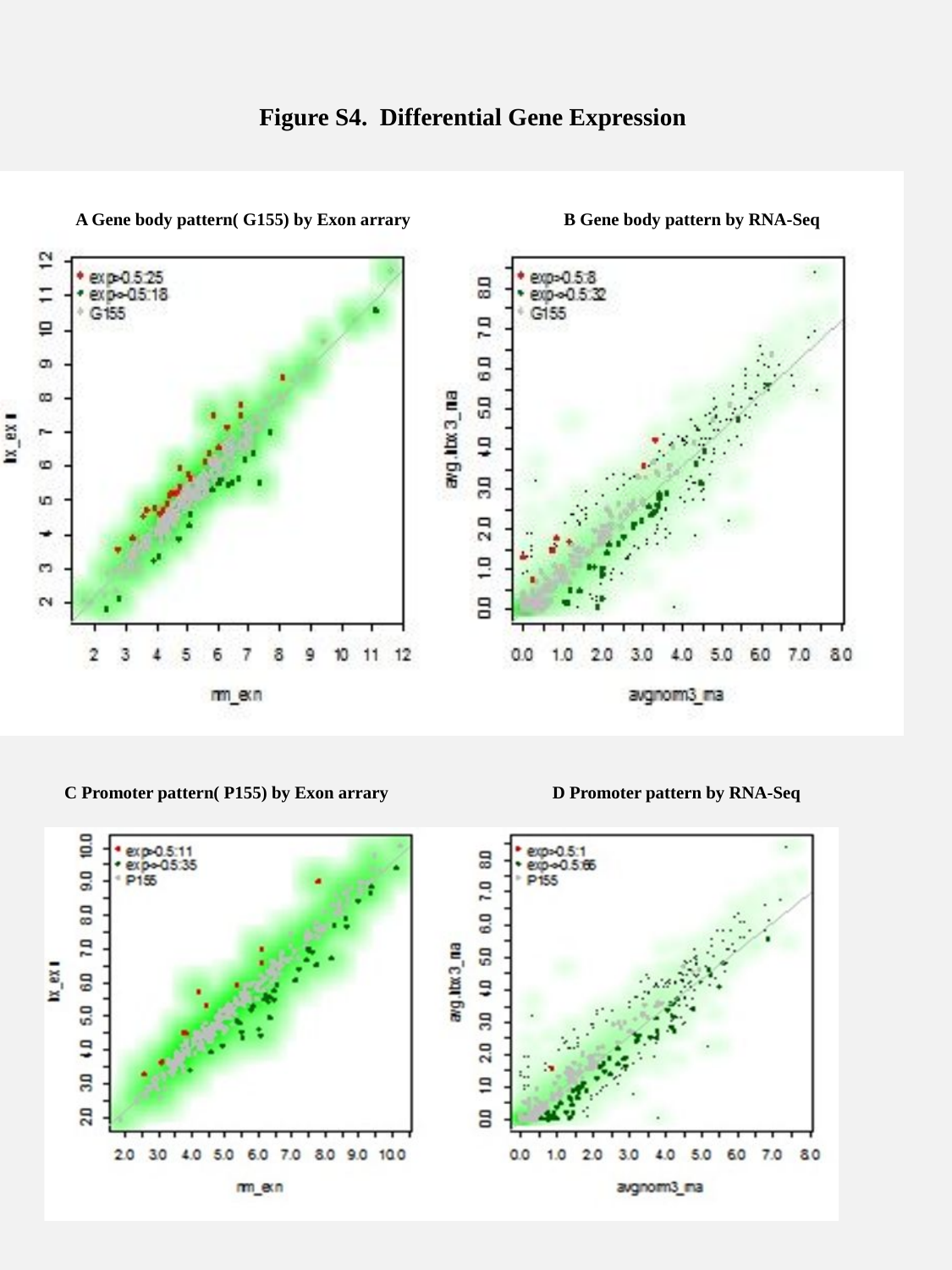

# Figure S4. Differential Gene Expression
B Gene body pattern by RNA-Seq
A Gene body pattern( G155) by Exon arrary
D Promoter pattern by RNA-Seq
C Promoter pattern( P155) by Exon arrary

## Slide 5
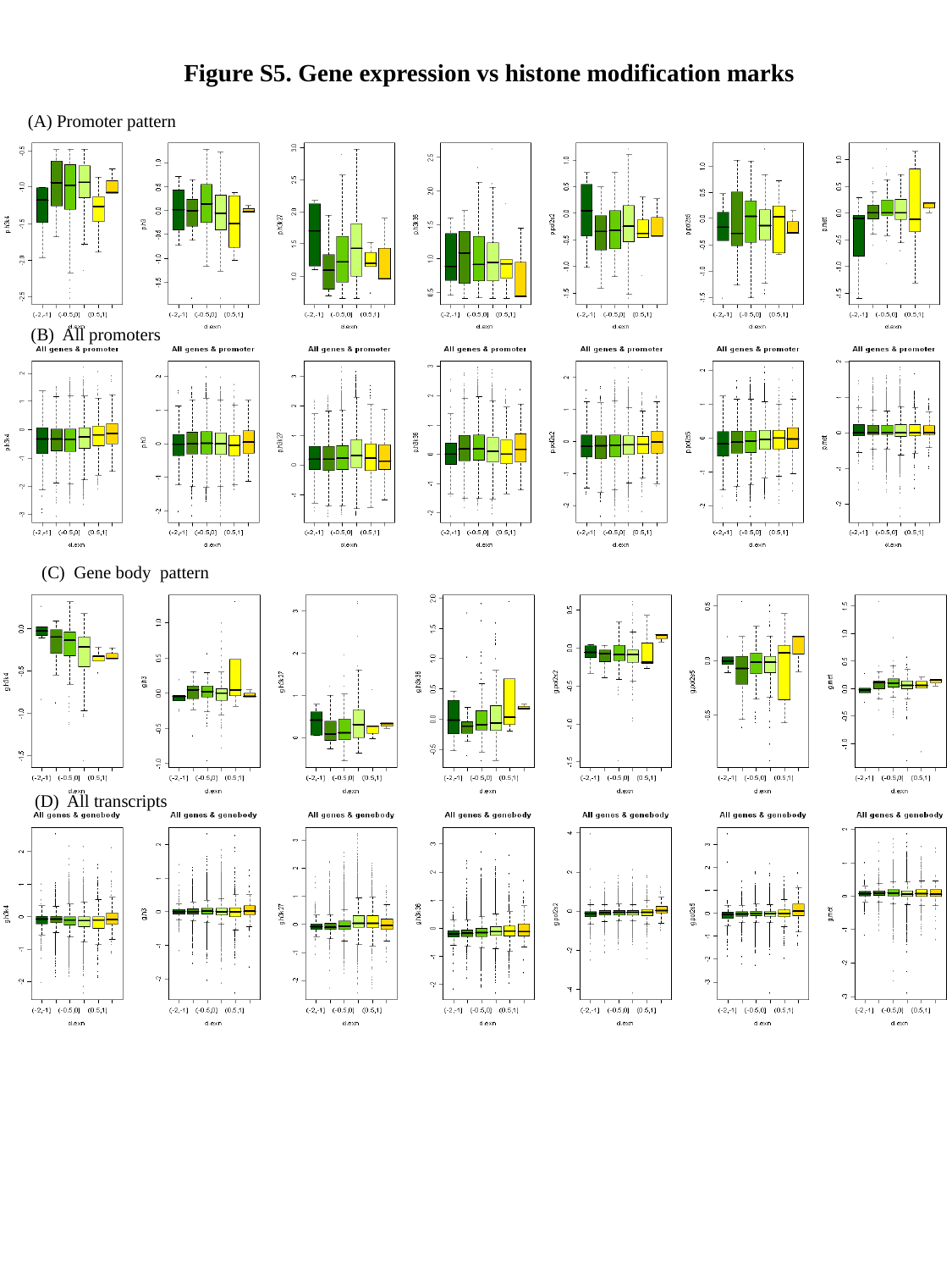

# Figure S5. Gene expression vs histone modification marks
(A) Promoter pattern
(B) All promoters
(C) Gene body pattern
(D) All transcripts

## Slide 6
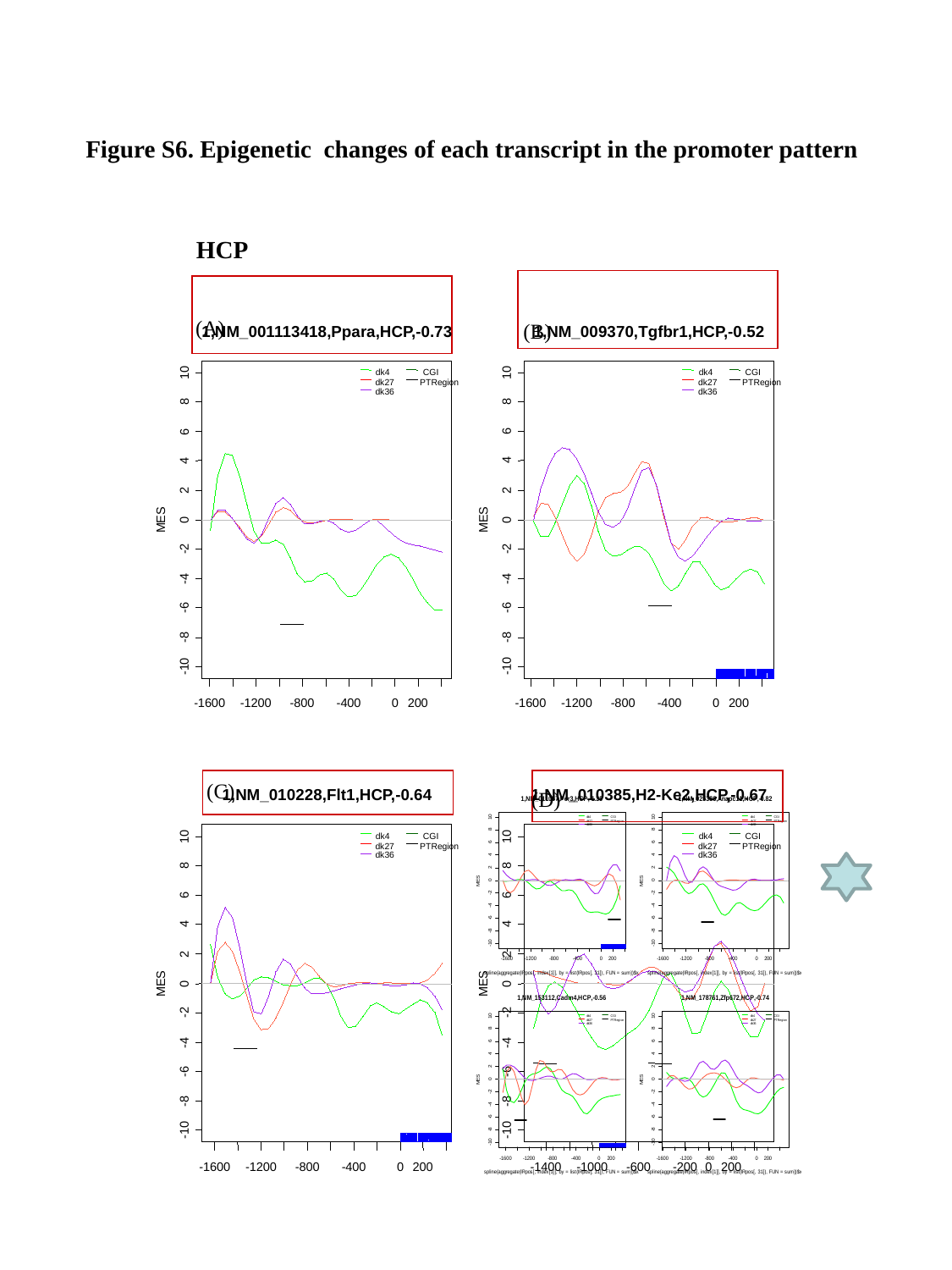

# Figure S6. Epigenetic changes of each transcript in the promoter pattern
HCP
1,NM_001113418,Ppara,HCP,-0.73
1,NM_009370,Tgfbr1,HCP,-0.52
10
10
dk4
CGI
dk27
PTRegion
dk36
8
8
6
6
4
4
2
2
MES
0
MES
0
-2
-2
-4
-4
-6
-6
-8
-8
-10
-10
-1600
-1200
-800
-400
0
200
-1600
-1200
-800
-400
0
200
dk4
CGI
dk27
PTRegion
dk36
1,NM_010228,Flt1,HCP,-0.64
10
8
6
4
2
MES
0
-2
-4
-6
-8
-10
-1600
-1200
-800
-400
0
200
1,NM_010385,H2-Ke2,HCP,-0.67
10
dk4
CGI
dk4
CGI
dk27
PTRegion
dk27
PTRegion
dk36
dk36
8
6
4
2
MES
0
-2
-4
-6
-8
-10
-1400
-1000
-600
-200
0
200
(A)
(B)
(C)
(D)

## Slide 7
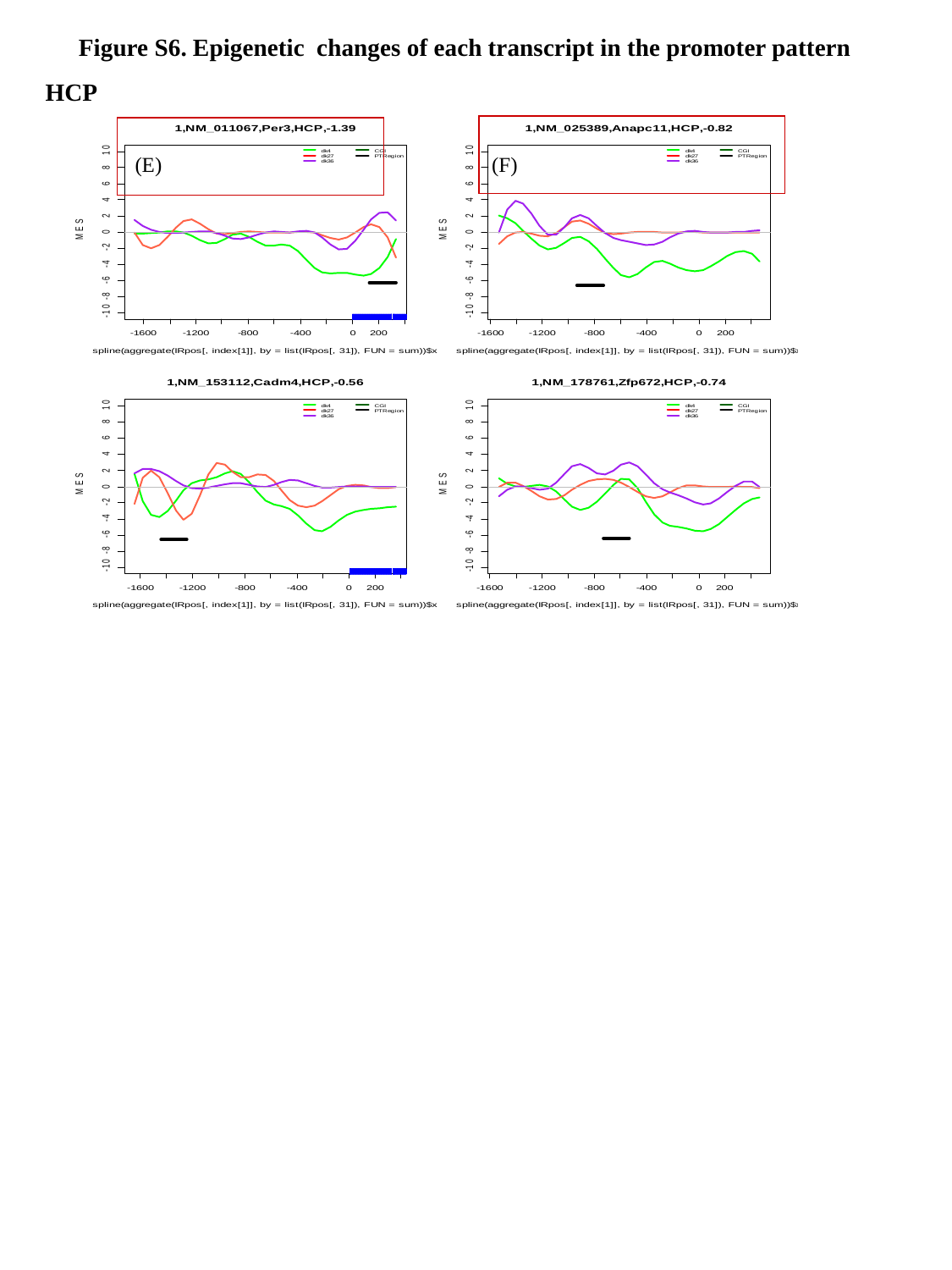

# Figure S6. Epigenetic changes of each transcript in the promoter pattern
HCP
(F)
(E)

## Slide 8
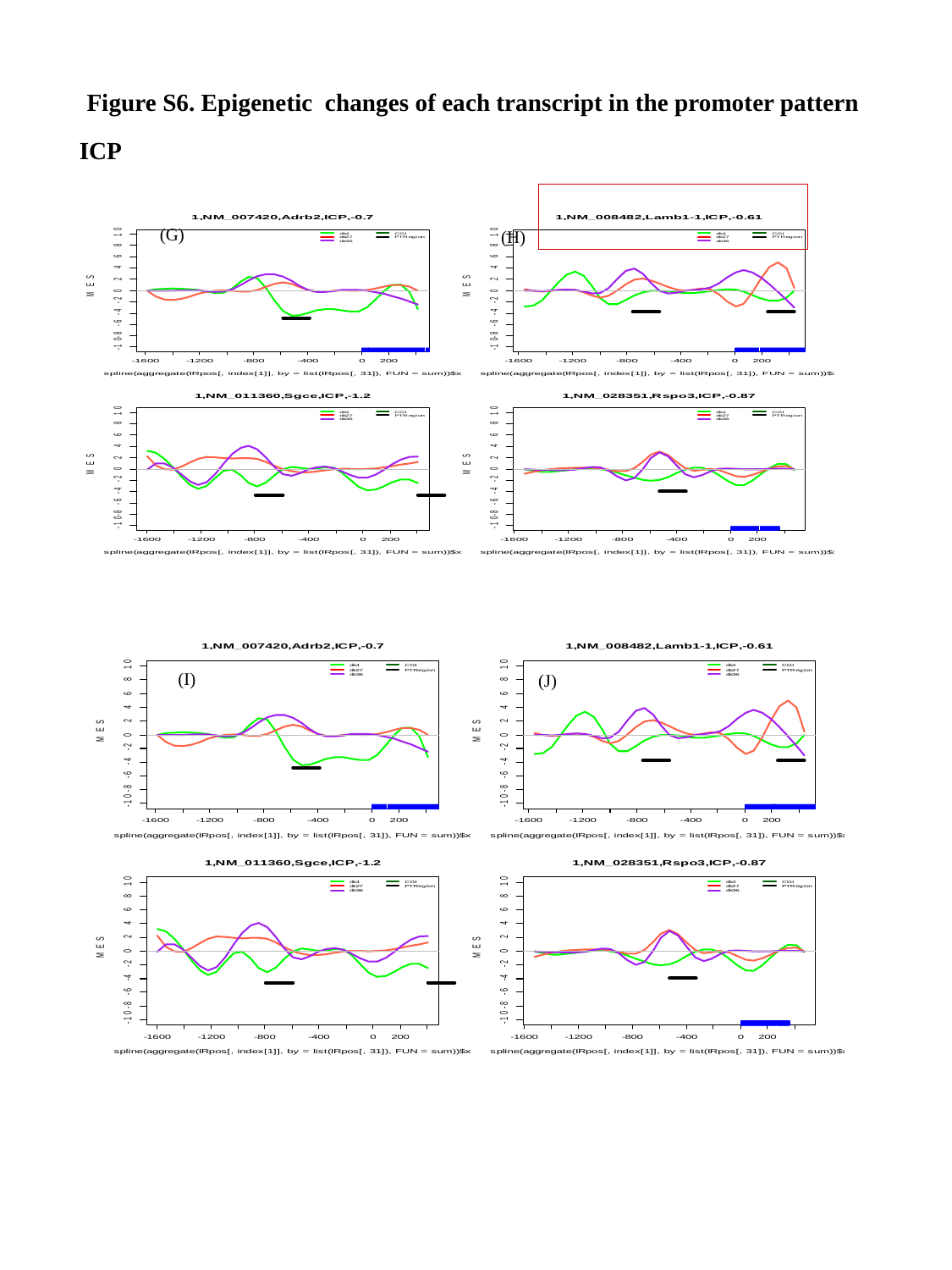

# Figure S6. Epigenetic changes of each transcript in the promoter pattern
ICP
(G)
(H)
(I)
(J)

## Slide 9
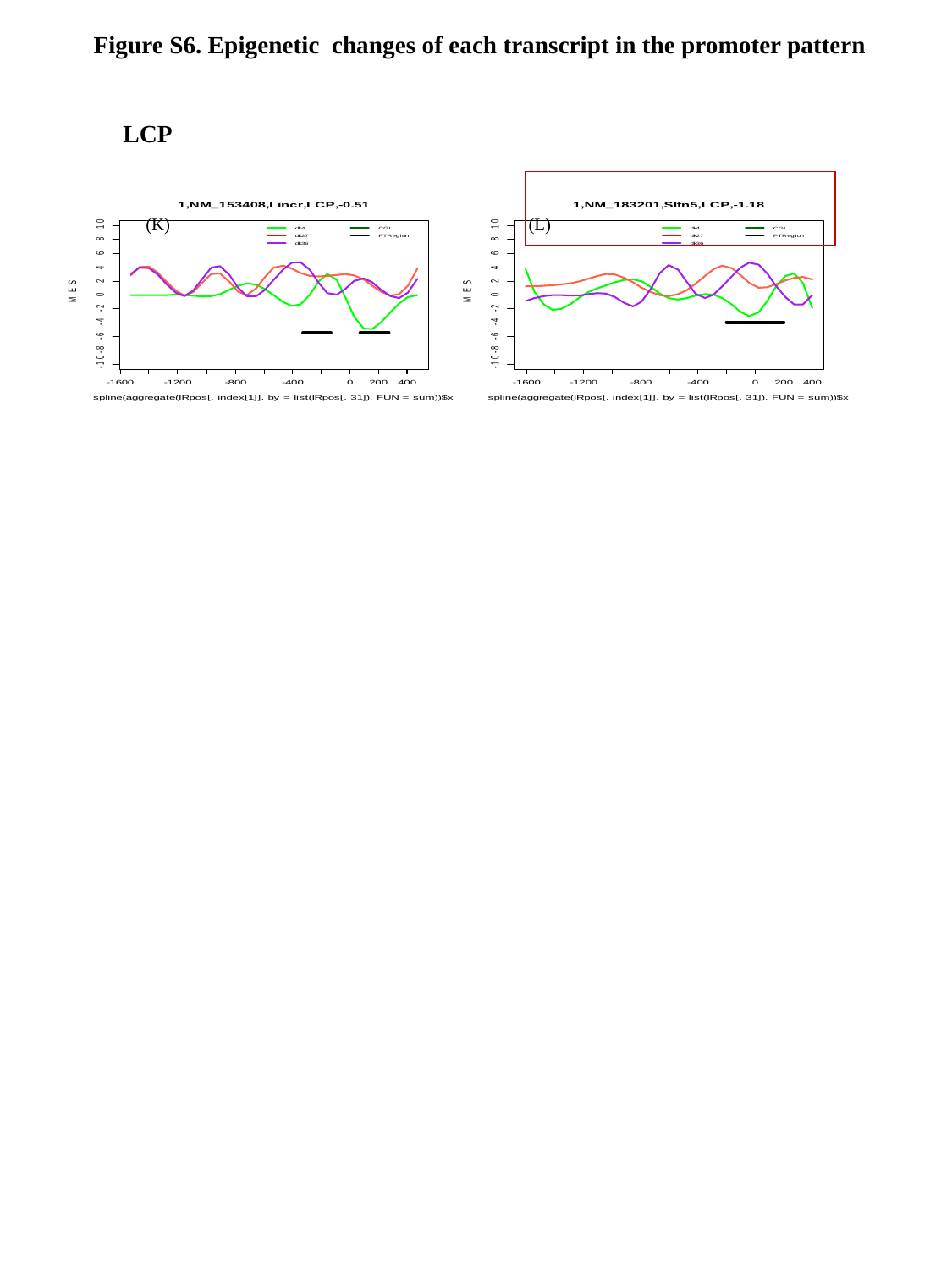

Figure S6. Epigenetic changes of each transcript in the promoter pattern
# LCP
(K)
(L)

## Slide 10
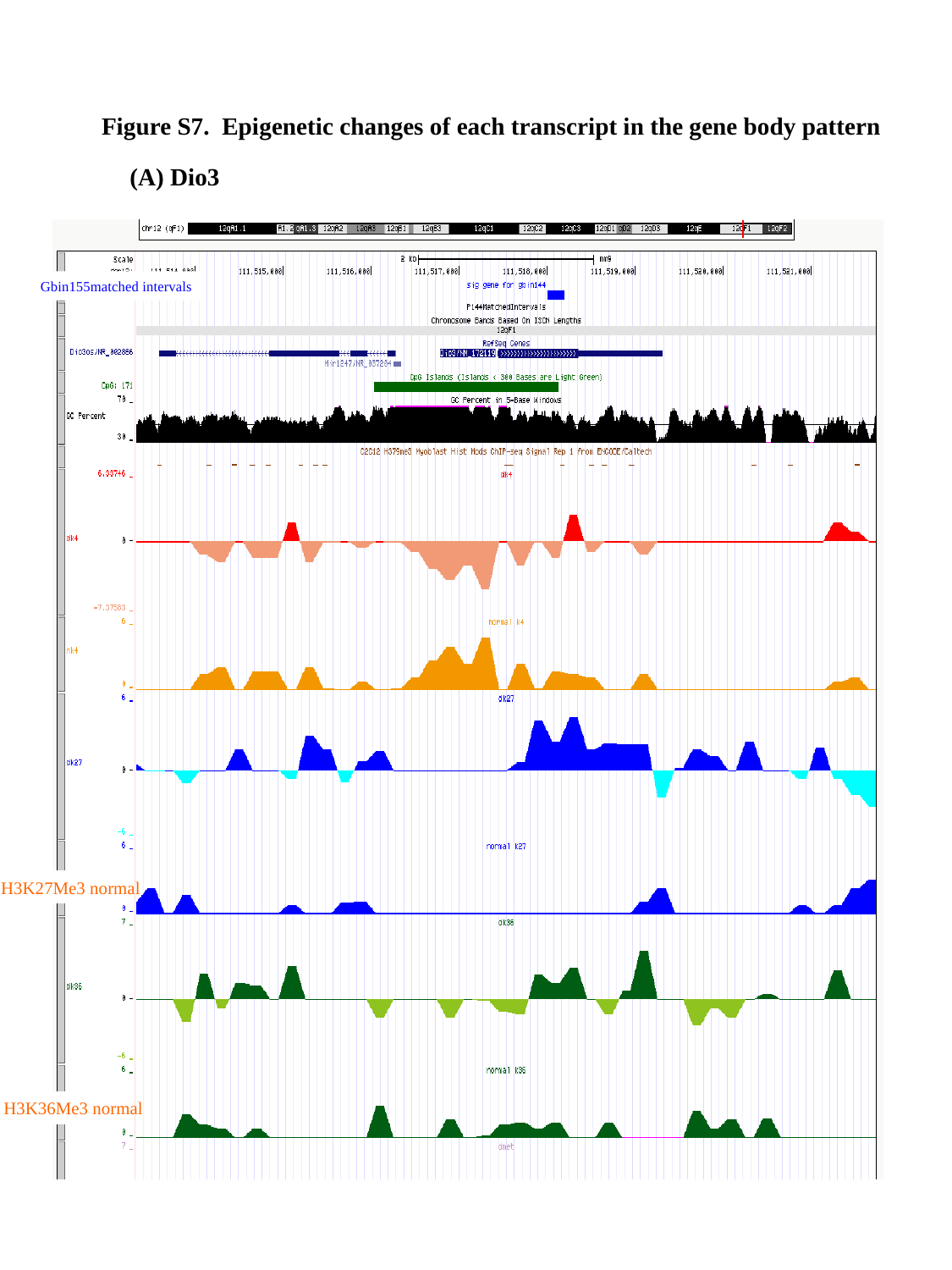

Figure S7. Epigenetic changes of each transcript in the gene body pattern
# (A) Dio3
Gbin155matched intervals
H3K27Me3 normal
H3K36Me3 normal

## Slide 11
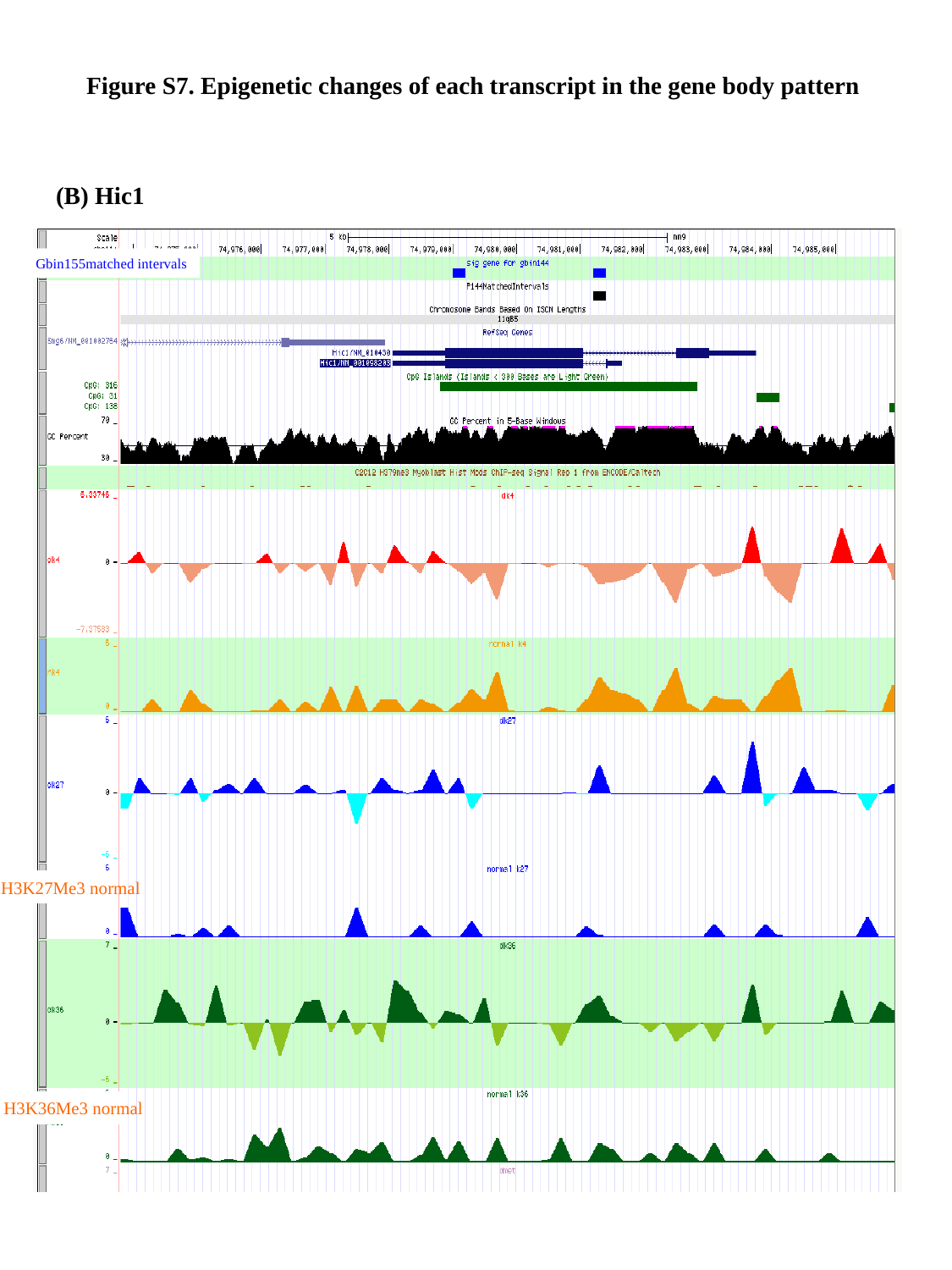

Figure S7. Epigenetic changes of each transcript in the gene body pattern
(B) Hic1
Gbin155matched intervals
H3K27Me3 normal
H3K36Me3 normal

## Slide 12
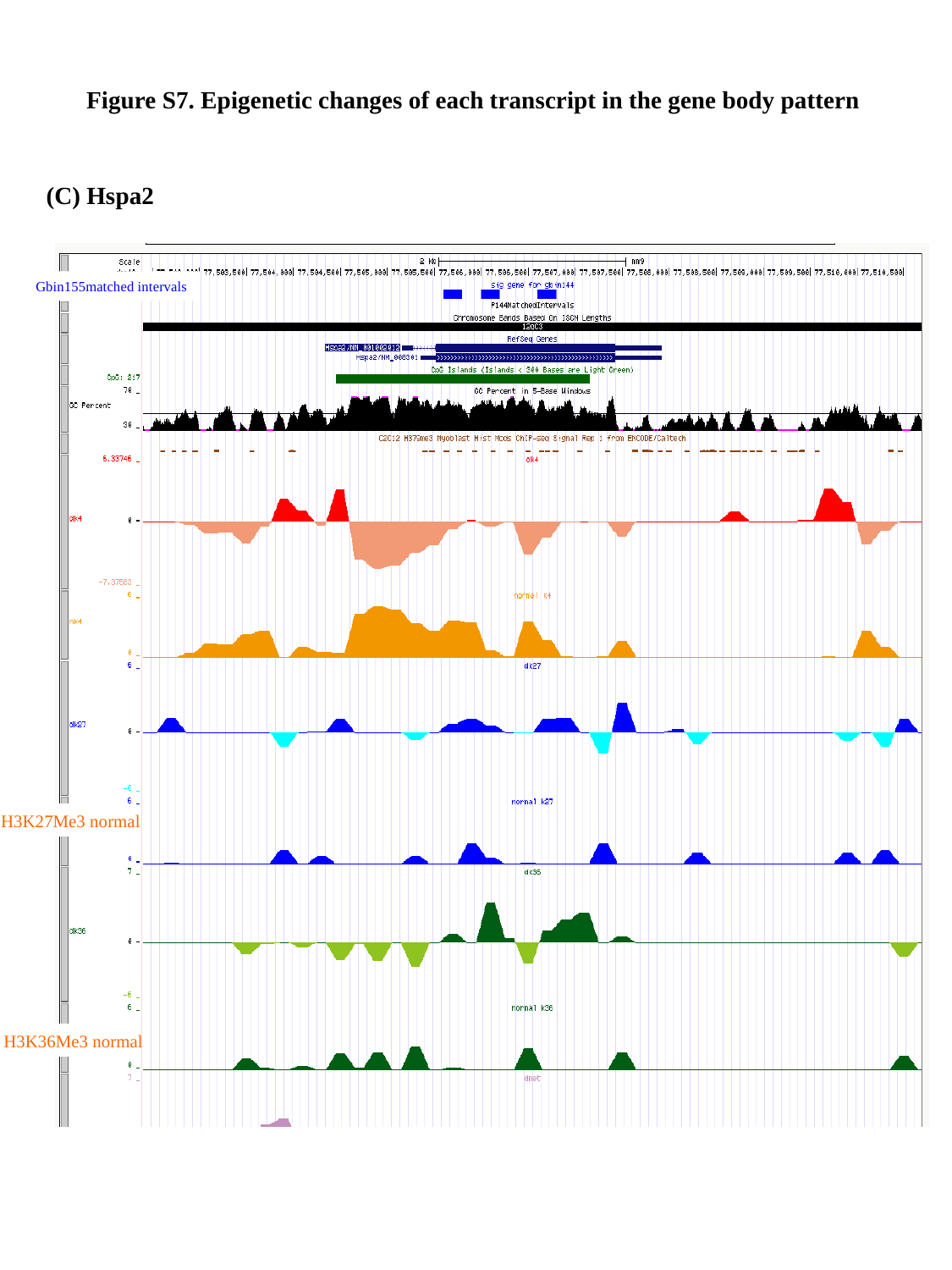

Figure S7. Epigenetic changes of each transcript in the gene body pattern
(C) Hspa2
Gbin155matched intervals
H3K27Me3 normal
H3K36Me3 normal

## Slide 13
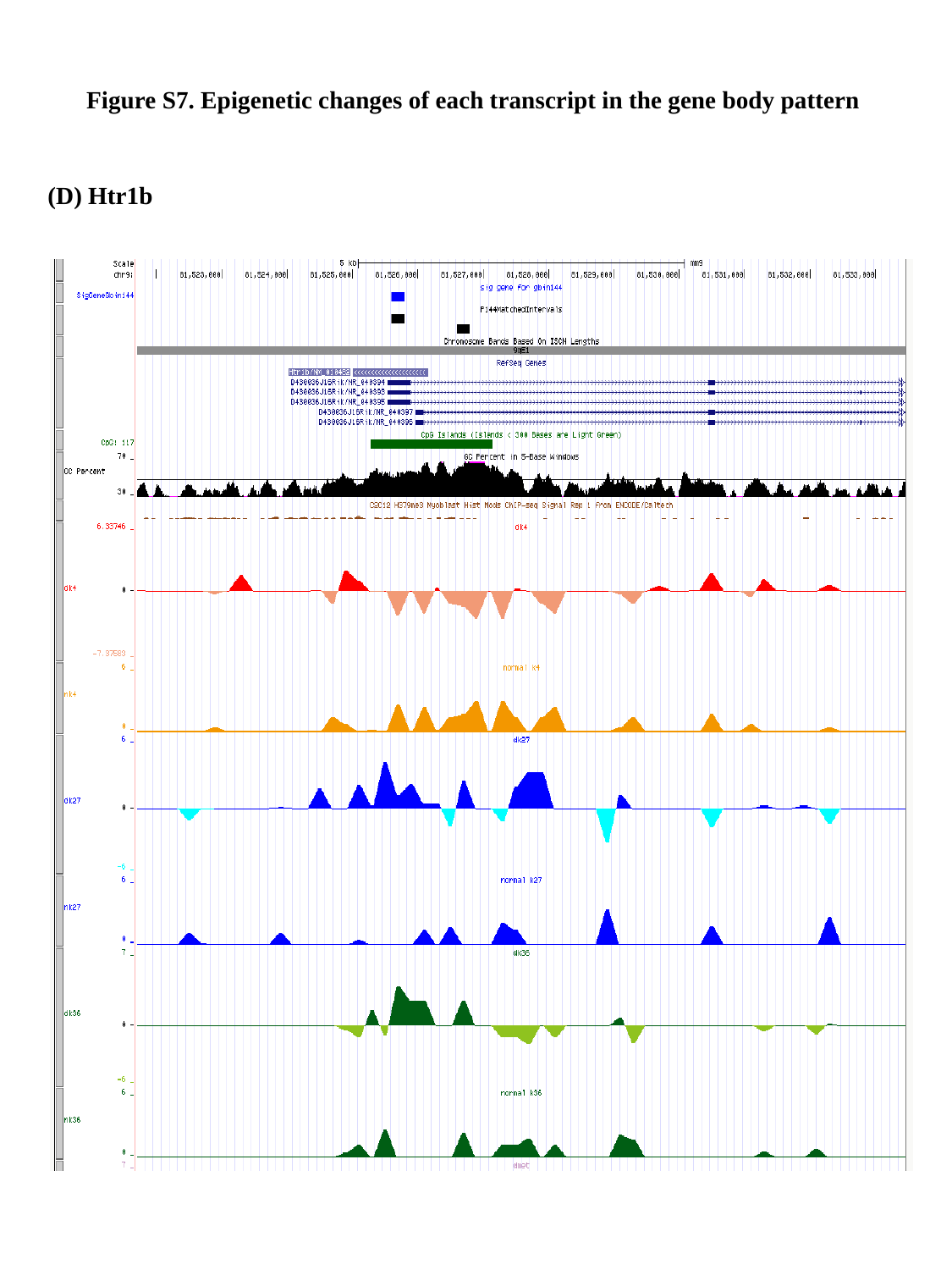

Figure S7. Epigenetic changes of each transcript in the gene body pattern
(D) Htr1b

## Slide 14
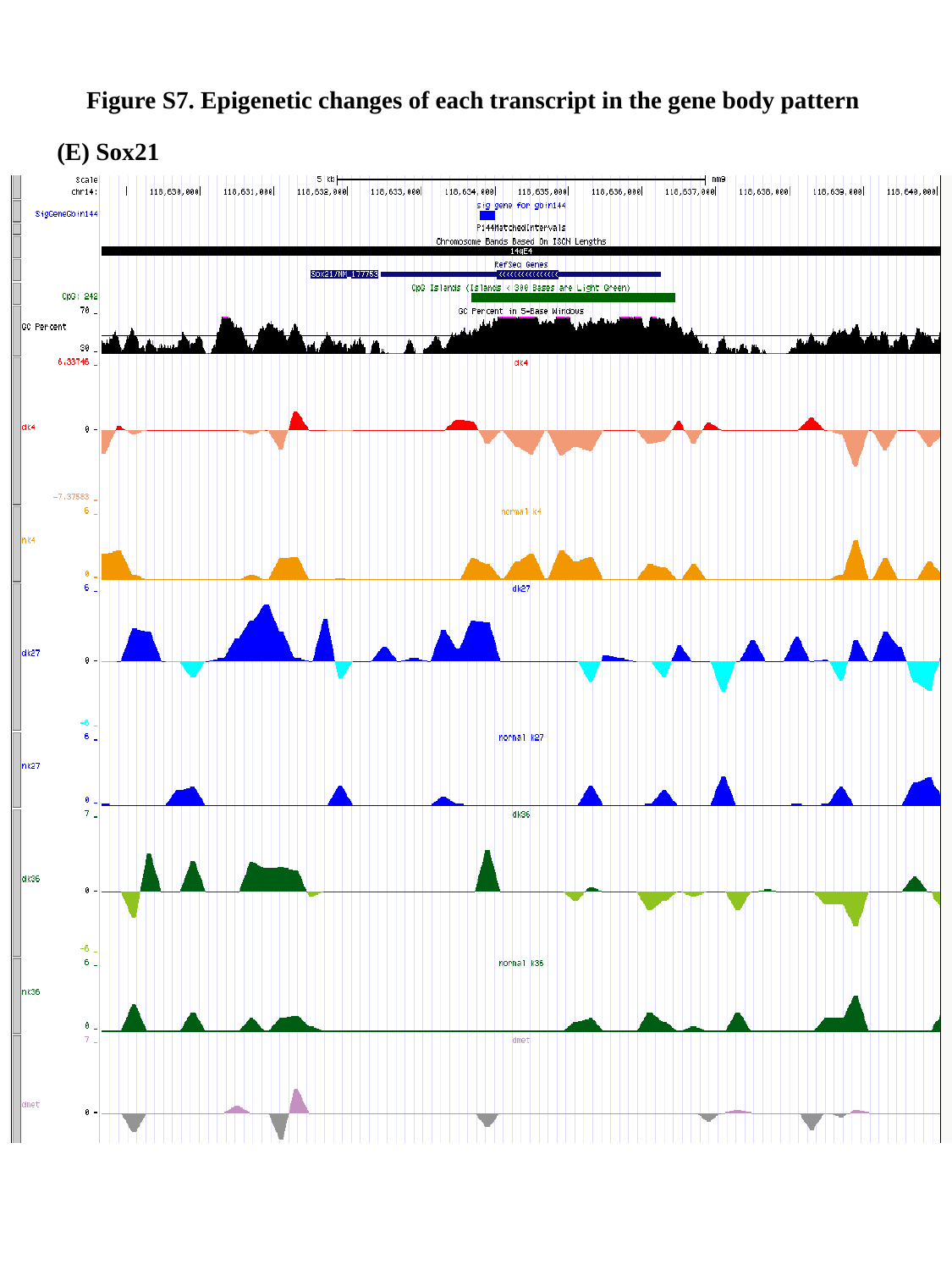

Figure S7. Epigenetic changes of each transcript in the gene body pattern
(E) Sox21

## Slide 15
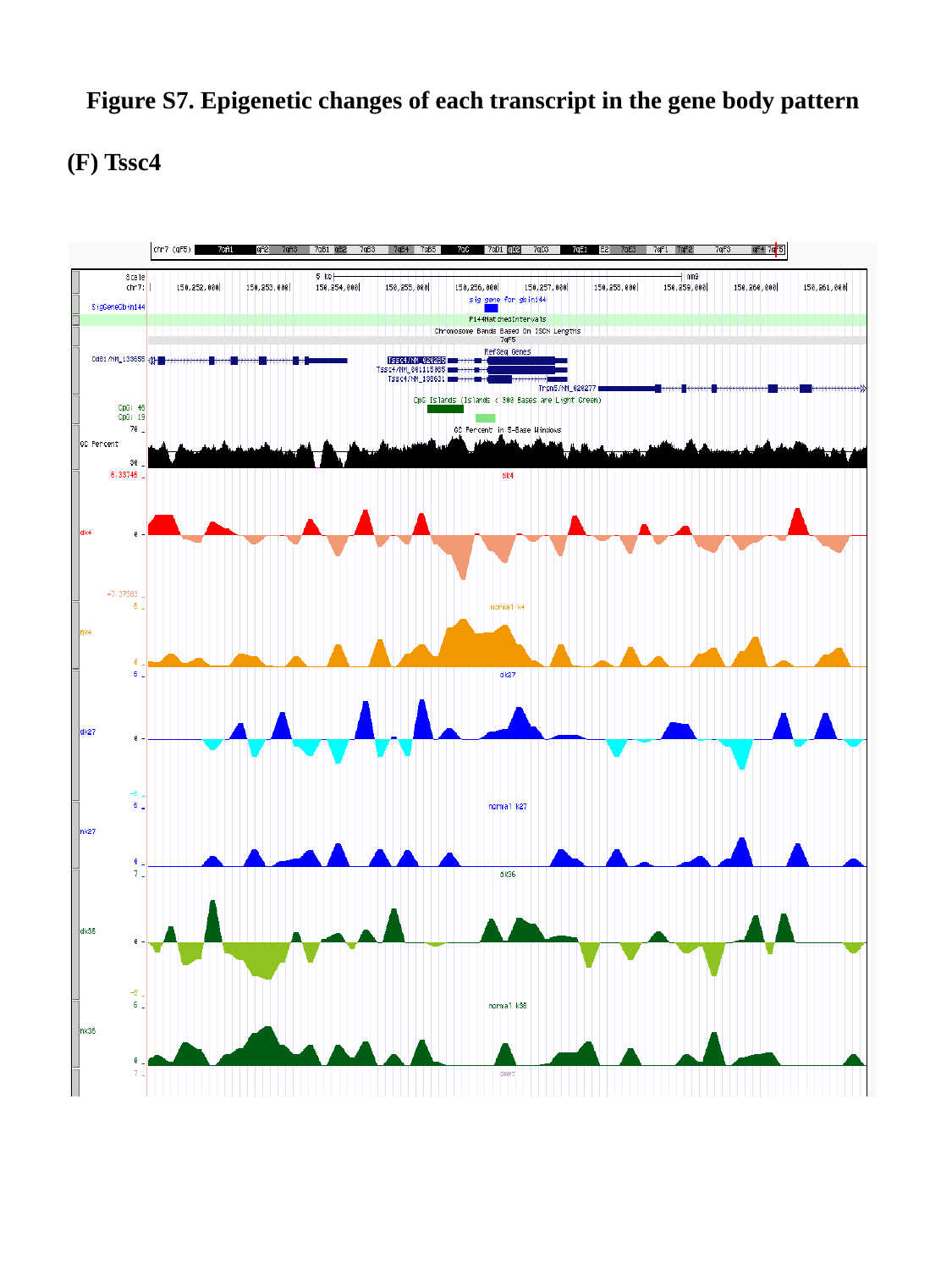

Figure S7. Epigenetic changes of each transcript in the gene body pattern
(F) Tssc4

## Slide 16
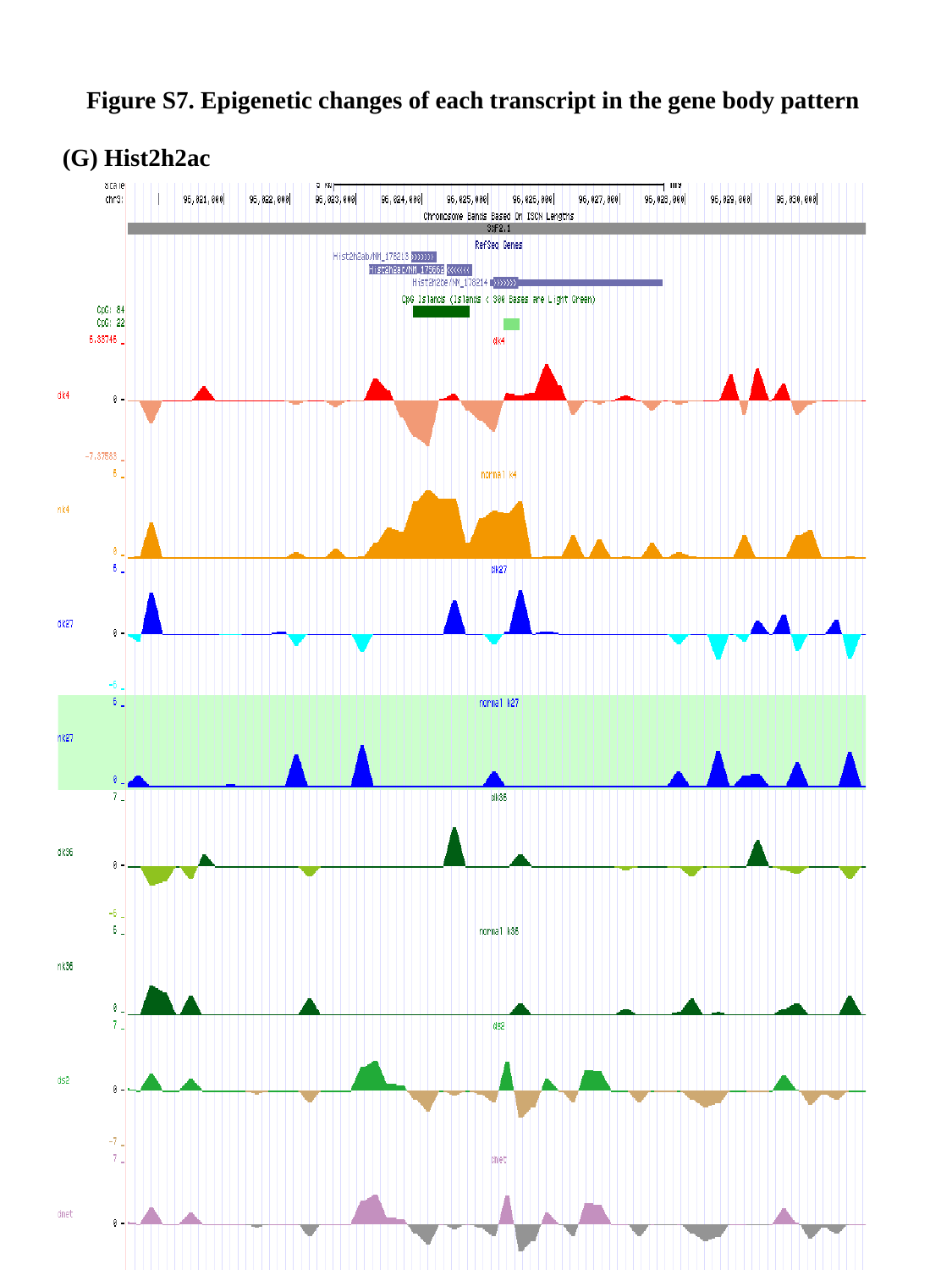

Figure S7. Epigenetic changes of each transcript in the gene body pattern
(G) Hist2h2ac
