# Supplementary material for: ChARM: Discovery of combinatorial chromatin modification patterns in hepatitis B virus X-transformed mouse liver cancer using association rule mining
Source: BMC Bioinformatics. 2016 Dec 13;17(Suppl 16):452. doi: 10.1186/s12859-016-1307-z (PMC5249029; doi:10.1186/s12859-016-1307-z)
Supplement: Additional file 6: — Comparison with ChAT. Figure S1. Pattern detected by ChAT on a ChIP-seq data set from livers of HBx TG mice. Figure S2. Multi-mode signatures composing of the same histone modifications. Figure S3. Pattern detected by ChAT on a ChIP-seq data set from livers of normal mice. Figure S4. Comparison of HBx with Normal: H3K4me3. Figure S5: Comparison of HBx with Normal: H3K36me3. Figure S6. Comparison of HBx with Normal: H3K27me3. (PPTX 909 kb) [file 12859_2016_1307_MOESM6_ESM.pptx]

## Slide 1
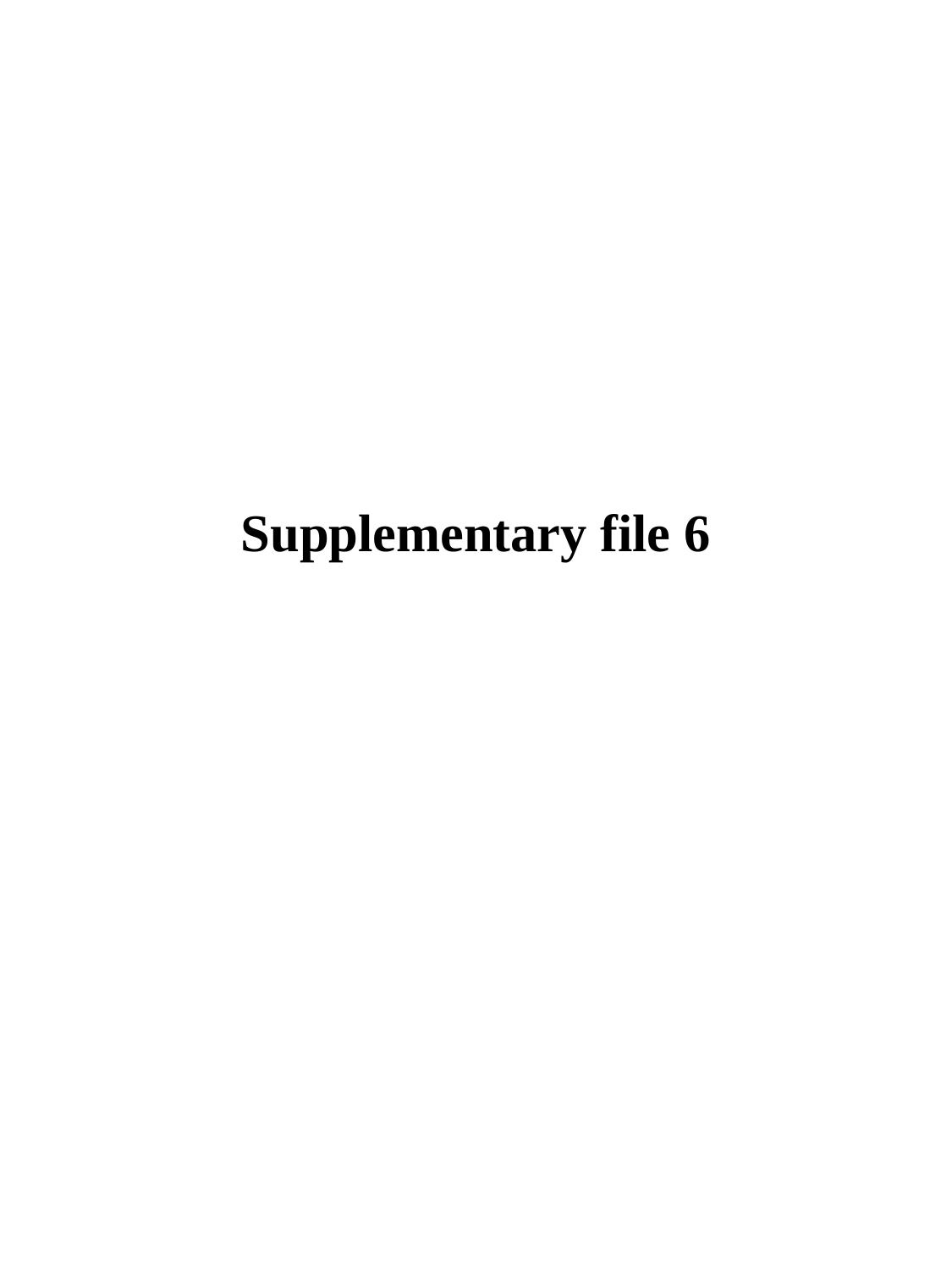

# Supplementary file 6

## Slide 2
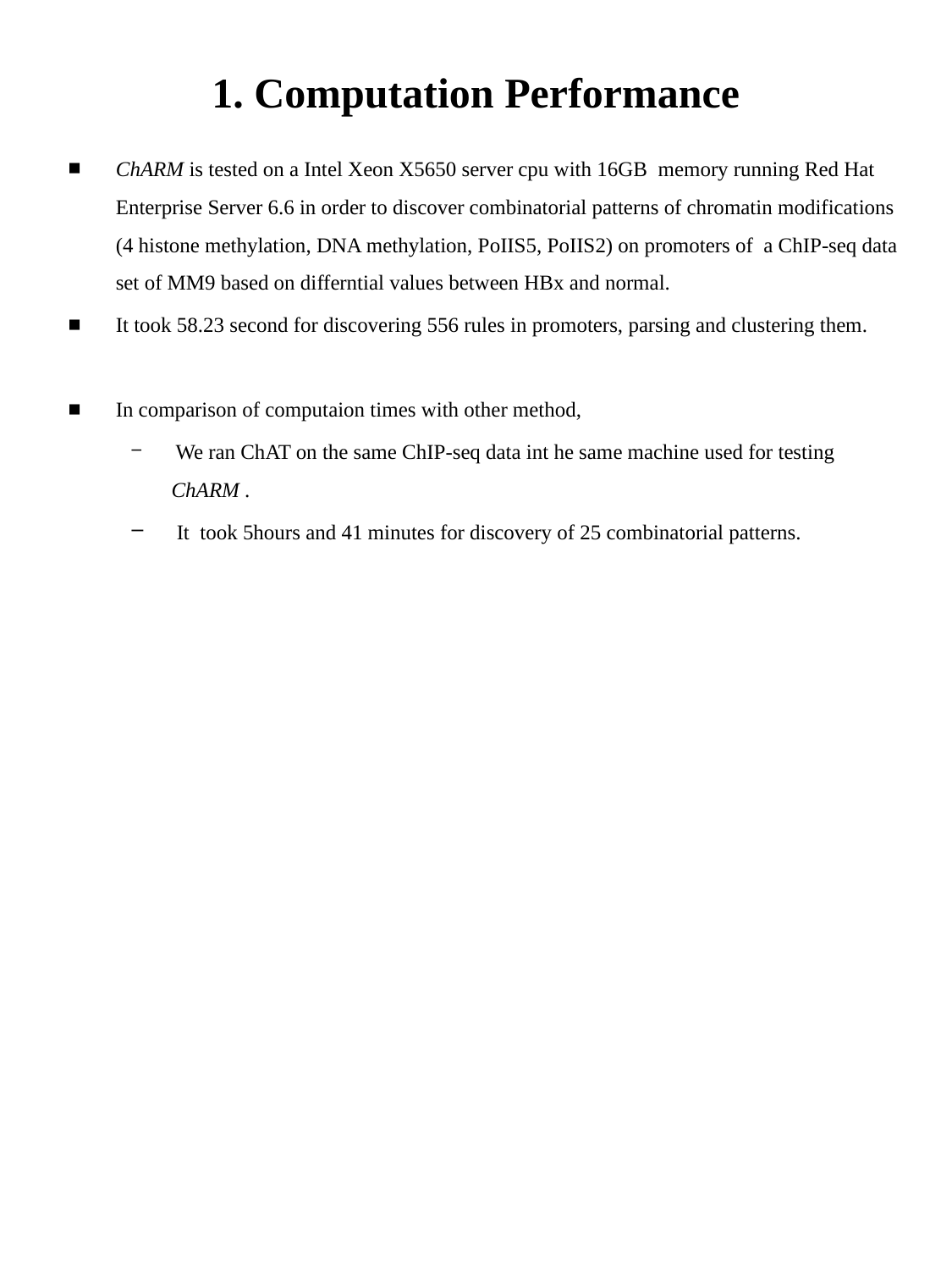

# 1. Computation Performance
ChARM is tested on a Intel Xeon X5650 server cpu with 16GB memory running Red Hat Enterprise Server 6.6 in order to discover combinatorial patterns of chromatin modifications (4 histone methylation, DNA methylation, PoIIS5, PoIIS2) on promoters of a ChIP-seq data set of MM9 based on differntial values between HBx and normal.
It took 58.23 second for discovering 556 rules in promoters, parsing and clustering them.
In comparison of computaion times with other method,
 We ran ChAT on the same ChIP-seq data int he same machine used for testing ChARM .
 It took 5hours and 41 minutes for discovery of 25 combinatorial patterns.

## Slide 3
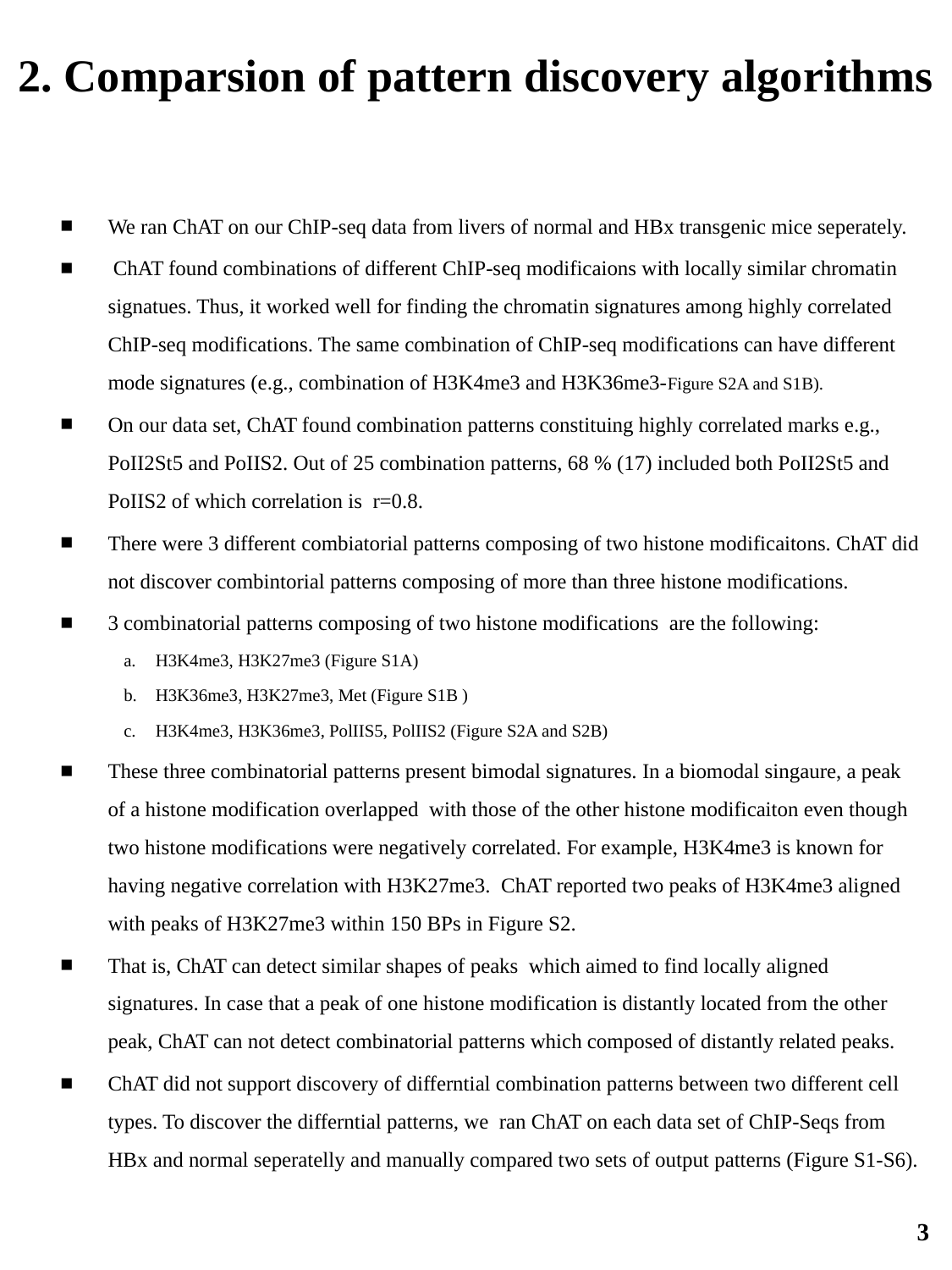

# 2. Comparsion of pattern discovery algorithms
We ran ChAT on our ChIP-seq data from livers of normal and HBx transgenic mice seperately.
 ChAT found combinations of different ChIP-seq modificaions with locally similar chromatin signatues. Thus, it worked well for finding the chromatin signatures among highly correlated ChIP-seq modifications. The same combination of ChIP-seq modifications can have different mode signatures (e.g., combination of H3K4me3 and H3K36me3-Figure S2A and S1B).
On our data set, ChAT found combination patterns constituing highly correlated marks e.g., PoII2St5 and PoIIS2. Out of 25 combination patterns, 68 % (17) included both PoII2St5 and PoIIS2 of which correlation is r=0.8.
There were 3 different combiatorial patterns composing of two histone modificaitons. ChAT did not discover combintorial patterns composing of more than three histone modifications.
3 combinatorial patterns composing of two histone modifications are the following:
H3K4me3, H3K27me3 (Figure S1A)
H3K36me3, H3K27me3, Met (Figure S1B )
H3K4me3, H3K36me3, PolIIS5, PolIIS2 (Figure S2A and S2B)
These three combinatorial patterns present bimodal signatures. In a biomodal singaure, a peak of a histone modification overlapped with those of the other histone modificaiton even though two histone modifications were negatively correlated. For example, H3K4me3 is known for having negative correlation with H3K27me3. ChAT reported two peaks of H3K4me3 aligned with peaks of H3K27me3 within 150 BPs in Figure S2.
That is, ChAT can detect similar shapes of peaks which aimed to find locally aligned signatures. In case that a peak of one histone modification is distantly located from the other peak, ChAT can not detect combinatorial patterns which composed of distantly related peaks.
ChAT did not support discovery of differntial combination patterns between two different cell types. To discover the differntial patterns, we ran ChAT on each data set of ChIP-Seqs from HBx and normal seperatelly and manually compared two sets of output patterns (Figure S1-S6).
3

## Slide 4
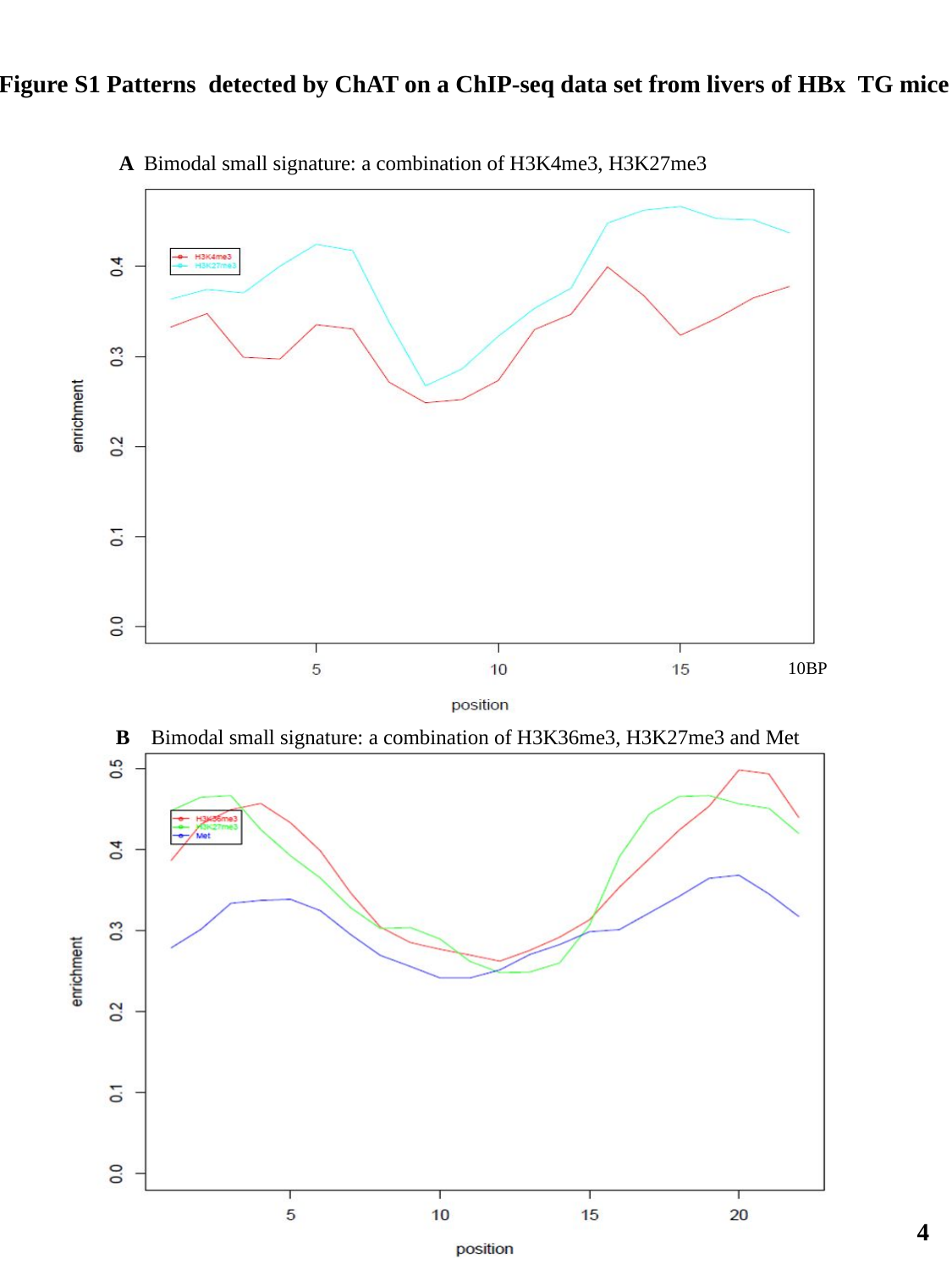

# Figure S1 Patterns detected by ChAT on a ChIP-seq data set from livers of HBx TG mice
 A Bimodal small signature: a combination of H3K4me3, H3K27me3
 10BP
 B Bimodal small signature: a combination of H3K36me3, H3K27me3 and Met
4

## Slide 5
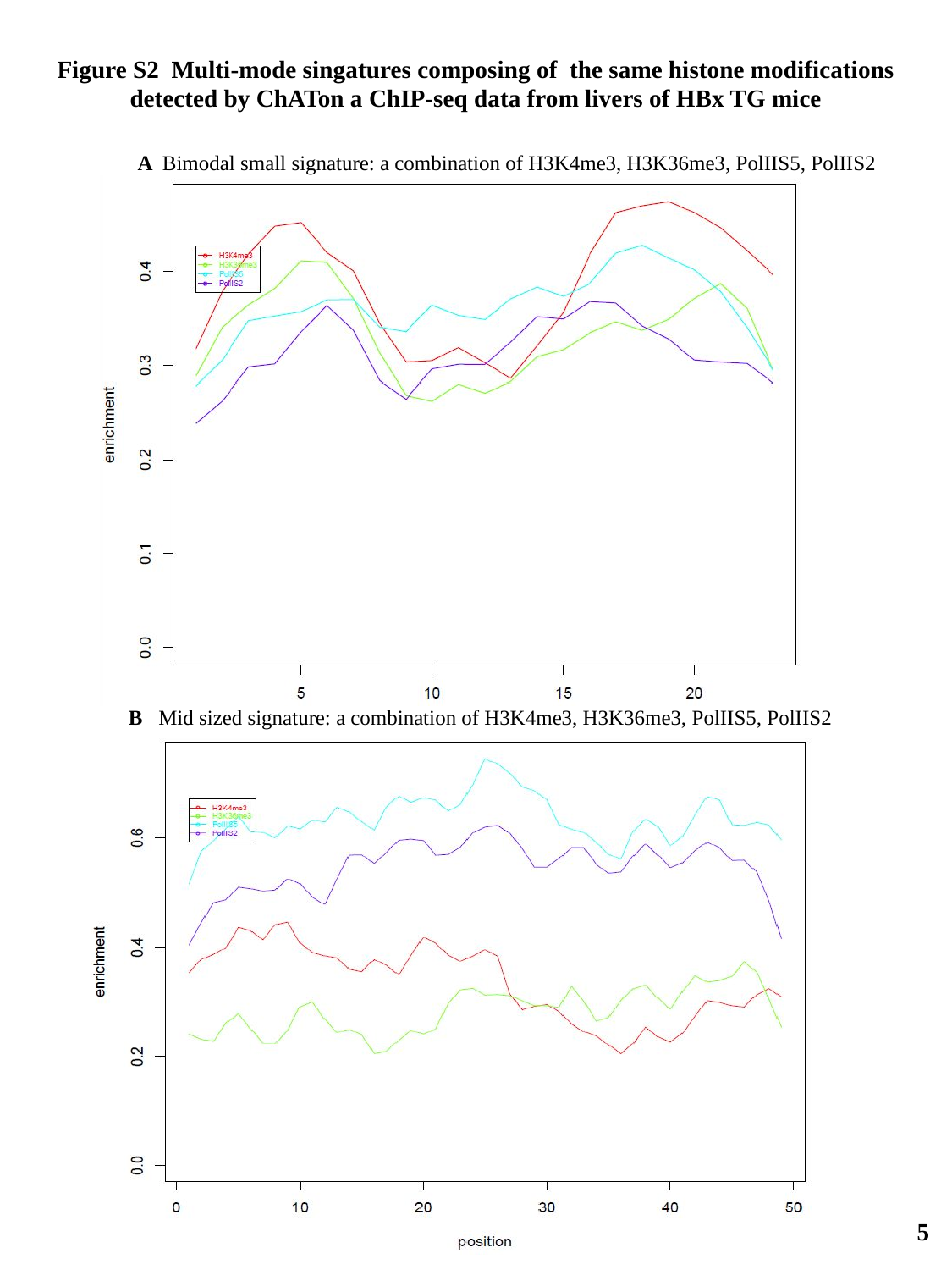

# Figure S2 Multi-mode singatures composing of the same histone modifications detected by ChATon a ChIP-seq data from livers of HBx TG mice
A Bimodal small signature: a combination of H3K4me3, H3K36me3, PolIIS5, PolIIS2
B Mid sized signature: a combination of H3K4me3, H3K36me3, PolIIS5, PolIIS2
5

## Slide 6
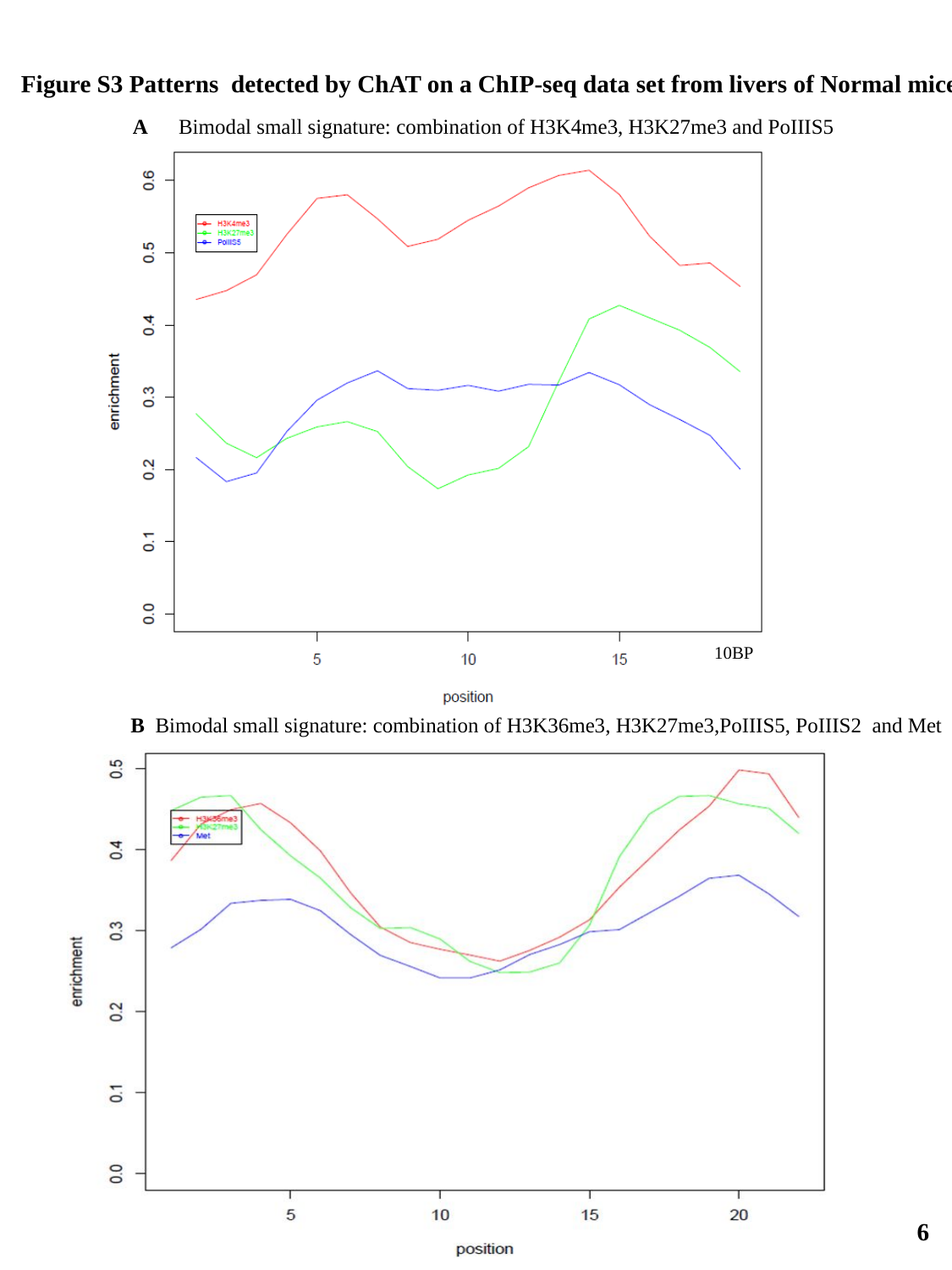

# Figure S3 Patterns detected by ChAT on a ChIP-seq data set from livers of Normal mice
 A Bimodal small signature: combination of H3K4me3, H3K27me3 and PoIIIS5
 10BP
 B Bimodal small signature: combination of H3K36me3, H3K27me3,PoIIIS5, PoIIIS2 and Met
6

## Slide 7
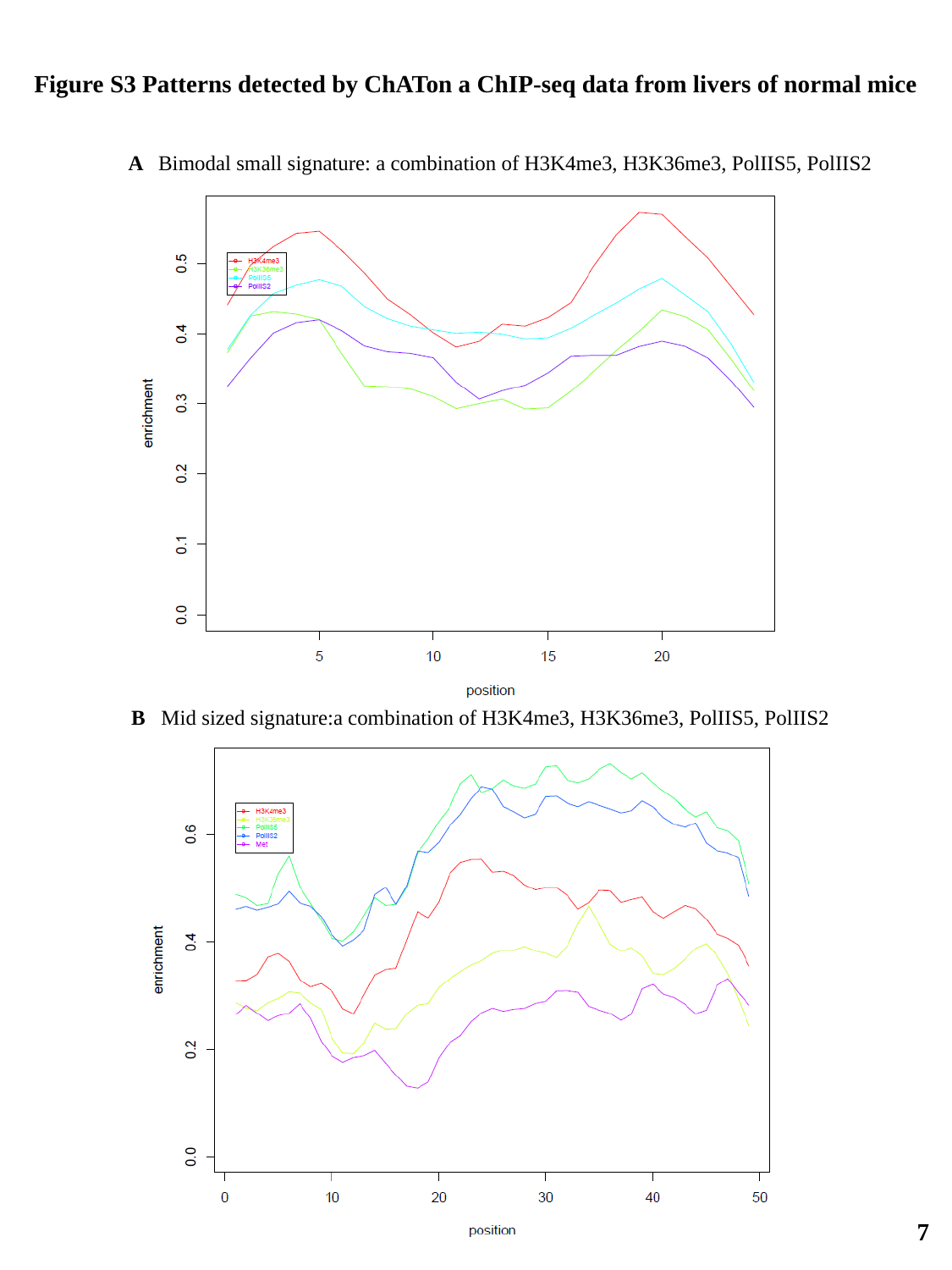

# Figure S3 Patterns detected by ChATon a ChIP-seq data from livers of normal mice
A Bimodal small signature: a combination of H3K4me3, H3K36me3, PolIIS5, PolIIS2
B Mid sized signature:a combination of H3K4me3, H3K36me3, PolIIS5, PolIIS2
7

## Slide 8
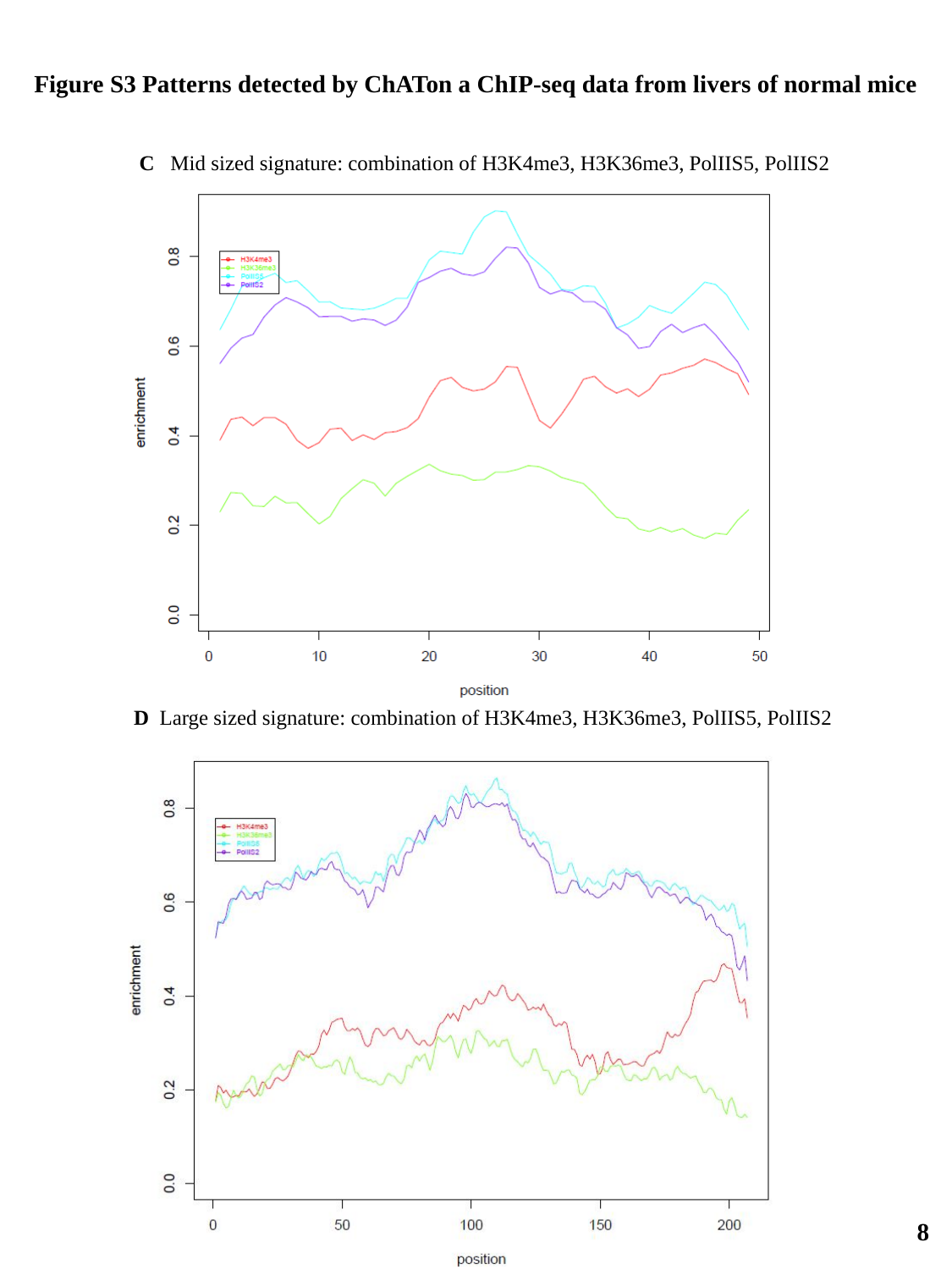

# Figure S3 Patterns detected by ChATon a ChIP-seq data from livers of normal mice
C Mid sized signature: combination of H3K4me3, H3K36me3, PolIIS5, PolIIS2
D Large sized signature: combination of H3K4me3, H3K36me3, PolIIS5, PolIIS2
8

## Slide 9
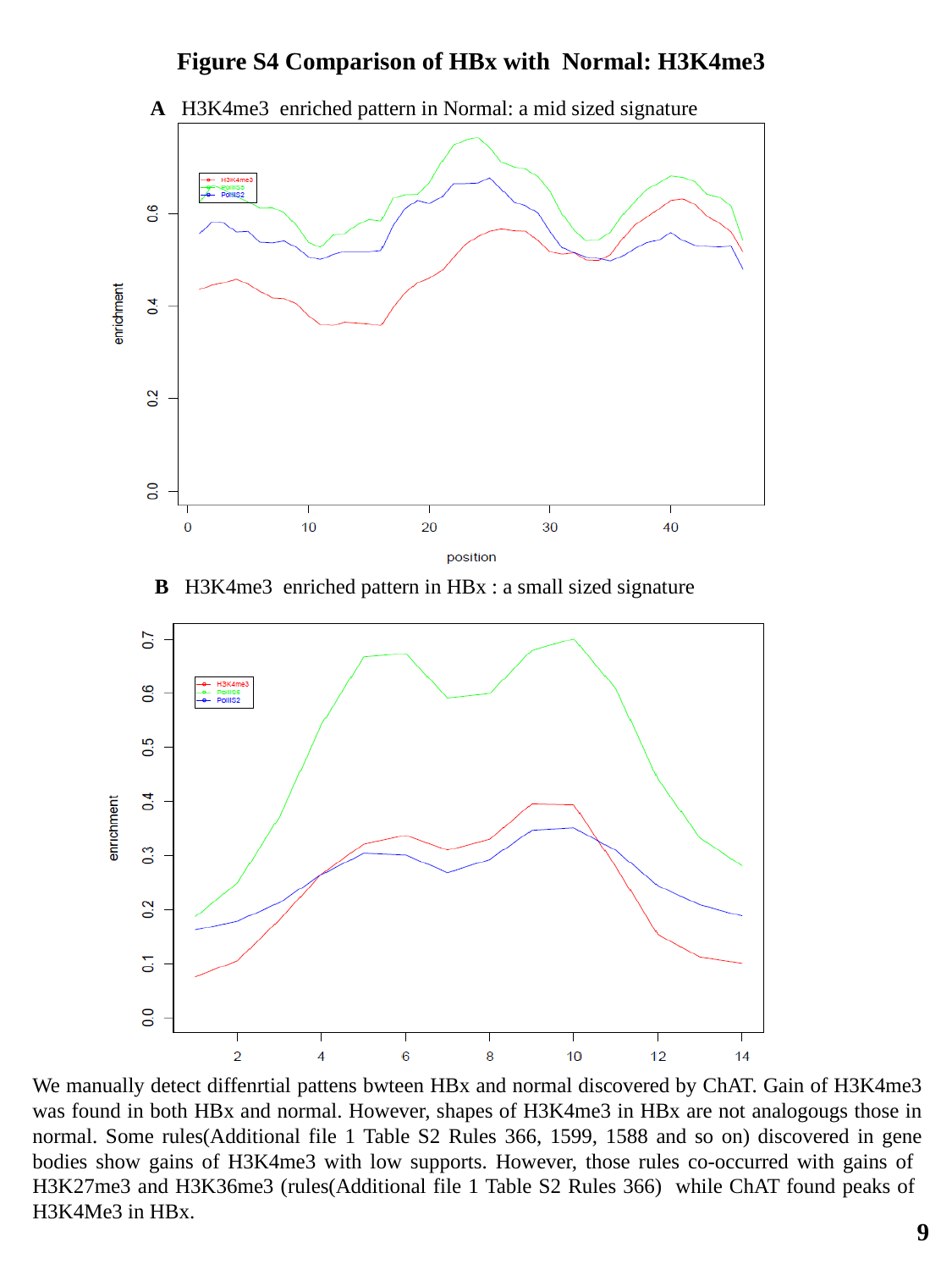

# Figure S4 Comparison of HBx with Normal: H3K4me3
A H3K4me3 enriched pattern in Normal: a mid sized signature
B H3K4me3 enriched pattern in HBx : a small sized signature
We manually detect diffenrtial pattens bwteen HBx and normal discovered by ChAT. Gain of H3K4me3 was found in both HBx and normal. However, shapes of H3K4me3 in HBx are not analogougs those in normal. Some rules(Additional file 1 Table S2 Rules 366, 1599, 1588 and so on) discovered in gene bodies show gains of H3K4me3 with low supports. However, those rules co-occurred with gains of H3K27me3 and H3K36me3 (rules(Additional file 1 Table S2 Rules 366) while ChAT found peaks of H3K4Me3 in HBx.
9

## Slide 10
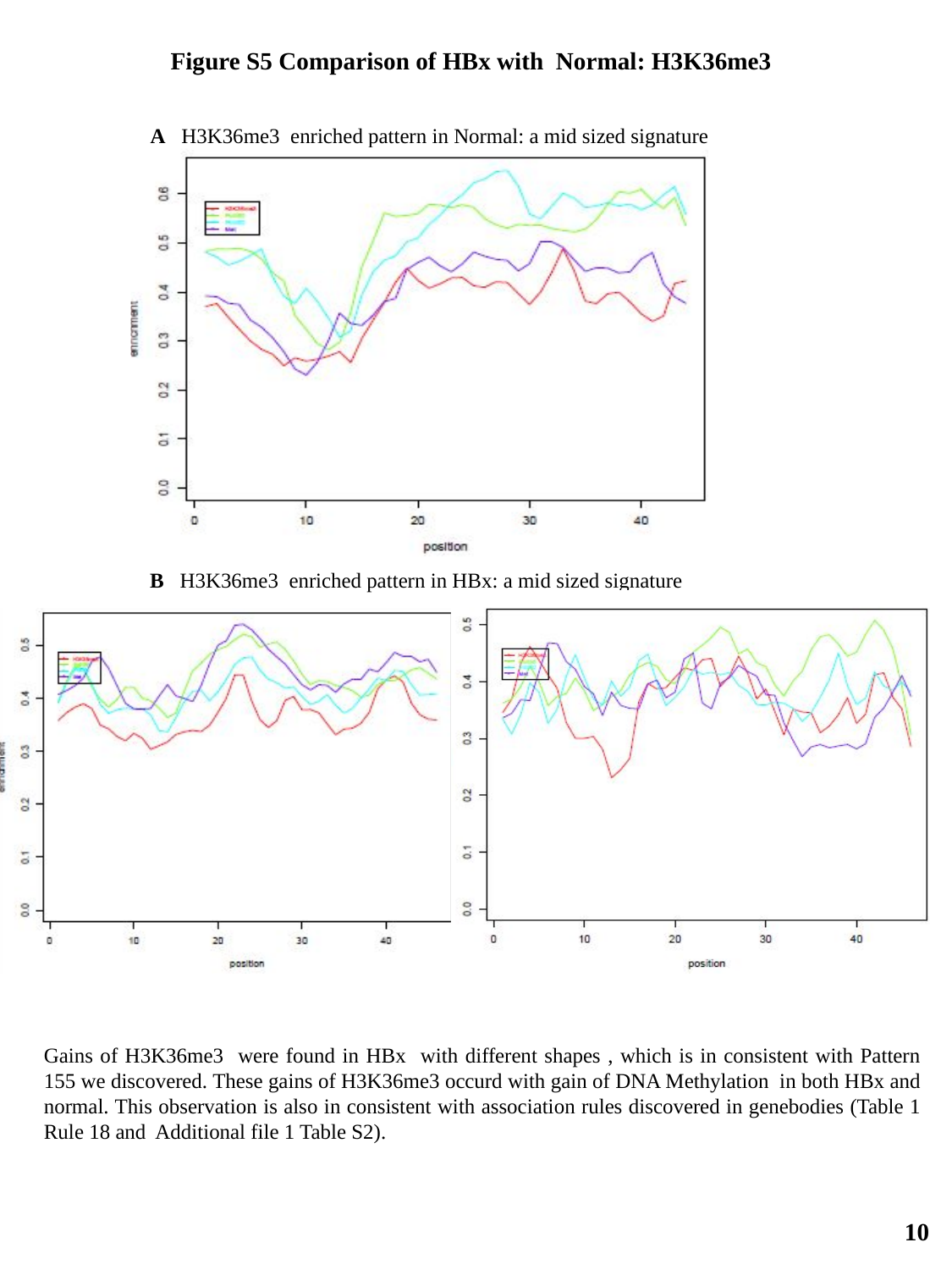

# Figure S5 Comparison of HBx with Normal: H3K36me3
A H3K36me3 enriched pattern in Normal: a mid sized signature
B H3K36me3 enriched pattern in HBx: a mid sized signature
Gains of H3K36me3 were found in HBx with different shapes , which is in consistent with Pattern 155 we discovered. These gains of H3K36me3 occurd with gain of DNA Methylation in both HBx and normal. This observation is also in consistent with association rules discovered in genebodies (Table 1 Rule 18 and Additional file 1 Table S2).
10

## Slide 11
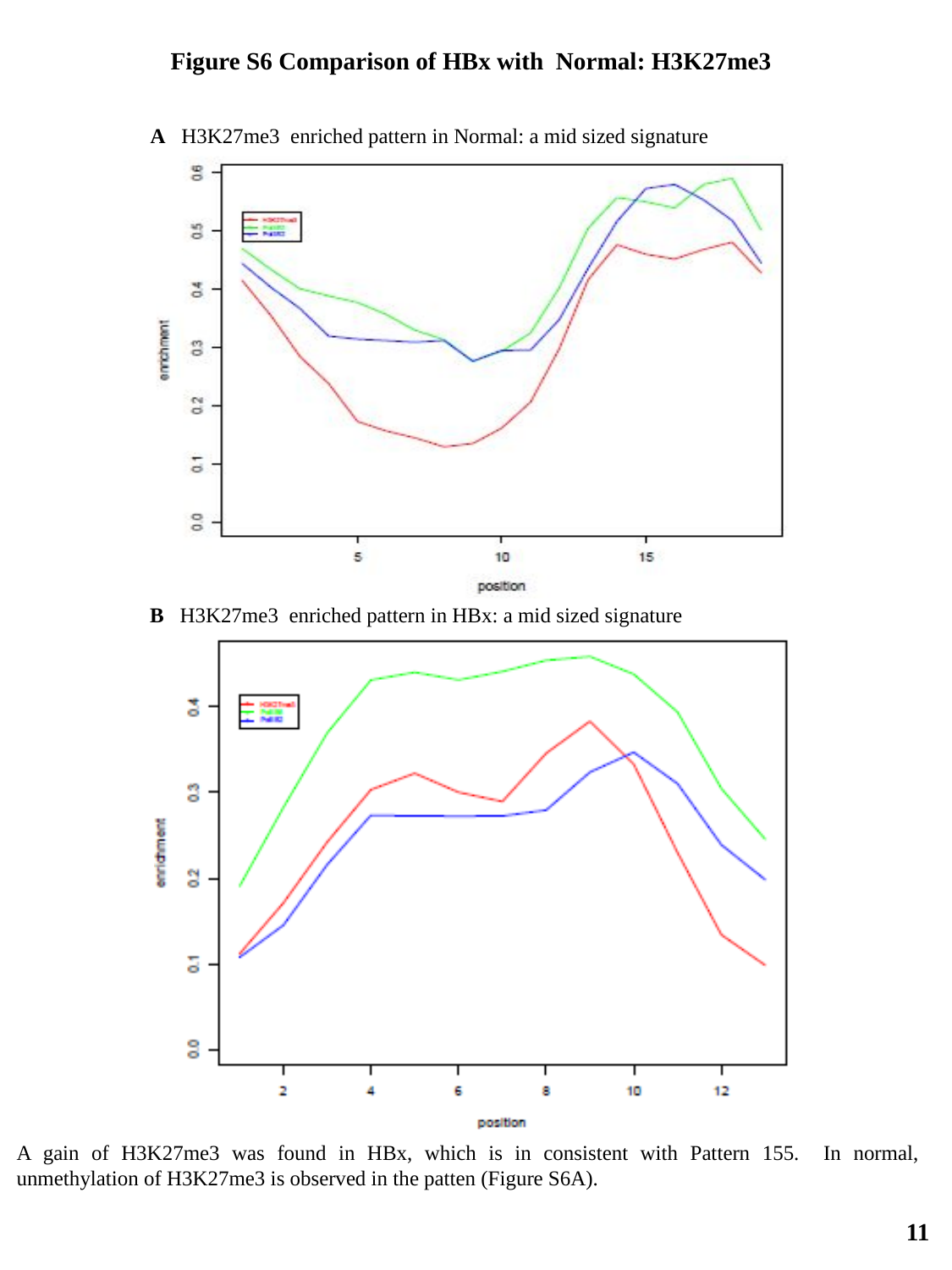

# Figure S6 Comparison of HBx with Normal: H3K27me3
A H3K27me3 enriched pattern in Normal: a mid sized signature
B H3K27me3 enriched pattern in HBx: a mid sized signature
A gain of H3K27me3 was found in HBx, which is in consistent with Pattern 155. In normal, unmethylation of H3K27me3 is observed in the patten (Figure S6A).
11
